# Molecular Evolution and Functional Diversification of Replication Protein A1 in Plants

**DOI:** 10.3389/fpls.2016.00033

**Published:** 2016-01-29

**Authors:** Behailu B. Aklilu, Kevin M. Culligan

**Affiliations:** ^1^Department of Molecular, Cellular and Biomedical Sciences, University of New HampshireDurham, NH, USA; ^2^Program in Genetics, University of New HampshireDurham, NH, USA

**Keywords:** RPA, replication, meiosis, DNA repair

## Abstract

Replication protein A (RPA) is a heterotrimeric, single-stranded DNA binding complex required for eukaryotic DNA replication, repair, and recombination. RPA is composed of three subunits, RPA1, RPA2, and RPA3. In contrast to single RPA subunit genes generally found in animals and yeast, plants encode multiple paralogs of RPA subunits, suggesting subfunctionalization. Genetic analysis demonstrates that five *Arabidopsis thaliana* RPA1 paralogs (RPA1A to RPA1E) have unique and overlapping functions in DNA replication, repair, and meiosis. We hypothesize here that RPA1 subfunctionalities will be reflected in major structural and sequence differences among the paralogs. To address this, we analyzed amino acid and nucleotide sequences of *RPA1* paralogs from 25 complete genomes representing a wide spectrum of plants and unicellular green algae. We find here that the plant *RPA1* gene family is divided into three general groups termed *RPA1A, RPA1B*, and *RPA1C*, which likely arose from two progenitor groups in unicellular green algae. In the family *Brassicaceae* the *RPA1B* and *RPA1C* groups have further expanded to include two unique sub-functional paralogs *RPA1D* and *RPA1E*, respectively. In addition, *RPA1* groups have unique domains, motifs, *cis*-elements, gene expression profiles, and pattern of conservation that are consistent with proposed functions in monocot and dicot species, including a novel C-terminal zinc-finger domain found only in plant RPA1C-like sequences. These results allow for improved prediction of RPA1 subunit functions in newly sequenced plant genomes, and potentially provide a unique molecular tool to improve classification of *Brassicaceae* species.

## Introduction

Replication protein A (RPA) is a heterotrimeric, single-stranded DNA (ssDNA) binding protein complex that is highly conserved in eukaryotes (Wold, 1997). It was first identified as an indispensable component for the *in vitro* replication of simian virus 40 (SV40) DNA (Wobbe et al., 1987). Its primary function is to protect ssDNA from nucleolytic damage and hairpin formation during DNA metabolism. Consistent with its function, RPA plays essential roles in almost all DNA metabolic pathways including DNA replication, transcription, recombination, DNA damage surveillance and recognition, cell-cycle checkpoints, and in all major types of DNA repair including base excision, nucleotide excision, mismatch, and double-strand break repair (Wold, 1997; Iftode et al., 1999; Zou et al., 2006). RPA participates in these diverse DNA metabolic pathways through its ability to interact with DNA and numerous proteins involved in these processes (Iftode et al., 1999; Zou et al., 2006). In both animal and yeast (*Saccharomyces cerevisiae*) cells, RPA is hyper-phosphorylated upon DNA damage or replication stress by checkpoint kinases including ATM, ATR, and DNA-PK (Binz et al., 2004; Vassin et al., 2004; Liu et al., 2006). The hyper-phosphorylation may switch the function of RPA from DNA replication to DNA repair by inducing changes in the RPA structure and RPA-DNA and RPA–protein interactions, suggesting that animal and yeast cells use a structure-based modulation mechanism of RPA (Binz et al., 2004; Vassin et al., 2004).

Although DNA metabolism and DNA damage responses are highly conserved in eukaryotes, RPA regulation in plants appears different from animals and yeast. For example, RPA hyper-phosphorylation was not detected in rice (*Oryza sativa*) plants treated with DNA damaging agents (Marwedel et al., 2003). Furthermore, in contrast to the single RPA1, RPA2, and RPA3 subunits of the heterotrimeric RPA complex found in yeast and mammals (except for few mammals with a second RPA2), plants encode multiple RPA1, RPA2, and RPA3 subunits. Rice encodes three *RPA1*-like genes (*RPA1a,b,c*), three *RPA2*-like genes (*RPA2-1,-2,-3*), and one *RPA3*-like gene (Ishibashi et al., 2006). Arabidopsis (*Arabidopsis thaliana*) encodes five *RPA1* genes (*RPA1-A, B, C, D*, and *E*; Shultz et al., 2007; Aklilu et al., 2014), two RPA2 genes, and two RPA3 genes (Ganpudi and Schroeder, 2011).

Why do plants contain multiple functional *RPA1* gene paralogs? Paralogous genes are generated by events such as whole-genome duplication, segmental duplication, and tandem gene duplication. Unlike animals, genome duplication is prominent in plants (Lockton and Gaut, 2005; Cui et al., 2006). For example, Arabidopsis has experienced at least three events of whole genome duplications (Vision et al., 2000; Simillion et al., 2002). There are three possible fates of genes after duplication. These are (i) nonfunctionalization/pseudogenization or loss, where one copy loses its function(s) by degenerative mutations, (ii) subfunctionalization where both copies become partially compromised by mutation accumulation to the point at which their total capacity is reduced to the level of the single-copy ancestral gene, and (iii) neofunctionalization where one copy acquires a novel, beneficial function and become preserved by natural selection, with the other copy retaining the original function (Lynch and Conery, 2000; Zhang, 2003; Moore and Purugganan, 2005; Louis, 2007). The subfunctionalization model also predicts that duplicate genes will share overlapping redundant functions early in the process of functional divergence (Moore and Purugganan, 2005).

Using functional genetic analysis we previously showed that all of the five Arabidopsis *RPA1* paralogs are functional (Aklilu et al., 2014). We also reported that these paralogs have undergone subfunctionalization but also share different degrees of overlapping redundant functions. For instance, the *rpa1c* mutant displays hypersensitivity to DNA double-strand breaks induced by both ionizing radiation and camptothecin, while the *rpa1e* mutant shows hypersensitivity only to ionizing radiation. Combination of *rpa1c* and *rpa1e* results in additive hypersensitivity to a variety of DNA damaging agents. Overall, the results suggest that RPA1C and RPA1E each play unique roles in the repair of DNA damage, with RPA1C playing a leading role in promoting double-strand break repair (Aklilu et al., 2014).

Conversely, the *rpa1b rpa1d* double mutant has a severely defective growth and developmental phenotype (reduced fitness) under normal growth conditions. However, it displays similar sensitivity as wild-type plants for DNA damaging agents. The growth and developmental abnormalities of the *rpa1b rpa1d* double mutant are likely a result of defects in DNA replication that generate abnormal cell division suggesting both RPA1B and RPA1D are required for normal DNA replication (Aklilu et al., 2014).

In addition to DNA repair and DNA replication activities, RPA1 proteins play essential roles in progression of meiosis. Of all the five *rpa1* single mutants, only the *rpa1a* single mutant displays an obvious defective meiotic phenotype, observed as lower seed set and reduced crossover formation (Osman et al., 2009; Aklilu et al., 2014). However, the *rpa1a rpa1c* double mutant is completely sterile, with incomplete synapsis and chromosome fragmentation observed during meiosis (Aklilu et al., 2014). This suggests that both RPA1A and RPA1C play important and perhaps overlapping roles during meiosis.

In order to understand the evolutionary history and identify specific sequence variations responsible for the functional diversification of RPA1 throughout plants, we have analyzed the nucleotide and amino acid sequences, gene conservation, and structural features of all available plant RPA1 paralogs. The sequence and evolutionary analysis together with our new additional genetic analysis reveals that plant *RPA1* gene family is divided into three main evolutionary groups (A, B, and C): Group A (including *A. thaliana* RPA1A) is primarily responsible for meiotic progression. Group B (including *A. thaliana* RPA1B and RPA1D) is responsible for normal DNA replication. Group C (including *A. thaliana* RPA1C and RPA1E) is primarily responsible for DNA damage repair. As we show here, these groups have unique coding and regulatory sequences, gene and protein structure, and different level of gene conservation and expression that are consistent with these proposed functions, and highlight how evolution of this gene family occurred into specialized members. These data provide needed sequence structure context for future biochemical studies of RPA function in plants.

## Materials and methods

### Plant materials and growth

Plant growth conditions and plant lines, including Wild-type (Wt) and all *RPA1* single mutant lines used are previously described (Aklilu et al., 2014).

### Microscopy

Meiotic chromosome spreads were prepared as described (Armstrong et al., 2001). Slides were mounted with DAPI (2.5 mg/ml) in Vectashield. Chromosomes were visualized with a confocal microscope (Zeiss LSM 510) using the halogen light. Images were then captured using an externally mounted digital camera (Olympus CKX41) and processed with a SPOT microscope imaging software.

### Sequences sources

Promoter, coding, and amino acid sequences were obtained from the National Center for Biotechnology Information (NCBI) and The Arabidopsis Information Resources (TAIR) database. Orthologous RPA1 sequences were identified by employing NCBI BLAST searches. Full length Arabidopsis RPA1 amino acid sequences were used for the BLAST search against the genome of each organism. In addition, sequences identified at each step are used to refine the search. Orthology was confirmed by reverse BLAST. The lowest *e*-value and maximum percent identity BLAST hit was chosen as the putative ortholog of the respective Arabidopsis RPA1 protein.

### Phylogenetic analysis

Phylogenetic analysis of the RPA1 protein sequences was performed with the MEGA5 software package (Tamura et al., 2011). Amino acid sequences were aligned using ClustalW (default parameters) with in the MEGA5 software package. Trees were constructed using maximum likelihood methods with Jones-Taylor-Thornton (JTT) amino acid substitution model and 1000 bootstrap replicates. Except from bootstrapping and choice of model, all other parameters were left at default settings. Additional neighbor joining (NJ) trees were constructed with the same amino acid substitution model, bootstrap replicates, and NJ tree construction default parameters in MEGA5 to confirm the results of the ML analysis.

### Codon bias/usage

Frequency of optimal codons (F_OP_) was calculated based on optimal codons identified for Arabidopsis (Wright et al., 2004) and rice (Liu et al., 2004).

### Gene structure and protein domains

Introns in *RPA1* genes were identified using the NCBI gene database. RPA1 protein domains and subdomains were identified using the NCBI Conserved Domain Database [CDD] (Marchler-Bauer et al., 2011).

### Sequence distance and natural selection

Analysis of synonymous (*dS*) and nonsysnonymous (*dN*) substitution rate were performed with the MEGA5 software package using the Nei-Gojobori substitution model (Tamura et al., 2011). Natural selection (ω) was calculated by dividing *dN* by *dS* (*dN*/*dS*).

### Gene expression

Level and pattern of *RPA1* genes expression at different developmental stages and tissues were retrieved from genevestigator (https://www.genevestigator.com) and Arabidopsis eFP browser (http://bar.utoronto.ca/efp/cgi-bin/efpWeb.cgi). Genevestigator is an online visualization tool that summarizes results from thousands of high quality transcriptomic experiments often done by cDNA microarrays (Hruz et al., 2008). Arabidopsis eFP browser is also a data visualization tool for exploring degree and location of gene expression (Winter et al., 2007).

### Analysis of *cis-elements* in arabidopsis *RPA1* promoter regions

Promoter elements for all the sequences were analyzed using the PLACE (Plant Cis-acting Regulatory DNA Elements) database (http://www.dna.affrc.go.jp/PLACE/; Higo et al., 1999).

### Arabidopsis *RPA1* TAIR gene codes

AtRPA1A, AT2G06510; AtRPA1B, AT5G08020; AtRPA1C, AT5G45400; AtRPA1D, AT5G61000; AtRPA1E, AT4G19130.

## Results and discussion

### Arabidopsis *RPA1A* plays a leading role in meiosis

**Figure 1:**
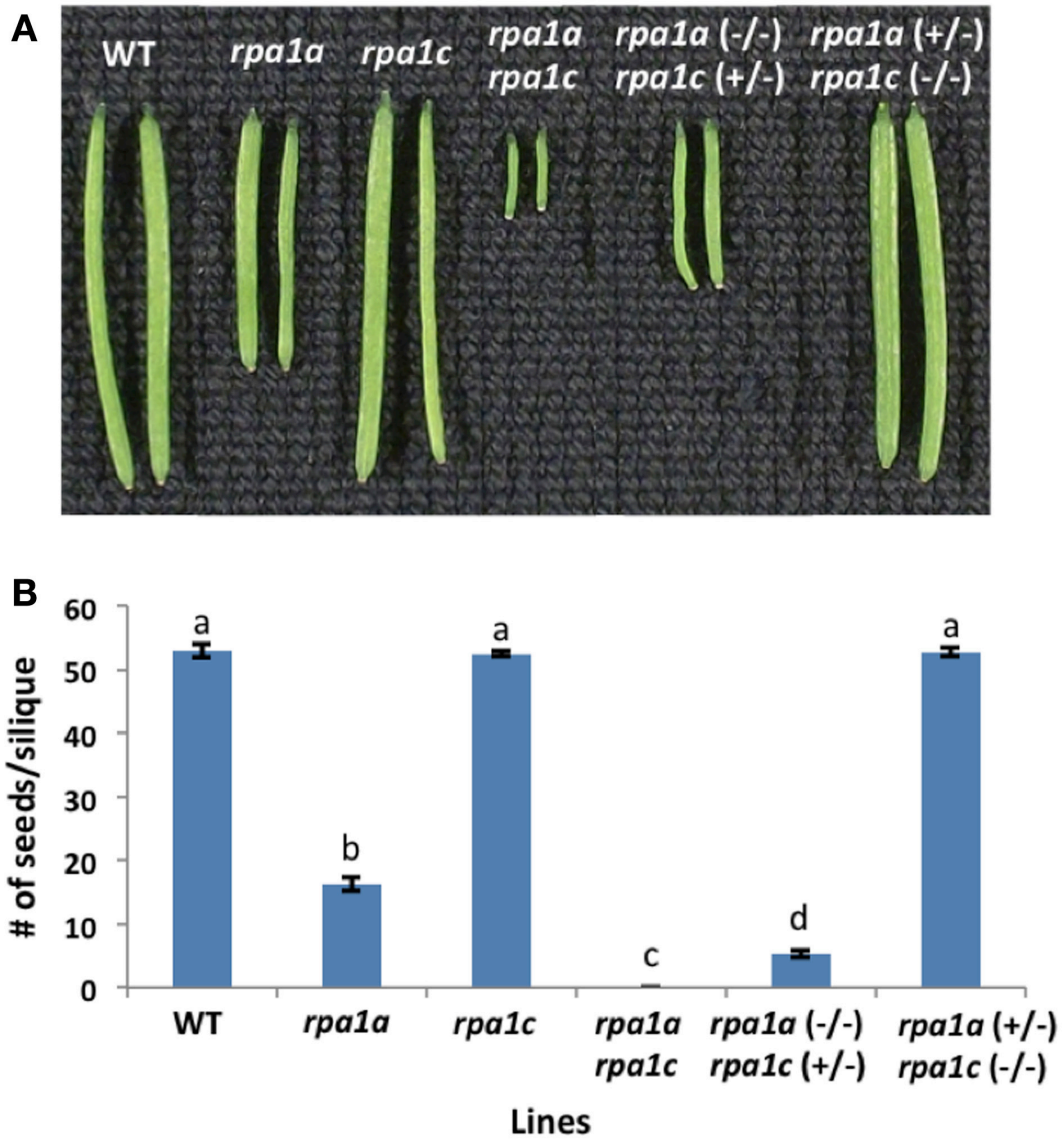
**Meiotic defective ***A. thaliana*** RPA1 mutant lines. (A)** Siliques harvested from ~7 weeks old wild-type (WT) and RPA1 mutant plants. **(B)** Number of seeds per silique. The mutant plants have the following percentage of fertility reduction: *rpa1a*: ~70%; *rpa1a* (−/−) *rpa1c* (+/−): ~92%; *rpa1a rpa1c*: 100%. *rpa1a* (+/−) *rpa1c* (−/−) has similar fertility level as WT. Data are mean ± SE (*n* > 10). To analyze statistical difference *F*-test (ANOVA) and LSD were carried out at *P* ≤ 0.05. Bars with different letters indicate significant differences.

We previously described the role of RPA1A and RPA1C during meiotic progression employing homozygous mutants *rpa1a, rpa1c*, and the double mutant *rpa1a rpa1c* (Aklilu et al., 2014). These results suggest a genetically redundant role for both RPA1A and RPA1C during early stages of meiosis (crossing over and repair). A previous study suggests RPA1A plays a unique role during later stages of meiosis (second-end capture; Osman et al., 2009), but there is currently no evidence to support a role for RPA1C at later stages of meiosis. To determine which if any of these two subunits plays a more predominant role during early (repair) stages of meiosis (and thereby providing additional clarity into the functional classification of these genes), we generated two additional heterozygous mutant combinations, *rpa1a* (+/−) *rpa1c* (−/−) and conversely *rpa1a* (−/−) *rpa1c* (+/−). By comparing these two combinations, we sought to determine the relative dominance (“gene dosage”) of each in promoting early meiotic repair. As shown in Figures 1A,B, phenotypic analysis of these mutant combinations show that while *rpa1a* (+/−) *rpa1c* (−/−) is fully fertile, the *rpa1a* (−/−) *rpa1c* (+/−), displays reduced fertility (~92% reduction in fertility) vs. the *rpa1a* (−/−) single mutant.

To evaluate meiotic integrity at the chromosomal level, we prepared chromosomal spreads of pollen-mother cells from WT and mutant lines. In comparison to WT, *rpa1a, rpa1c*, and *rpa1a rpa1c*, the *rpa1a* (+/−) *rpa1c* (−/−) combination displays normal meiotic progression, while the *rpa1a* (−/−) *rpa1c* (+/−) displayed both univalents and fragmented chromosomes during metaphase I (Supplementary Figure S1). In addition, *rpa1a* (−/−) *rpa1c* (+/−) combination showed highly fragmented chromosomes and unequal segregation during the subsequent stages anaphase I and II (Supplementary Figure S1). These results show that the *rpa1c* mutation in the *rpa1a* background is semi-dominant during meiotic repair, and suggest that RPA1C is either less effective at meiotic repair than is RPA1A, or that RPA1C is only required for repair of a small subset of meiotic double-strand breaks. We therefore propose that RPA1A plays the leading role in both early and late stages of meiotic crossing over, but that RPA1C can either partially or completely fulfill the role of RPA1A during early meiotic stages (DNA repair and initiation of recombination) in its absence. This is consistent with rice *rpa1a* single mutants that show defective DNA repair during meiosis (Chang et al., 2009). However, it would be useful to determine combined effects of RPA1A and RPA1C deficiency in rice for a more direct comparison of rice and Arabidopsis (monocot vs. dicot) meiotic progression, since rice *rpa1c* knock-down lines also display meiotic deficiencies (Li et al., 2013).

### The plant RPA1 gene family is composed of three distinct groups

**Figure 2:**
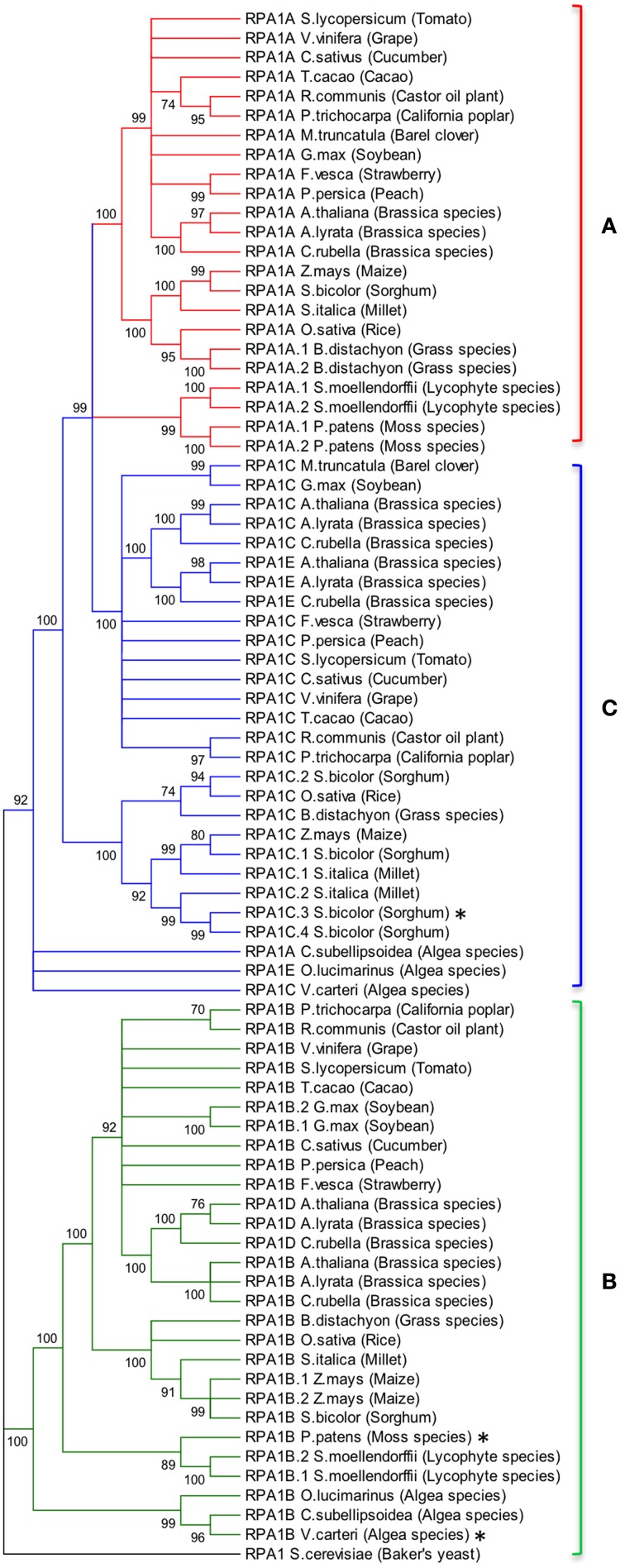
**Evolutionary relationships of RPA1 proteins**. **(A)** RPA1A group, **(B)** RPA1B group, **(C)** RPA1C group. The evolutionary history was inferred using the Maximum-Likelihood method performed with MEGA5.2 software package. Amino acid sequences were aligned using ClustalW and used to produce phylogenetic trees using the Jones-Taylor-Thornton (JTT) amino acid substitution model. Numbers next to the branches are bootstrap values (1000 replicates). Branches that show less than 70% bootstrap support were collapsed. Some RPA1 sequences denoted by asterisk (^*^) [sorghum RPA1C-3, *P. patens* RPA1B, and *V. cateri* RPA1B] contain DBD-F deletion. Yeast RPA1 was used as an outgroup to root the tree. Except for bootstrapping and choice of model, all other parameters were left at default settings.

**Figure 3:**
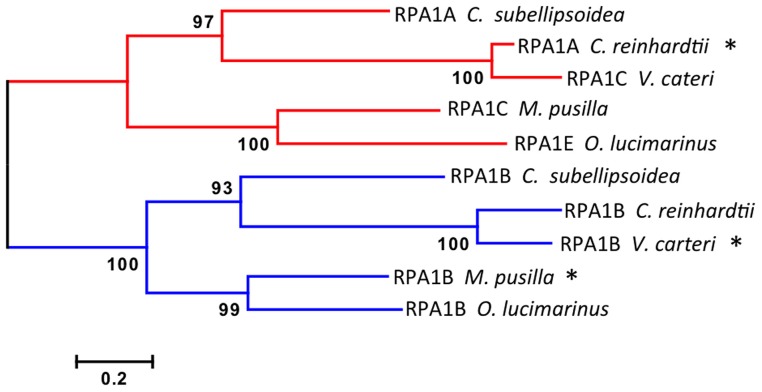
**Evolutionary relationships of unicellular green algae RPA1 proteins**. The evolutionary history was inferred using the Maximum Likelihood method performed with MEGA5.2 software package. Amino acid sequences were aligned using ClustalW and used to produce phylogenetic trees using the Jones-Taylor-Thornton (JTT) amino acid substitution model. Some RPA1 sequences denoted by asterisk (^*^) [*C. reinhardtii* RPA1A, *M. pussila* RPA1B, and *V. cateri* RPA1B] contain DBD-F deletion. Numbers next to the branches are bootstrap values (1000 replicates). Scale represents number of amino acid substitutions per site. Except for bootstrapping and choice of model, all other parameters were left at default settings.

Animal and yeast RPA1 is generally encoded by a single gene (Wold, 1997; Iftode et al., 1999). Genetic studies in rice and Arabidopsis suggest two general groups of RPA1 encoding genes, one responsible for DNA replication and one for DNA repair (Ishibashi et al., 2006; Aklilu et al., 2014). In order to determine conservation of these groups throughout the plant kingdom, we conducted BLAST searches employing known RPA1 genes in Arabidopsis and constructed maximum likelihood phylogenetic trees from identified sequences to determine phylogenetic relationships. Full-length Arabidopsis RPA1 amino acid sequences were employed for BLAST searches against the complete genome of individual plant and algae species found in the NCBI database. In addition sequences identified at each step were used to detect additional homologs. Orthology was confirmed by reverse BLAST. The lowest *e*-value and maximum percent identity sequence identified was chosen as the putative ortholog of the respective Arabidopsis RPA1 protein (Supplementary Table S1). RPA1 has four conserved domains; DBD-F, DBD-A, DBD-B, and DBD-C. Some of the putative RPA1 orthologs have either a DBD-F or a DBD-C deletion. Human RPA1 containing a DBD-F deletion retains replication activity, while a DBD-C deletion is non-functional (Gomes and Wold, 1996; Haring et al., 2008). To be as inclusive as possible while eliminating potential psuedogenes or unrelated groups, we included those putative sequences that do not contain a DBD-F domain sequence in our analysis (Figures 2, 3).

To address phylogenetic relationships of all identified sequences, we employed maximum likelihood and neighbor-joining analyses as described in the Materials and Methods section. Shown in Figure 2 is our resulting maximum-likelihood phylogenetic analysis (and is very similar employing neighbor-joining methods) of RPA1 from all identified plant-related sequences. This analysis shows the plant RPA1 family is generally divided into three distinct groups composed of RPA1A (A group), RPA1B (B group), and RPA1C (C group) (Figure 2; Supplementary Figure S2; Supplementary Table S1). RPA1D and RPA1E, which are found only in the *Brassicaceae* family, are members of the B and C group, respectively. Within each major clade (for example, RPA1B) each taxa are arranged in a sub-clade of dicot, monocot, primitive plant (*Physicomitrella patens* and *Selaginella mollendorffii*), and unicellular green algae (*V. carteri, O. lucinarinus and C. subellipsoida*). The common ancestor of RPA1B of dicots and monocots appears diverged from an RPA1B-like progenitor of primitive plants. This ancestral RPA1B in primitive plants appears to have diverged from an RPA1B-like progenitor in unicellular green algae (Figure 2, Supplementary Figure S2). The evolutionary history for RPA1A and RPA1C appears different from RPA1B. The common ancestor of RPA1A of dicots and monocots appears diverged from RPA1A of primitive plants and RPA1C of dicots. In turn, RPA1A of primitive plants and RPA1C of dicots likely diverged from the common ancestor leading to RPA1C of monocots which itself appears to have diverged from the RPA1A/C progenitor in unicellular green algae (Figure 2, Supplementary Figure S2).

Interestingly, some plants contain additional RPA1 group members. Soybean (*Glycin max*) and maize (*Zea mays*) have two RPA1B-like sequences; sorghum (*Sorghum bicolor*) and millet (*Setaria italica*) have four and two RPA1C-like sequences, respectively. Members of the *Brassicaceae* family (*A. thaliana, Arabidopsis lyrata, and Capsella rubella*) have two additional RPA1 types, RPA1D and RPA1E. In terms of both sequence and functional similarity RPA1D and RPA1E are close paralogs of RPA1B and RPA1C, respectively (Figure 2; Aklilu et al., 2014), suggesting these are duplications of RPA1B and RPA1C that occurred only in the *Brassicaceae* family. Unlike other plants, barrel clover (*Medicago truncatula*) has only two types of RPA1 (RPA1A and RPA1C) and it does not appear to have an obvious RPA1B. Given the importance of RPA1B in DNA replication (Aklilu et al., 2014) and the fact that other member of the *Fabaceae* family (Soybean) has RPA1B, it is possible that lack of this sequence is due to sequencing/annotation errors. Alternatively, barrel clover may actually lack RPA1B since the RPA1A-like sequence from barrel clover contains a subdomain that is mostly found only in the N-terminal domain of RPA1B group (see Discussion of subdomains below). If so, this might suggest that *Mt* RPA1A evolved to perform dual functions in both meiosis and replication.

From our analysis we find that primitive plant genomes generally contain two types of RPA1 sequences, RPA1A/C-like and RPA1B-like. However, only *P. patens* and *S. mollendorffii* from this group have a completed genome sequence (Goodstein et al., 2012). Additional genome sequencing of more species would be needed to determine if RPA1C is present in primitive plants.

Interestingly the RPA1A/C-like progenitor sequences of unicellular green algae are more similar to each other than to their orthologous RPA1A/C counterparts in dicots, monocots, and primitive plants (Figure 2, Supplementary Figure S2). This suggests that the plant RPA1A and RPA1C groups have evolved significantly since divergence from its counterpart in unicellular green algae, or alternatively the algae ancestor has evolved more rapidly since splitting from higher plant lineages. To determine the early evolutionary relationship of RPA1 proteins, we searched for RPA1 orthologs from two additional algal completed genomes (employing the method described above) to construct a maximum likelihood phylogenetic tree focused on algal RPA1 sequences. Interestingly, all five unicellular green algae that represent five different genera have two types of RPA1 sequences, an RPA1B-like group common to all the algae representatives in this study, and an RPA1A/C-like group (Figure 3; Supplementary Table S1; Supplementary Figure S2). This suggests that the first duplication event of RPA1 that led to diversification in plants likely occurred during the evolution of green algae. This is further supported by the fact that red algae generally contain only a single RPA1-like sequence (Supplementary Table S2). We propose a model (described below) for the expansion of RPA1 from green algae to higher plants based upon the phylogenetic relationships and sequence groups described above, in combination with RPA1 domain structure described in the next five sections below.

### Structure and domains of plant RPA1 proteins

**Figure 4:**
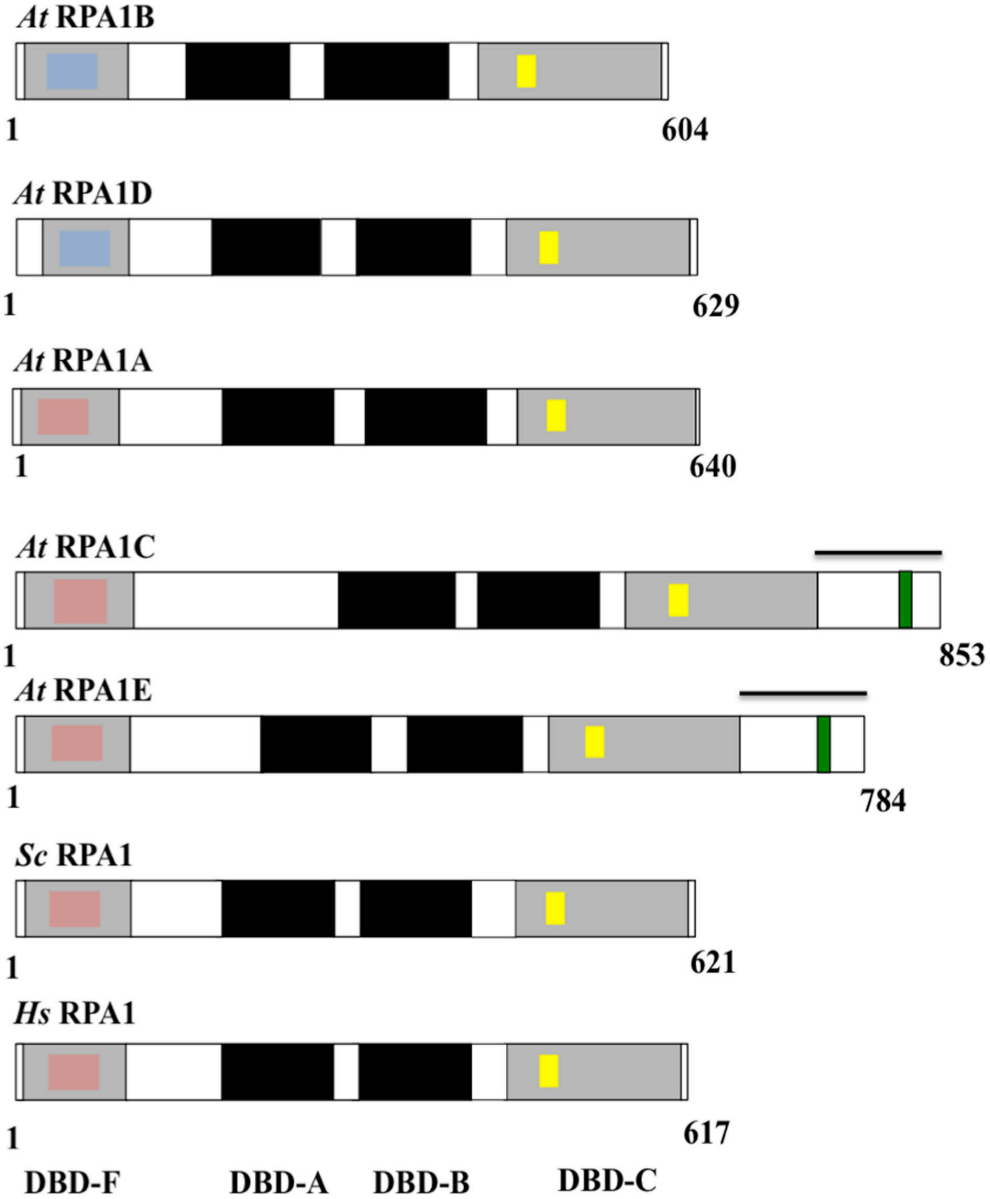
**Schematic diagram of the structure and functional domains of RPA1 proteins**. *At, Arabidopsis thaliana; Sc, Saccharomyces cerevisiae; Hs, Homo sapiens*. Blue inset boxes represent Generic Binding Surface I. Pink inset boxes represent Binding Surface I (Basic Cleft). Yellow inset boxes represent C5 (in *At* RPA1B, D), C6 (in *At* RPA1A, C, E), and C4 (in *Sc* RPA1and *Hs* RPA1)—type zinc-finger motifs. Green inset boxes represent CCHC-type zinc-finger motif. Bars above *At* RPA1C and *At* RPA1E indicate C-terminal extension region.

Eukaryotic RPA1 contains four highly-conserved domains termed the N-terminal domain or DNA Binding Domain F (DBD-F), two structurally similar central DNA Binding Domains (DBD-A and DBD-B), and a C-terminal DNA Binding Domain C (DBD-C) (Wold, 1997; Takashi et al., 2009; Figure 4). Studies in yeasts and animals suggest that the N-terminal domain (DBD-F) is primarily involved in protein-protein interactions, the middle domains (DBD-A and DBD-B) function in ssDNA binding, while the C-terminal domain (DBD-C) is involved in DNA damage recognition during nucleotide excision repair and subunit interaction (Wold, 1997; Lao et al., 2000; Zou et al., 2006).

To characterize conservation of these domains throughout plant RPA1 sequences as well as relate known functional characteristics to domain structure, we compared all *A. thaliana* RPA1 sequence domain structure to human and yeast RPA1as summarized in Figure 4. We employed the NCBI Conserved Domain Database [CDD] (Marchler-Bauer et al., 2011) to identify RPA1 structure, domains, and sub-domains. All sequences contain these four conserved domains (DBD-F, DBD-A, DBD-B, DBD-C). However, we find that each group contains unique subdomains or motifs that may contribute to functional differences. In the following sections we describe these subdomains and motifs in detail. With very few exceptions (Sorghum RPA1C and *P. patens* RPA1B contain DBD-F deletion), the general structure, domains, subdomains, and motifs of the three groups of *A. thaliana* RPA1 proteins are conserved throughout all plants examined here (Supplementary Figures S3–S6).

### RPA1B is unique among other RPA1 sequences through loss of the binding surface I domain

DBD-F of human RPA1 contains a subdomain called “Binding Surface I” (BS-I) or basic cleft (Figure 4, pink inset box) (Jacobs et al., 1999; Marchler-Bauer et al., 2011). Multiple studies demonstrate that the BS-I subdomain in human RPA1 and a similar region in yeast RPA1 is required for DNA repair activity but not required to support DNA replication (Longhese et al., 1994; Umezu et al., 1998; Bochkareva et al., 2005; Haring et al., 2008; Xu et al., 2008). Five highly conserved amino acid residues constitute the protein interaction sites of BS-I; position 1 is generally a polar residue (N/Q/S/T), position 2, 3, and 5 are basic (R/K), and position 4 is hydrophobic (L/V/I) (Supplementary Figure S3A; Supplementary Table S3). In all plants analyzed here, except Sorghum RPA1C-3, sequences phylogenetically predicted to fall into the RPA1A and RPA1C groups generally display these same conserved features as found in animal and fungal RPA1 (Figure 4; Supplementary Figures S3B,C). One notable exception among plant sequences is at position 1 where the conserved polar residue (N/Q/S/T) is replaced by a negatively charged residue (D/E). Interestingly, the predicted plant RPA1B group members do not display the conserved features of BS-I in most or all five positions, being replaced by chemically non-similar residues (Supplementary Figure S3D). For instance, RPA1B sequences generally contain a negatively charged amino acid (D) at position 1 instead of a conserved polar residue. In addition, position 3 in most RPA1B sequences contains a polar residue, while position 5 contains a negatively charged residue (E/D), instead of the conserved basic residues (R/K). Similarly, the hydrophobic residue found at position 4 (L/V/I) is in most cases replaced by a polar residue (T/S). These data suggest that plant RPA1B evolved into a more specialized member of the RPA1 family through loss of this domain (BS-I) to interact primarily with DNA replication-oriented pathways consistent with proposed functions of this group (Aklilu et al., 2014). In unicellular green algae only two of the five RPA1A/C progenitor sequences (*O. lucimarinus* RPA1E and *M. pussila* RPA1C) contain BS-I. The other sequences have either an N-terminal (DBD-F) deletion (*C. reinhardtii* RPA1C) and thus do not contain BS-I, or have N-terminal domain but do not contain BS-I (*V. cateri* RPA1C and *C. subellipsoida* RPA1A).

### RPA1B sequences contain a conserved N-terminal (DBD-F) nucleic acid binding surface termed generic binding surface I

OB-fold domains are known for their ssDNA-binding activity. However, no clear conservation of amino acid residues that directly interact with nucleic acids has been identified among the domains (Theobald et al., 2003). For example, except for two aromatic residues that stack with DNA bases, there is no conservation among the rest of the 6–9 amino acids that are predicted to contact ssDNA and are found in the two main ssDNA-binding OB fold domains (DBD-A and DBD-B) of human RPA1 (Bochkarev et al., 1997). Nevertheless, alignments of multiple OB fold domains reveal a pattern of hydrophobic residues, mostly flanked by polar or charged residues, in alternating amino acid positions that are conserved for short stretches of sequence around the site of ssDNA-binding (Theobald et al., 2003). These patterns are generally referred to as Generic Binding Surface I (GBS-I), and are found in DBD-A, DBD-B, and DBD-C of eukaryotic RPA1.

Analysis of plant RPA1 sequences employing NCBI CDD reveals that an additional GBS-I domain is present in DBD-F of RPA1B group sequences [Figure 4 (blue inset box); Supplementary Figure S4A]. This domain displays the classic pattern of hydrophobic residues flanked by polar or charged residues found in all other GBS-I domains, although the conservation of amino acid identity is unique among this particular domain in plant DBD-F. Interestingly, some plant RPA1A- and RPA1C-like sequences also contain this GBS-I in DBD-F (Supplementary Figure S4C) and these special cases are discussed below. In unicellular green algae only three of the five RPA1B-like sequences (RPA1B of *O. lucimarinus, C. reinhardtii*, and *C. subellipsoida*) contain GBS-I. The other two sequences (RPA1B of *M. pussila* and *V. cateri*) do not contain GBS-I as they have a truncated N-terminal domain (DBD-F). Interestingly, however, RPA1A of *C. subellipsoida* does contain a GBS-I domain in DBD-F. In general, this domain structure is unique to plant and green algae, and not found in animals and fungi.

Human RPA binds to ssDNA in two modes. The first mode has an occluded size of 8–10 nucleotides (Blackwell and Borowiec, 1994) and accomplished by the two major ssDNA-binding domains, DBD-A and DBD-B (Bochkareva et al., 2001; Hass et al., 2012). The second binding mode has an occluded binding size of ~30 nucleotides (Kim et al., 1992, 1994) and involves DBD-A, -B, and -C of RPA1 and DBD-D of RPA2 (Bastin-Shanower and Brill, 2001; Cai et al., 2007). DBD-F has a weak ssDNA-binding affinity (Daughdrill et al., 2001; Bochkareva et al., 2005) and is not involved in either binding modes (Cai et al., 2007). The ability of RPA binding to short nucleotides (8–10 nt) is required for DNA repair function, but not DNA replication (Hass et al., 2012). Conversely, it is proposed that the ability of RPA to bind to long stretches of ssDNA (~30 nt) is required for its function in DNA replication rather than in DNA repair, as there is extensive DNA unwinding and exposure of long ssDNA intermediates during DNA replication vs. DNA repair (Hass et al., 2012). Accordingly, our result suggests that in plants, DBD-F of the RPA1B group may participate in ssDNA-binding (as it contains GBS-I) so that together with other DBDs (DBD-A, DBD-B, DBD-C, and DBD-D in RPA2) it may result in an occluded size of more than 30 nucleotides.

The presence of GBS I in DBD-F of barrel clover and *Brachypodium distachyon* RPA1A-like sequences, and millet and sorghum RPA1C-like sequences (Supplementary Figure S4C) suggests these sequences could participate in DNA replication activities. For example, since we were unable to identify a predicted RPA1B sequence in barrel clover, it is possible the RPA1A-like sequence might have gained the GBS I subdomain to function in place of RPA1B. *B. distachyon* has two RPA1A (RPA1A-1 and RPA1A-2), perhaps allowing the paralogs to accumulate small gradual mutations and thereby gain new domains and functions as purifying selection is weaker on duplicate genes (Castillo-Davis et al., 2004). This same explanation can be argued for RPA1C-2 and RPA1C-4 of millet and sorghum, respectively.

### Plant RPA1 sequences contain unique zinc-finger motifs within the DBD-C C-terminal domain

RPA1 sequences from yeasts and animals contain a C4-type (C-X_2_-C-X_13_-C-X_2_-C) zinc-finger motif (ZFM) within the DBD-C domain (Wold, 1997). This motif is required for DNA replication (Lin et al., 1996, 1998; Walther et al., 1999), DNA damage recognition (Lao et al., 2000), and for proper structural formation of RPA complexes (Lin et al., 1996). Employing NCBI CDD protein domain analysis we find that indeed all plant RPA1 sequences also contain ZFM sequences. However, RPA1A and RPA1C contain a C6-type (C-X_3_-C-X_8_-C-X_13_-C-X_2_-C-X_6_-C) ZFM, while RPA1B contains a C5-type (C-X_2_-C-X_13_-C-X_2_-C-X_6_-C) ZFM. Conservation of these domains among all plant species analyzed is high, with only a few exceptions seen in sorghum RPA1C-3 and RPAC-4, rice RPA1C, and in the Lycophyte *S. moellendorffii* RPA1B-like sequences (Supplementary Figure S5). RPA1A/C-like progenitor sequences of unicellular green algae contain a C-4 type ZFM (Supplementary Figure S5E). However, in *M. pussila* RPA1C and *O. lucimarinus* RPA1E, the fourth residue of the conserved motif is histidine (H) instead of cysteine (C). Interestingly, RPA1B-like sequences of unicellular green algae do not contain a conserved C4-type ZFM in DBD-C (Supplementary Figure S5F).

ZFMs are known for their role in protein-DNA and protein-protein interactions (Krishna et al., 2003; Gamsjaeger et al., 2007). The human RPA1 ZFM interacts with both normal and damaged ssDNA with low and high affinity, respectively, thereby contributing to optimal ssDNA-binding activity (Dong et al., 1999; Walther et al., 1999; Lao et al., 2000). Human RPA1 that contains a mutation in either the two cysteine amino acids of the ZFM, or a complete deletion of the motif, retains ssDNA binding activity, heterotrimeric complex formation, and DNA repair promoting activities, but does not support DNA replication (Lin et al., 1996, 1998; Walther et al., 1999). The latter replication defect may be due to a failure to load polymerase δ at replication forks during the elongation step of DNA replication (Lin et al., 1998), or due to improper heterotrimeric complex formation, as the ZFM generally affects the structure of the RPA complex (Dong et al., 1999; Walther et al., 1999; Bochkareva et al., 2000). In plants, it is possible that the different types of ZFMs in the RPA1A/1C and RPA1B group may contribute to the functional specificity of these proteins in DNA metabolism through direct and specific protein-DNA and/or protein-protein interactions. The ZFMs may also contribute to the formation of different types of RPA heterotrimeric complexes. In support of this, Arabidopsis RPA1A preferentially forms a complex with RPA2B, and RPA1B preferentially forms a complex with RPA2A (Eschbach and Kobbe, 2014). Also in rice, each of the three RPA1 subunits preferentially forms a complex with a specific RPA2 subunit (Ishibashi et al., 2006).

### RPA1C group has a C-terminal extension region that contains a glycine/serine-rich domain interspersed by a CCHC-Type ZFM

In contrast to other RPA1 sequences examined here, plant RPA1C-like sequences (including RPA1E in *Brassicaceae*) contain a unique C-terminal extension region that contains an average amino acid sequence length of ~176 in RPA1C and ~119 in RPA1E-like sequences (Figure 4; Supplementary Table S4). CLUSTAL alignments of these C-terminal extensions (beginning at the end of the putative DBD-C domain in each case to the end of the predicted sequence), NCBI CDD domain searches, and manual comparisons were employed to identify common sequence domains or regions. Common among all of these sequences is the presence of at least one zinc-knuckle CCHC-type (CX_2_CX_4_HX_4_C) ZFM. All *Brassicaceae* members display one ZFM in RPA1C, and one ZFM in RPA1E C-terminal extensions, are roughly at the same position within the alignments (both C-terminal only, and the full-length sequence alignments), and share high sequence identity. Interestingly, in plants that do not have the RPA1C/RPA1E paradigm (non-*Brassicaceae*), the RPA1C sequence(s) contain multiple ZFM that fall into two clusters (termed here C1 and C2) each with unique sequence identity (Supplementary Figure S6). In most cases (tomato, cucumber, and maize for example) there are two unique ZFM separated by ~30 amino acids (C1 followed by C2). In other cases, such as rice for example, there is a C1 followed by multiple (three in this case) C2s. The fact that there are two unique cluster types of the ZFM suggests either (1) that these motifs arose independently through acquisition of ZFM-like sequences from two independent sources (genes) in the ancestral gene, or (2) were duplicated from the same ancestral gene but evolved early into two unique ZFM sequences. In the case of *Brassicaceae*, a likely scenario is that the ancestral RPA1C that gave rise to current RPA1C and RPA1E contained both C1 and C2, but in each case lost C2. If so, this would suggest C2 is only necessary in the presence of C1 to carry out multiple DNA repair-related functions. In unicellular green algae only one of the RPA1A/C progenitor sequences (*M. pussila* RPA1C) contains CCHC-type ZFM in the C-terminal extension region. The other sequences either do not contain a C-terminal extension region (*O. lucimarinus* RPA1E) or contain a C-terminal extension region that does not have CCHC-ZFM (*V. cateri* RPA1C).

This analysis also revealed that the C-terminal extension regions in RPA1C sequences are glycine- and serine-rich (Supplementary Figures S6A,B). Glycine-rich regions are found in RNA-Binding Proteins (RBPs) and they may be enriched by additional polar (hydrophilic) residues, such as serine, arginine, asparagine, glutamine, and tyrosine. However, the function of these additional polar residues is poorly understood (Rogelj et al., 2011). Glycine-rich domains interspersed by CCHC-type ZFMs are found in RNA-binding plant proteins involved in post-transcriptional regulation of gene expression under various stress conditions (Karlson et al., 2002; Karlson and Imai, 2003; Kim et al., 2005, 2007; Kim and Kang, 2006). Therefore, it is possible that RPA1C may also bind to RNA and play a role in post-transcriptional regulation. Furthermore, CCHC-type ZFMs are predicted to bind to both normal GT-rich ssDNA (Rajavashisth et al., 1989; Tzfati et al., 1995; Kim et al., 2005) and damaged ssDNA [e.g., Arabidopsis DDB2 (Ly et al., 2013), yeast RAD18 (Jones et al., 1988), and human PARP-1 (Langelier et al., 2011)]. This suggests that besides binding to RNA and ssDNA and thereby playing regulatory role, the CCHC-type zinc-finger motif may also play a role in DNA damage recognition, as suggested from genetic analysis (Aklilu et al., 2014).

**Figure 5:**
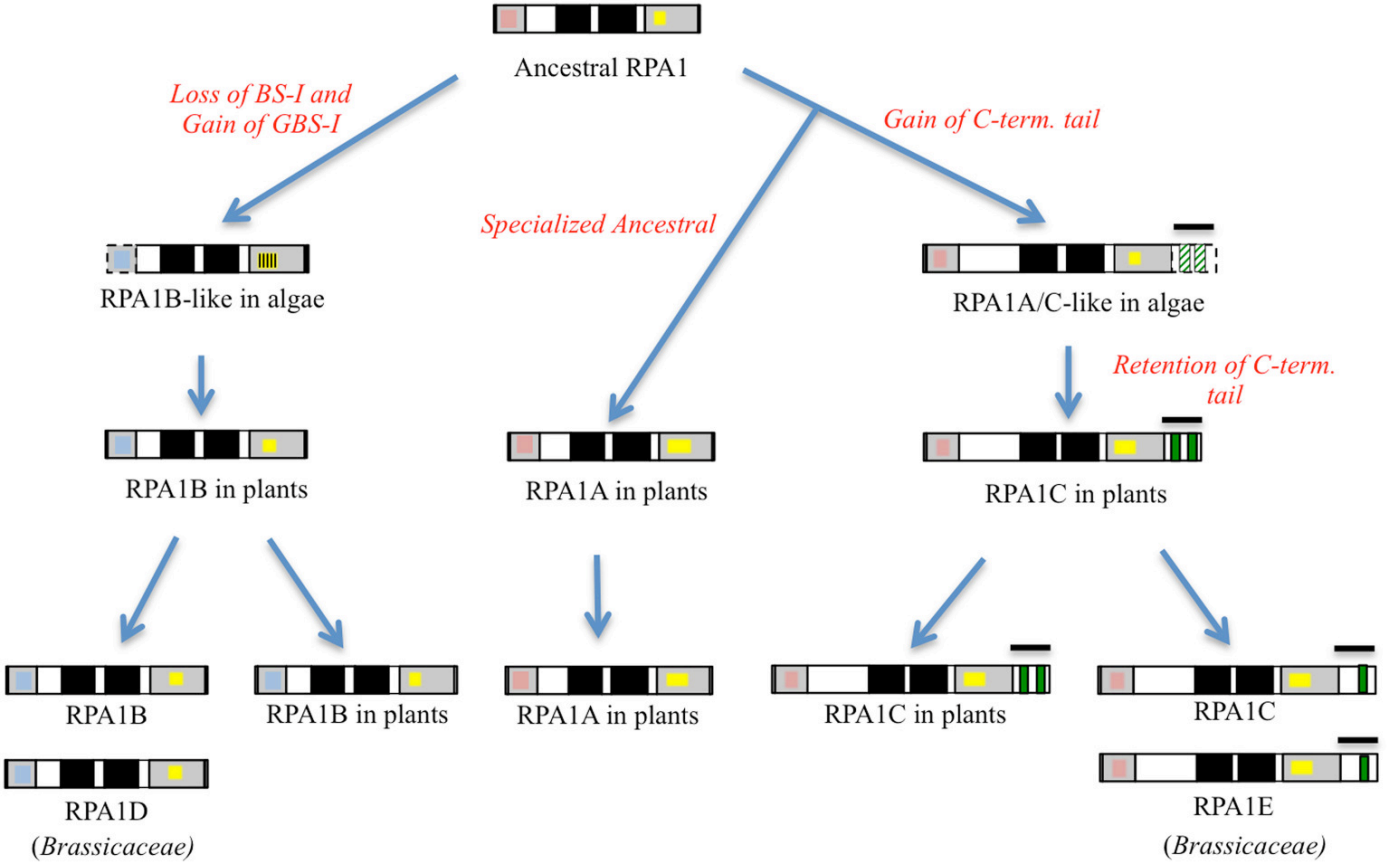
**Proposed model of RPA1 evolution in plants and algae**. Gray boxes from right to left are DBD-F and DBD-C, respectively, dark boxes from right to left are DBD-A and DBD- B, respectively, red boxes are Binding Surface I (BS-I), blue boxes are Generic Binding Surface I (GBS-I), yellow boxes are C4/C5/C6—type zinc-finger motifs (ZFM), green boxes are CCHC-type ZFM. Line bars indicate C-terminal extension (tail) region, broken lines and strips indicate poorly conserved domains.

In summary, the protein structure analysis result is consistent with genetic and phylogenetic data that plant RPA1 proteins fall into three distinct groups with unique functions (RPA1A, RPA1B, and RPA1C). Based on these data we propose that duplication of RPA1 in unicellular green algae led to two main progenitor groups in primitive plants, and later diverged into three groups in higher plants with specialized functions (Figure 5).

### The *RPA1B* group has higher levels of gene expression, intron frequency, and optimal codon usage

**Figure 6:**
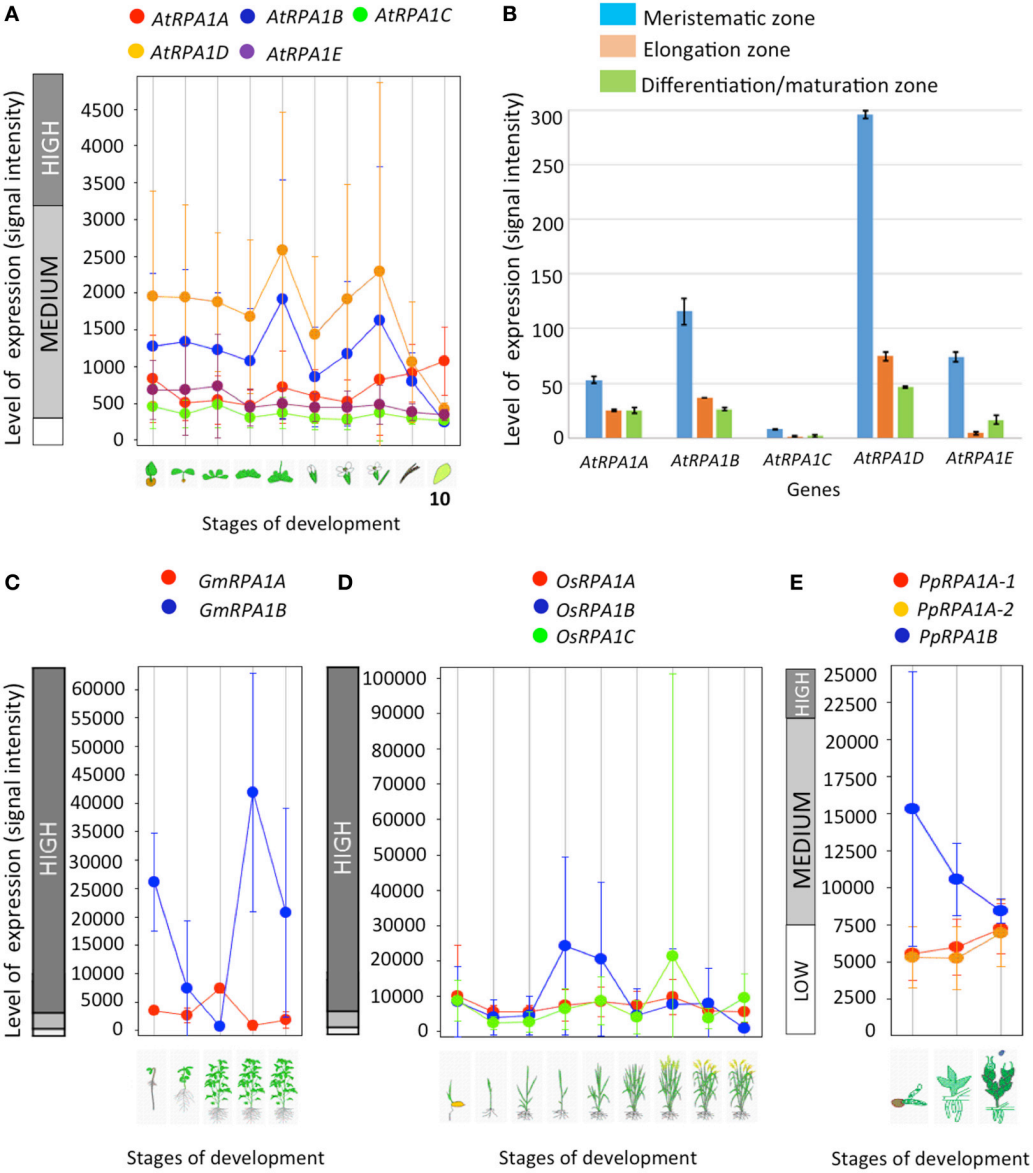
**Level and pattern of ***RPA1*** expression at different developmental stages and root zones. (A)** *A. thaliana*; (1) Germinated seed, (2) Seedling, (3) Young rosette leaf, (4) Developing rosette leaf, (5) Bolting, (6) Young flower, (7) Developed flower, (8) Flowers and siliques, (9) Mature siliques, (10) Senescence. **(B)** *AtRPA1* expression in different root zones of 7-day-old Arabidopsis seedlings. **(C)** Soybean; (1) Germination, (2) Main shoot growth, (3) Flowering, (4) Fruit formation, (5) Bean development. **(D)** Rice; (1) Germination, (2) Seedling, (3) Tillering stage, (4) Stem elongation stage, (5) Booting stage, (6) Heading stage, (7) Flowering stage, (8) Milk stage, (9) Dough stage. **(E)** Psychomitrela patens; (1) Germination [protenema development], (2) Gametophore growth, (3) Gametangia development. Data were collected from genevestigator (**A,C–E**; Hruz et al., 2008) and Arabidopsis eFP Browser (**B**; Winter et al., 2007). Error bars indicate standard error.

Depending on their functional specificity and biochemical role, genes in general and duplicated genes in particular can have different temporal, spatial, and levels of gene expression (Chiapello et al., 1998; Casneuf et al., 2006; Hyun et al., 2014). As discussed above, genetic analysis of *rpa1* mutants suggests specialized functions for individual RPA1 subunits. Based on this we predict unique gene expression patterns of RPA1 genes that are consistent with their proposed function. For example, we might expect to see higher basal expression of individual RPA1 members if they participate in “housekeeping” type functions, such as normal DNA replication activity. To this end, we examined expression patterns and levels of the Arabidopsis, soybean, rice, and *P. patens* RPA1 paralogs (Figure 6) from Genevestigator, an online gene expression visualization tool that summarizes results from thousands of high quality transcriptomic experiments, typically employing cDNA microarrays (Hruz et al., 2008). In general the *RPA1B* group displays higher basal expression levels in most developmental stages in comparison with the *RPA1A* and *RPA1C* groups of all four included plant species, in both shoots and roots (Figures 6A,B). In contrast, members of the *RPA1C* group and to a lesser extent the *RPA1A* group are induced by DNA damage (Culligan et al., 2006). The higher overall level of gene expression of the RPA1B group suggests a requirement throughout the developmental growth of the plant, and is consistent with the proposed function of RPA1B in normal DNA replication functions (Aklilu et al., 2014).

**Figure 7:**
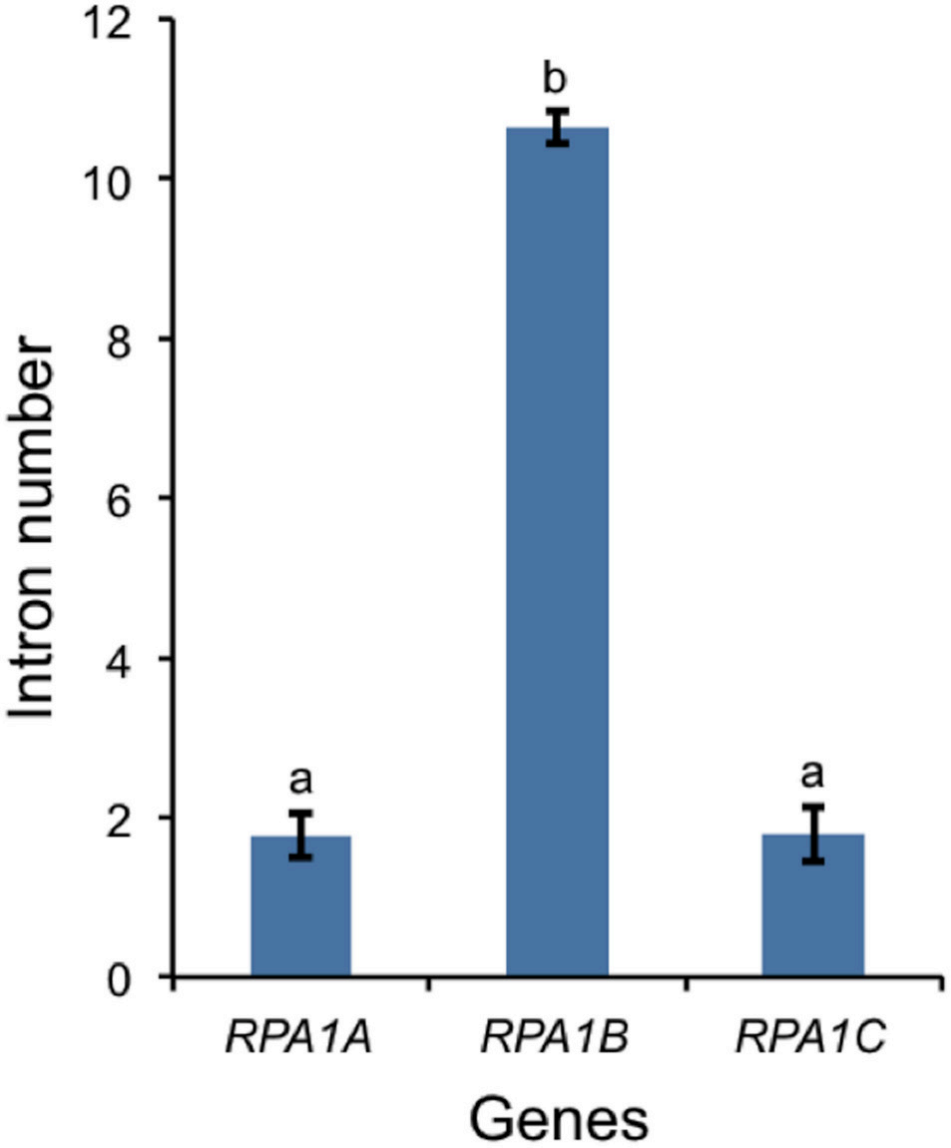
**Number of introns in plant RPA1 genes**. Twenty plants (Supplementary Table S1) are included in the analysis. Data are mean ± SE. To analyze statistical difference *F*-test (ANOVA) and LSD were carried out at *P* ≤ 0.05. Bars with different letters indicate significant differences.

In *A. thaliana, RPA1B* and *RPA1D* display ~10-fold more introns vs. *RPA1A, RPA1C*, and *RPA1E* (Supplementary Table S1). To determine if this paradigm is consistent throughout plants, we established intron frequencies in all three major groups of *RPA1* paralogs (*A, B*, and *C*) as predicted by our phylogenetic analysis above, employing NCBI sequence databases. As shown in Figure 7, *RPA1B*-like genes from 20 plant species contain ~six-fold more introns on average than either group *RPA1A*, or group *RPA1C*. However, in unicellular green algae, there is no clear pattern of intron frequency between the B-like group and A/C-like group (Supplementary Table S1). In many eukaryotes, intron frequency is generally proportional to gene expression levels (Callis et al., 1987; Brinster et al., 1988; Duncker et al., 1997; Juneau et al., 2006; Shabalina et al., 2010), and mRNA stability (Le Hir et al., 2003; Niu and Yang, 2011). For example, highly expressed genes in both Arabidopsis and rice show increased intron frequency (number of introns per kilobase of coding sequence) vs. lower expressed genes (Ren et al., 2006). Therefore, these data are consistent with higher gene expression levels of *RPA1B* group members vs. *RPA1A* or *RPA1C* members.

**Figure 8:**
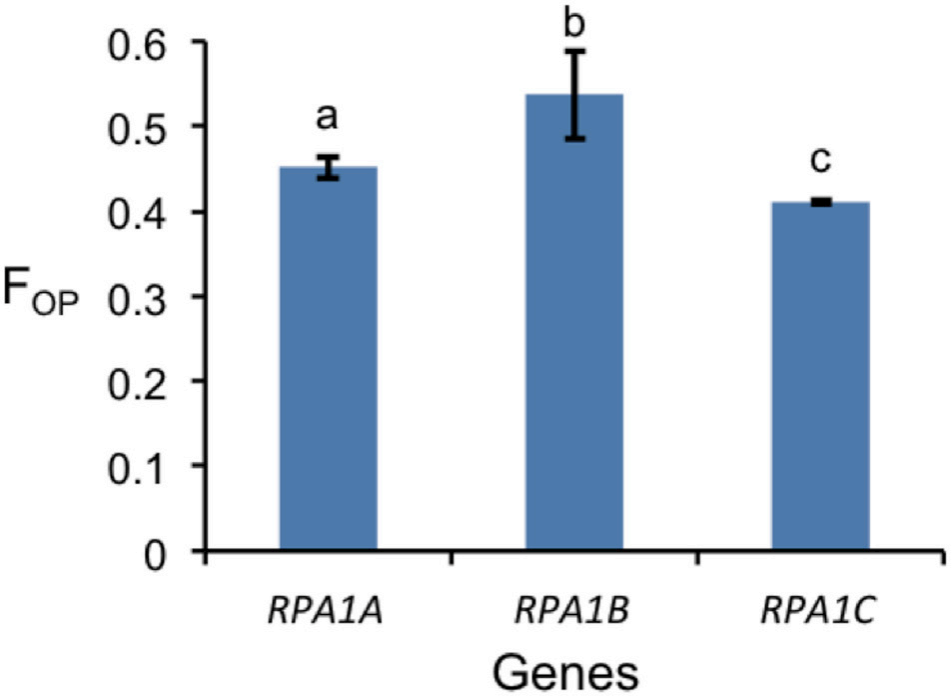
**Frequency of optimal codon (F_OP_) values for RPA1 genes of Arabidopsis and rice**. Data are mean ± SE. To analyze statistical difference *F*-test (ANOVA) and LSD were carried out at *P* ≤ 0.05. Bars with different letters indicate significant differences.

Codon preference (optimal codons) is determined by tRNA abundance and gene expression level and occurs with unequal frequencies (Ikemura, 1985; Duret and Mouchiroud, 1999). Highly expressed genes in both prokaryotes and eukaryotes have increased frequency of codons that match abundant tRNAs (Ikemura, 1985; Bulmer, 1988; Wright et al., 2004). Codon bias may reflect a selective pressure to enhance translational efficiency for highly expressed genes (Bulmer, 1988; Marais and Duret, 2001; Wright et al., 2004; Sablok et al., 2013). Since the *RPA1B* group displays higher overall gene expression levels vs. *RPA1A* and *RPA1C*, we further hypothesized that RPA1 genes may reflect this through a bias in optimal codon usage. In order to test this we calculated the frequency of optimal codons (F_OP_) for Arabidopsis and rice RPA1 genes, since these species have the most complete data set of developmental stages from Genevestigator (Hruz et al., 2008) and optimal codons for highly expressed genes have been identified in these organisms (Liu et al., 2004; Wright et al., 2004). F_OP_ is calculated as the number of occurrences of optimal codons divided by the total number of codons (Ikemura, 1985). As shown in Figure 8, we find that the *RPA1B* group has the highest number of optimal codons (F_OP_ = 0.54) followed by *RPA1A* (F_OP_ = 0.45) and *RPA1C* (F_OP_ = 0.41). F_OP_-values for the Arabidopsis *RPA1D* (0.45) and *RPA1E* (0.42) are not included in the *F*-test as they are only found in *Brassicacea*. This suggests that *RPA1B* group is likely under the control of translational selection due to a demand for its higher translational protein product during DNA replication.

### *RPA1A* and *RPA1B* are more conserved than *RPA1C*

Highly expressed genes are usually under a high degree of selective constraint and thus display a higher degree of conservation (Pál et al., 2001; Drummond et al., 2005). As described above, plant RPA1 groups have unique expression patterns (Figure 6). Accordingly, we hypothesize unique sequence conservation of RPA1 subunits that is consistent with their expression level. To test this, we analyzed and compared the type and degree of natural selection applied on each *RPA1* group. Natural selection is measured by the ratio of non-synonymous (*dN*) and synonymous (*dS*) nucleotide substitution rates (ω or *dN*/*dS*) and its value ranges from zero (0) to infinity (∞). While a value of < 1 is a sign of purifying selection (where sequence conservation is preferred), a value of >1 is a sign of positive selection (where change in sequence is preferred). We carried out the analysis by dividing plants into two groups. (1) The *Brassicacea* group (*A. thaliana* and *A. lyrata*), *RPA1* from this group is analyzed separately because it has five paralogs, and (2) Other dicot group that contains only three *RPA1* paralogs (Tomato, Cucumber, Strawberry, Castor oil plant, Grape, Cacao, Peach, and California Poplar). Plants that have only two RPA1 paralogs or more than three, as a result of lineage specific duplication, were not included in the analysis since these cases would affect sequence conservation and selection, and comparison of orthologs would lead to unreliable comparisons. *dN* and *dS* analyses employed *RPA1* coding sequence comparisons to a common “outgroup” orthologous *RPA1* (pair-wise comparison). To this end, *RPA1* of *C. rubella* and rice were used as an “outgroup” to the first and second group, respectively.

**Figure 9:**
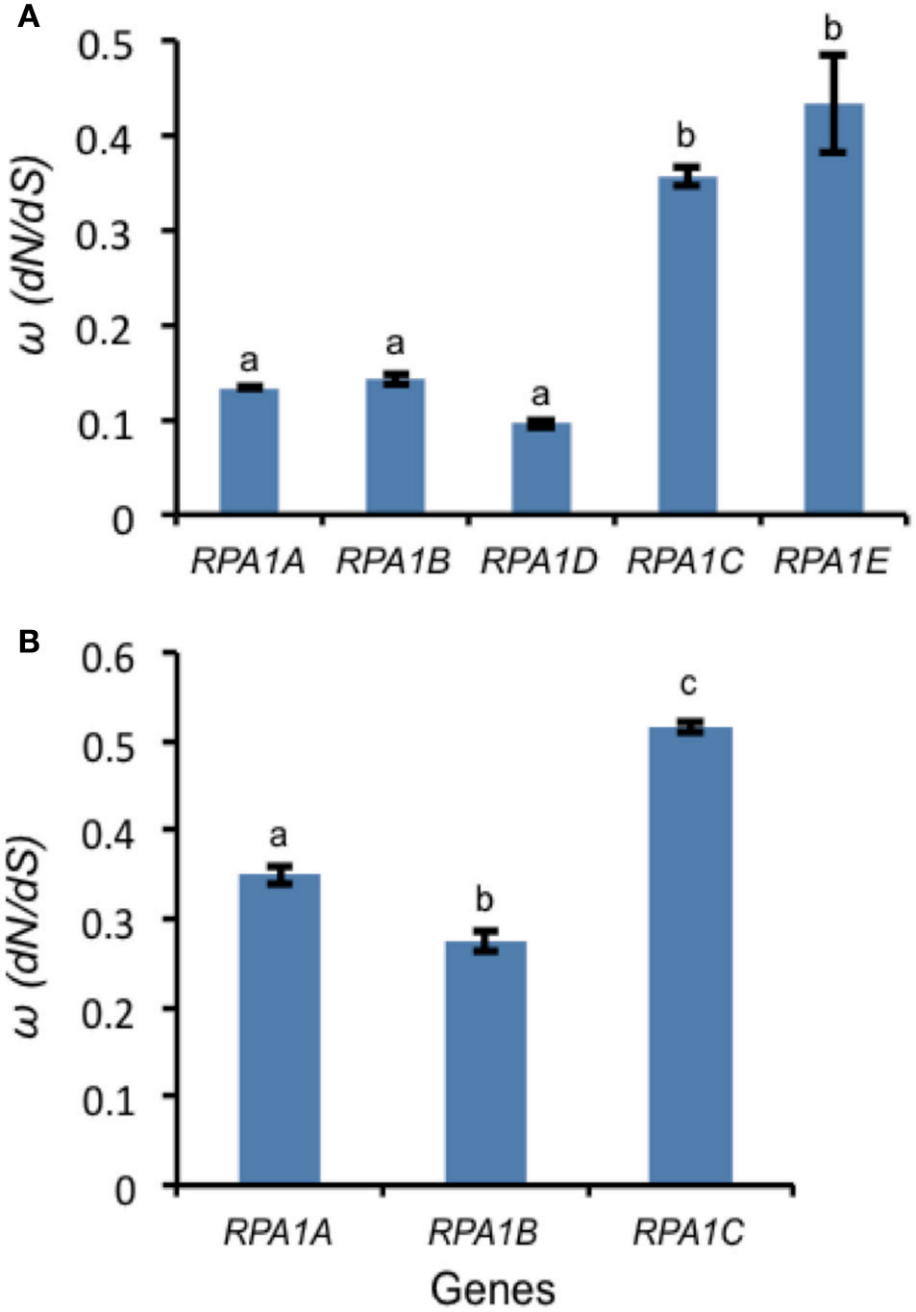
**Natural selection (ω) values for ***RPA1*** genes. (A)** ω-values for Arabidopsis and *A. lyrata RPA1* genes. *C. rubella RPA1* genes were used as a reference for ortholog pairwise sequence distance measurement (*dN* and *dS*). **(B)** ω-values for RPA1 genes of eight plants (Tomato, Cucumber, Strawberry, Castor oil plant, Grape, Cacao, Peach, and California Poplar). Rice *RPA1* genes were used as a reference for ortholog pairwise sequence distance measurement (*dN* and *dS*). *dN* and *dS* analyses were conducted in MEGA5 using the Nei-Gojobori model. Data are mean ± SE. To analyze statistical difference *F*-test (ANOVA) and LSD were carried out at *P* ≤ 0.05. Bars with different letters indicate significant differences.

All *RPA1* are under purifying selection, as they all have ω-values < 1 (Figure 9). However, the degree of selection pressure or conservation is stronger on *RPA1B* followed by RPA1A and *RPA1C*. This can be due to the difference in gene expression level and consistent with the high transcript accumulation of *RPA1B* group vs. the *RPA1A/C* group (Figure 6). However, gene expression alone does not explain the sequence conservation difference seen among the groups as, for example, there is no clear expression difference between the RPA1A group and RPA1C group (Figure 6). In this case, functional differences may play a role. While RPA1A is primarily responsible for the highly conserved meiotic DNA recombination process, RPA1C likely functions in many types of DNA repair pathways and interacts with various proteins found in each pathway. Proteins involved in many biochemical pathways that employ various interaction partners tend to be more conserved (Krylov et al., 2003). However, since some repair pathways and associated proteins are relatively less conserved across plants (Singh et al., 2010), lineage specific co-evolution of RPA1C may result in less conservation of the protein across species.

### *Arabidopsis RPA1* paralogs contain unique *cis*-acting element composition associated with biological function

As discussed above, *RPA1* members display unique gene expression patterns consistent with their proposed function in meiosis, DNA replication and repair. Accordingly, we hypothesize that respective *RPA1* paralog members should display *cis*-acting elements that regulate their spatial, temporal and induced expression pattern consistent with proposed activities. To identify *cis*-acting regulatory elements, sequences were analyzed using PLACE (Plant *cis*-acting regulatory DNA elements) database (Higo et al., 1999). For this analysis we used two sets of promoter sequences. The first set includes predicted promoter sequences upstream of transcriptional start site for each paralogs retrieved from TAIR (The Arabidopsis Information Resource) database. Since the predicted promoter sequence of each paralog is different in length, we also analyzed a second set with an equal length (1794 bp) of promoter sequence for each paralog (Table 1). This is based on the predicted promoter sequence length of *RPA1A* that contains the largest promoter region of the group. A complete list of *cis*-acting elements along with their description is presented in Supplementary Table S5.

**Table 1:** *****Cis***-elements in Arabidopsis ***RPA1*** promoters^a^**.

**Category of *cis*-elements**	**Number of *cis*-elements in Arabidopsis *RPA1* promoters**				
	***RPA1A***	***RPA1B***	***RPA1D***	***RPA1C***	***RPA1E***
Reproductive phase transition and flower related	16	0	4	1	1
	**16**	3	4	12	3
Pollen related	**21**	7	13	8	7
	21	25	27	21	28
Cell cycle and DNA synthesis related	0	3	1	0	0
	0	**5**	**3**	0	0
Senescence related	5	0	0	0	3
	**5**	0	0	0	3
Seed development and germination related	33	1	28	25	10
	33	34	38	**47**	**46**
Abiotic stress related	43	1	20	24	16
	43	27	42	**57**	**75**
Biotic stress related	16	8	24	7	11
	16	30	**36**	25	29

a *Cis-acting regulatory DNA elements in the RPA1 promoter sequences. Numbers indicate the total number of cis-elements in the respective category. Bold numbers indicate the highest number of cis-elements in each category. Each category has two rows filled with numbers. The numbers in the upper row are based on promoter sequences of varying length (RPA1A = 1794 bp, RPA1C = 873 bp, RPA1E = 571 bp, RPA1B = 372 bp, RPA1D = 1174 bp) as obtained from TAIR data base. The numbers in the lower row are based on optimized promoter sequence length (equal length, 1794 bp, for each RPA1)*.

As is summarized in Table 1, *RPA1A* promoter sequences are enriched in *cis*- elements related to reproductive phase transition, flower, and senescence vs. other RPA1B and RPA1C group members. In addition, these sequences appear enriched in pollen-related *cis*-acting elements vs. other groups, but only when limited to predicted promoter sequences (Table 1). These data are consistent with a proposed leading role for *RPA1A* during meiosis, and coincides with *RPA1A* expression up-regulation immediately after reproductive phase transition, and during flowering and pollen formation stage (Figure 6A). Interestingly, *RPA1A* promoter enrichment of *cis*- acting elements related to senescence is well correlated to its late-developmental stage (senescence) transcriptional up-regulation (Figure 6A), suggesting a potential role for *RPA1A* in regulation of senescence, but this would need to be tested biologically.

*Cis*-elements related to DNA synthesis and cell cycle regulation were only identified in the promoters of *RPA1B* and *RPA1D*, consistent with their proposed primary role in DNA replication (Aklilu et al., 2014). Interestingly, *RPA1D* appears to be uniquely enriched in *cis*-elements related to biotic stress responses.

Lastly, both *RPA1C* and *RPA1E* display enrichment of *cis*-elements related to seed development and germination and abiotic stress (Table 1). Obviously, abiotic stress encompasses a wide spectrum of insults to tissues (Waterworth et al., 2011; Roy, 2014), but includes agents that damage DNA, such as UV light or ionizing radiation for example. *RPA1C* and *RPA1E* are predicted to play a leading role in DNA repair activities (Aklilu et al., 2014) and are induced by ionizing radiation (Culligan et al., 2006), suggesting perhaps many of these abiotic stress-related elements are directly involved in the transcriptional response to DNA damage. Interestingly, DNA repair pathways are known to regulate seed quality and longevity by repairing DNA damage accumulated during storage and imbibition (Cheah and Osborne, 1978; Dandoy et al., 1987; Waterworth et al., 2010, 2015; Chen et al., 2012; Bueso et al., 2014). The observed enrichment of seed related *cis*-elements in the promoters of both *RPA1C* and *RPA1E* raises the question of whether RPA could play a role in seed quality and longevity.

### Summary

We describe in this study the evolution of plant RPA1 family and the unique sequence variations that can be used to categorize RPA1 sequences into three general groups in plants, RPA1A, RPA1B, and RPA1C. Our data suggest that these three groups of plant RPA sequences evolved from two groups of green algal RPA1 progenitor sequences, RPA1B-like and RPA1A/C-like sequences, by losing and gaining unique motifs, domains, and subdomains (Figure 5).

The unique sequence variations that exist within each group of plant RPA1 can be employed to predict biochemical function of RPA1 genes from newly sequenced genomes. These predictions will be valuable in future biochemical characterization studies of RPA complexes, which are ultimately necessary to accurately determine RPA functions in plants. Since the expansion of RPA1B and RPA1C into RPA1D and RPA1E respectively, appears to have occurred early and specifically in the *Brassicacea* family, these sequences could also prove useful in determining phylogenetic relationships within this family.

## Author contributions

BA designed and carried out the experiments and wrote the paper. KC designed experiments and wrote the paper.

## Funding

This work was supported by the National Science Foundation [grant number MCB-0818603]; National Science Foundation ADVANCE [grant number UNH147493] to KC; and the Dissertation Year Fellowship from the University of New Hampshire graduate school to BA. Partial funding was provided by the New Hampshire Agricultural Experiment Station. This is Scientific Contribution Number 2654. This work was supported by the USDA National Institute of Food and Agriculture HATCH Project NH00543.

### Conflict of interest statement

The authors declare that the research was conducted in the absence of any commercial or financial relationships that could be construed as a potential conflict of interest.

## Abbreviations

RPA: Replication Protein A
DSB: Double Strand Break
DBD: DNA Binding Domain
ATM: Ataxia Telangiectasia Mutated
ATR: Ataxia Telangiectasia Rad3-Related
ssDNA: single-stranded DNA.

## References

[B1] Aklilu B. B. Soderquist R. S. Culligan K. M. (2014). Genetic analysis of the Replication Protein A large subunit family in Arabidopsis reveals unique and overlapping roles in DNA repair, meiosis and DNA replication. Nucleic Acids Res. 42, 3104–3118. 10.1093/nar/gkt1292 24335281 PMC3950690

[B2] Armstrong S. J. Franklin F. C. Jones G. H. (2001). Nucleolus-associated telomere clustering and pairing precede meiotic chromosome synapsis in *Arabidopsis thaliana*. J. Cell Sci. 114(Pt 23), 4207–4217. Available online at: http://jcs.biologists.org/content/114/23/4207.article-info 11739653 10.1242/jcs.114.23.4207

[B3] Bastin-Shanower S. A. Brill S. J. (2001). Functional analysis of the four DNA binding domains of replication protein A. The role of RPA2 in ssDNA binding. J. Biol. Chem. 276, 36446–36453. 10.1074/jbc.M104386200 11479296 PMC2796477

[B4] Binz S. K. Sheehan A. M. Wold M. S. (2004). Replication protein A phosphorylation and the cellular response to DNA damage. DNA Repair 3, 1015–1024. 10.1016/j.dnarep.2004.03.028 15279788

[B5] Blackwell L. J. Borowiec J. A. (1994). Human replication protein A binds single-stranded DNA in two distinct complexes. Mol. Cell. Biol. 14, 3993–4001. 10.1128/MCB.14.6.3993 8196638 PMC358765

[B6] Bochkarev A. Pfuetzner R. A. Edwards A. M. Frappier L. (1997). Structure of the single-stranded-DNA-binding domain of replication protein A bound to DNA. Nature 385, 176–181. 10.1038/385176a0 8990123

[B7] Bochkareva E. Belegu V. Korolev S. Bochkarev A. (2001). Structure of the major single-stranded DNA-binding domain of replication protein A suggests a dynamic mechanism for DNA binding. EMBO J. 20, 612–618. 10.1093/emboj/20.3.612 11157767 PMC133470

[B8] Bochkareva E. Kaustov L. Ayed A. Yi G. S. Lu Y. Pineda-Lucena A.. (2005). Single-stranded DNA mimicry in the p53 transactivation domain interaction with replication protein A. Proc. Natl. Acad. Sci. U.S.A. 102, 15412–15417. 10.1073/pnas.0504614102 16234232 PMC1266094

[B9] Bochkareva E. Korolev S. Bochkarev A. (2000). The role for zinc in replication protein A. J. Biol. Chem. 275, 27332–27338. 10.1074/jbc.m000620200 10856290

[B10] Brinster R. L. Allen J. M. Behringer R. R. Gelinas R. E. Palmiter R. D. (1988). Introns increase transcriptional efficiency in transgenic mice. Proc. Natl. Acad. Sci. U.S.A. 85, 836–840. 10.1073/pnas.85.3.836 3422466 PMC279650

[B11] Bueso E. Ibañez C. Sayas E. Muñoz-Bertomeu J. Gonzalez-Guzmán M. Rodriguez P. L. (2014). A forward genetic approach in *Arabidopsis thaliana* identifies a RING-type ubiquitin ligase as a novel determinant of seed longevity. Plant Sci. 215–216, 110–116. 10.1016/j.plantsci.2013.11.004 24388521

[B12] Bulmer M. (1988). Are codon usage patterns in unicellular organisms determined by selection-mutation balance. Evol. Biol. 1, 15 10.1046/j.1420-9101.1988.1010015.x

[B13] Cai L. Roginskaya M. Qu Y. Yang Z. Xu Y. Zou Y. (2007). Structural characterization of human RPA sequential binding to single-stranded DNA using ssDNA as a molecular ruler. Biochemistry 46, 8226–8233. 10.1021/bi7004976 17583916 PMC2553558

[B14] Callis J. Fromm M. Walbot V. (1987). Introns increase gene expression in cultured maize cells. Genes Dev. 1, 1183–1200. 10.1101/gad.1.10.1183 2828168

[B15] Casneuf T. De Bodt S. Raes J. Maere S. Van de Peer Y. (2006). Nonrandom divergence of gene expression following gene and genome duplications in the flowering plant *Arabidopsis thaliana*. Genome Biol. 7:R13. 10.1186/gb-2006-7-2-r13 16507168 PMC1431724

[B16] Castillo-Davis C. I. Hartl D. L. Achaz G. (2004). cis-Regulatory and protein evolution in orthologous and duplicate genes. Genome Res. 14, 1530–1536. 10.1101/gr.2662504 15256508 PMC509261

[B17] Chang Y. Gong L. Yuan W. Li X. Chen G. Li X.. (2009). Replication protein A (RPA1a) is required for meiotic and somatic DNA repair but is dispensable for DNA replication and homologous recombination in rice. Plant Physiol. 151, 2162–2173. 10.1104/pp.109.142877 19812186 PMC2785997

[B18] Cheah K. S. Osborne D. J. (1978). DNA lesions occur with loss of viability in embryos of ageing rye seed. Nature 272, 593–599. 10.1038/272593a0 19213149

[B19] Chen H. Chu P. Zhou Y. Li Y. Liu J. Ding Y.. (2012). Overexpression of AtOGG1, a DNA glycosylase/AP lyase, enhances seed longevity and abiotic stress tolerance in Arabidopsis. J. Exp. Bot. 63, 4107–4121. 10.1093/jxb/ers093 22473985

[B20] Chiapello H. Lisacek F. Caboche M. Hénaut A. (1998). Codon usage and gene function are related in sequences of *Arabidopsis thaliana*. Gene 209, GC1–GC38. 10.1016/S0378-1119(97)00671-9 9583944

[B21] Cui L. Wall P. K. Leebens-Mack J. H. Lindsay B. G. Soltis D. E. Doyle J. J.. (2006). Widespread genome duplications throughout the history of flowering plants. Genome Res. 16, 738–749. 10.1101/gr.4825606 16702410 PMC1479859

[B22] Culligan K. M. Robertson C. E. Foreman J. Doerner P. Britt A. B. (2006). ATR and ATM play both distinct and additive roles in response to ionizing radiation. Plant J. 48, 947–961. 10.1111/j.1365-313X.2006.02931.x 17227549

[B23] Dandoy E. Schnys R. Deltour R. Verly W. G. (1987). Appearance and repair of apurinic/apyrimidinic sites in DNA during early germination of *Zea mays*. Mutat. Res. Fund. Mol. Mech. Muta. 181, 57–60. 10.1016/0027-5107(87)90287-9

[B24] Daughdrill G. W. Ackerman J. Isern N. G. Botuyan M. V. Arrowsmith C. Wold M. S.. (2001). The weak interdomain coupling observed in the 70 kDa subunit of human replication protein A is unaffected by ssDNA binding. Nucleic Acids Res. 29, 3270–3276. 10.1093/nar/29.15.3270 11470885 PMC55822

[B25] Dong J. Park J. S. Lee S. H. (1999). *In vitro* analysis of the zinc-finger motif in human replication protein A. Biochem. J. 337(Pt 2), 311–317. 9882630 PMC1219967

[B26] Drummond D. A. Bloom J. D. Adami C. Wilke C. O. Arnold F. H. (2005). Why highly expressed proteins evolve slowly. Proc. Natl. Acad. Sci. U.S.A. 102, 14338–14343. 10.1073/pnas.0504070102 16176987 PMC1242296

[B27] Duncker B. P. Davies P. L. Walker V. K. (1997). Introns boost transgene expression in *Drosophila melanogaster*. Mol. Gen. Genet. 254, 291–296. 10.1007/s004380050418 9150263

[B28] Duret L. Mouchiroud D. (1999). Expression pattern and, surprisingly, gene length shape codon usage in Caenorhabditis, Drosophila, and Arabidopsis. Proc. Natl. Acad. Sci. U.S.A. 96, 4482–4487. 10.1073/pnas.96.8.4482 10200288 PMC16358

[B29] Eschbach V. Kobbe D. (2014). Different replication protein A complexes of *Arabidopsis thaliana* have different DNA-binding properties as a function of heterotrimer composition. Plant Cell Physiol. 55, 1460–1472. 10.1093/pcp/pcu076 24880780

[B30] Gamsjaeger R. Liew C. K. Loughlin F. E. Crossley M. Mackay J. P. (2007). Sticky fingers: zinc-fingers as protein-recognition motifs. Trends Biochem. Sci. 32, 63–70. 10.1016/j.tibs.2006.12.007 17210253

[B31] Ganpudi A. L. Schroeder D. F. (2011). UV damaged DNA repair & tolerance in plants, in Selected Topics in DNA Repair, ed Chen C. (San Diego, CA: InTech), 73–96.

[B32] Gomes X. V. Wold M. S. (1996). Functional domains of the 70-kilodalton subunit of human replication protein A. Biochemistry 35, 10558–10568. 10.1021/bi9607517 8756712

[B33] Goodstein D. M. Shu S. Howson R. Neupane R. Hayes R. D. Fazo J.. (2012). Phytozome: a comparative platform for green plant genomics. Nucleic Acids Res. 40, D1178–D1186. 10.1093/nar/gkr944 22110026 PMC3245001

[B34] Haring S. J. Mason A. C. Binz S. K. Wold M. S. (2008). Cellular functions of human RPA1. Multiple roles of domains in replication, repair, and checkpoints. J. Biol. Chem. 283, 19095–19111. 10.1074/jbc.M800881200 18469000 PMC2441558

[B35] Hass C. S. Lam K. Wold M. S. (2012). Repair-specific functions of replication protein A. J. Biol. Chem. 287, 3908–3918. 10.1074/jbc.M111.287441 22179778 PMC3281679

[B36] Higo K. Ugawa Y. Iwamoto M. Korenaga T. (1999). Plant cis-acting regulatory DNA elements (PLACE) database: 1999. Nucleic Acids Res. 27, 297–300. 10.1093/nar/27.1.297 9847208 PMC148163

[B37] Hruz T. Laule O. Szabo G. Wessendorp F. Bleuler S. Oertle L.. (2008). Genevestigator v3: a reference expression database for the meta-analysis of transcriptomes. Adv. Bioinformatics 2008:420747. 10.1155/2008/420747 19956698 PMC2777001

[B38] Hyun T. K. Eom S. H. Han X. Kim J. S. (2014). Evolution and expression analysis of the soybean glutamate decarboxylase gene family. J. Biosci. 39, 899–907. 10.1007/s12038-014-9484-2 25431418

[B39] Iftode C. Daniely Y. Borowiec J. A. (1999). Replication protein A (RPA): the eukaryotic SSB. Crit. Rev. Biochem. Mol. Biol. 34, 141–180. 10.1080/10409239991209255 10473346

[B40] Ikemura T. (1985). Codon usage and tRNA content in unicellular and multicellular organisms. Mol. Biol. Evol. 2, 13–34. 3916708 10.1093/oxfordjournals.molbev.a040335

[B41] Ishibashi T. Kimura S. Sakaguchi K. (2006). A higher plant has three different types of RPA heterotrimeric complex. J. Biochem. 139, 99–104. 10.1093/jb/mvj014 16428324

[B42] Jacobs D. M. Lipton A. S. Isern N. G. Daughdrill G. W. Lowry D. F. Gomes X.. (1999). Human replication protein A: global fold of the N-terminal RPA-70 domain reveals a basic cleft and flexible C-terminal linker. J. Biomol. NMR 14, 321–331. 10.1023/A:1008373009786 10526407

[B43] Jones J. S. Weber S. Prakash L. (1988). The *Saccharomyces cerevisiae* RAD18 gene encodes a protein that contains potential zinc finger domains for nucleic acid binding and a putative nucleotide binding sequence. Nucleic Acids Res. 16, 7119–7131. 10.1093/nar/16.14.7119 2970061 PMC338355

[B44] Juneau K. Miranda M. Hillenmeyer M. E. Nislow C. Davis R. W. (2006). Introns regulate RNA and protein abundance in yeast. Genetics 174, 511–518. 10.1534/genetics.106.058560 16816425 PMC1569799

[B45] Karlson D. Imai R. (2003). Conservation of the cold shock domain protein family in plants. Plant Physiol. 131, 12–15. 10.1104/pp.014472 12529510 PMC1540277

[B46] Karlson D. Nakaminami K. Toyomasu T. Imai R. (2002). A cold-regulated nucleic acid-binding protein of winter wheat shares a domain with bacterial cold shock proteins. J. Biol. Chem. 277, 35248–35256. 10.1074/jbc.M205774200 12122010

[B47] Kim C. Paulus B. F. Wold M. S. (1994). Interactions of human replication protein A with oligonucleotides. Biochemistry 33, 14197–14206. 10.1021/bi00251a031 7947831

[B48] Kim C. Snyder R. O. Wold M. S. (1992). Binding properties of replication protein A from human and yeast cells. Mol. Cell. Biol. 12, 3050–3059. 10.1128/MCB.12.7.3050 1320195 PMC364519

[B49] Kim Y. O. Kang H. (2006). The role of a zinc finger-containing glycine-rich RNA-binding protein during the cold adaptation process in *Arabidopsis thaliana*. Plant Cell Physiol. 47, 793–798. 10.1093/pcp/pcj047 16608866

[B50] Kim Y. O. Kim J. S. Kang H. (2005). Cold-inducible zinc finger-containing glycine-rich RNA-binding protein contributes to the enhancement of freezing tolerance in *Arabidopsis thaliana*. Plant J. 42, 890–900. 10.1111/j.1365-313X.2005.02420.x 15941401

[B51] Kim Y. O. Pan S. Jung C. H. Kang H. (2007). A zinc finger-containing glycine-rich RNA-binding protein, atRZ-1a, has a negative impact on seed germination and seedling growth of *Arabidopsis thaliana* under salt or drought stress conditions. Plant Cell Physiol. 48, 1170–1181. 10.1093/pcp/pcm087 17602187

[B52] Krishna S. S. Majumdar I. Grishin N. V. (2003). Structural classification of zinc fingers: survey and summary. Nucleic Acids Res. 31, 532–550. 10.1093/nar/gkg161 12527760 PMC140525

[B53] Krylov D. M. Wolf Y. I. Rogozin I. B. Koonin E. V. (2003). Gene loss, protein sequence divergence, gene dispensability, expression level, and interactivity are correlated in eukaryotic evolution. Genome Res. 13, 2229–2235. 10.1101/gr.1589103 14525925 PMC403683

[B54] Langelier M. F. Planck J. L. Roy S. Pascal J. M. (2011). Crystal structures of poly(ADP-ribose) polymerase-1 (PARP-1) zinc fingers bound to DNA: structural and functional insights into DNA-dependent PARP-1 activity. J. Biol. Chem. 286, 10690–10701. 10.1074/jbc.m110.202507 21233213 PMC3060520

[B55] Lao Y. Gomes X. V. Ren Y. Taylor J. S. Wold M. S. (2000). Replication protein A interactions with DNA. III. Molecular basis of recognition of damaged DNA. Biochemistry 39, 850–859. 10.1021/bi991704s 10653628

[B56] Le Hir H. Nott A. Moore M. J. (2003). How introns influence and enhance eukaryotic gene expression. Trends Biochem. Sci. 28, 215–220. 10.1016/S0968-0004(03)00052-5 12713906

[B57] Li X. Chang Y. Xin X. Zhu C. Li X. Higgins J. D.. (2013). Replication protein A2c coupled with replication protein A1c regulates crossover formation during meiosis in rice. Plant Cell 25, 3885–3899. 10.1105/tpc.113.118042 24122830 PMC3877797

[B58] Lin Y. L. Chen C. Keshav K. F. Winchester E. Dutta A. (1996). Dissection of functional domains of the human DNA replication protein complex replication protein A. J. Biol. Chem. 271, 17190–17198. 10.1074/jbc.271.29.17190 8663296

[B59] Lin Y. L. Shivji M. K. Chen C. Kolodner R. Wood R. D. Dutta A. (1998). The evolutionarily conserved zinc finger motif in the largest subunit of human replication protein A is required for DNA replication and mismatch repair but not for nucleotide excision repair. J. Biol. Chem. 273, 1453–1461. 10.1074/jbc.273.3.1453 9430682

[B60] Liu J. S. Kuo S. R. Melendy T. (2006). DNA damage-induced RPA focalization is independent of gamma-H2AX and RPA hyper-phosphorylation. J. Cell. Biochem. 99, 1452–1462. 10.1002/jcb.21066 16927366

[B61] Liu Q. Feng Y. Zhao X. Dong H. Xue Q. (2004). Synonymous codon usage bias in *Oryza sativa*. Plant Sci. 167, 101–105. 10.1016/j.plantsci.2004.03.003

[B62] Lockton S. Gaut B. S. (2005). Plant conserved non-coding sequences and paralogue evolution. Trends Genet. 21, 60–65. 10.1016/j.tig.2004.11.013 15680516

[B63] Longhese M. P. Plevani P. Lucchini G. (1994). Replication factor A is required *in vivo* for DNA replication, repair, and recombination. Mol. Cell. Biol. 14, 7884–7890. 10.1128/MCB.14.12.7884 7969128 PMC359327

[B64] Louis E. J. (2007). Evolutionary genetics: making the most of redundancy. Nature 449, 673–674. 10.1038/449673a 17928851

[B65] Ly V. Hatherell A. Kim E. Chan A. Belmonte M. F. Schroeder D. F. (2013). Interactions between Arabidopsis DNA repair genes UVH6, DDB1A, and DDB2 during abiotic stress tolerance and floral development. Plant Sci. 213, 88–97. 10.1016/j.plantsci.2013.09.004 24157211

[B66] Lynch M. Conery J. S. (2000). The evolutionary fate and consequences of duplicate genes. Science 290, 1151–1155. 10.1126/science.290.5494.1151 11073452

[B67] Marais G. Duret L. (2001). Synonymous codon usage, accuracy of translation, and gene length in *Caenorhabditis elegans*. J. Mol. Evol. 52, 275–280. 10.1007/s002390010155 11428464

[B68] Marchler-Bauer A. Lu S. Anderson J. B. Chitsaz F. Derbyshire M. K. DeWeese-Scott C.. (2011). CDD: a Conserved Domain Database for the functional annotation of proteins. Nucleic Acids Res. 39, D225–D229. 10.1093/nar/gkq1189 21109532 PMC3013737

[B69] Marwedel T. Ishibashi T. Lorbiecke R. Jacob S. Sakaguchi K. Sauter M. (2003). Plant-specific regulation of replication protein A2 (OsRPA2) from rice during the cell cycle and in response to ultraviolet light exposure. Planta 217, 457–465. 10.1007/s00425-003-1001-z 14520573

[B70] Moore R. C. Purugganan M. D. (2005). The evolutionary dynamics of plant duplicate genes. Curr. Opin. Plant Biol. 8, 122–128. 10.1016/j.pbi.2004.12.001 15752990

[B71] Niu D. K. Yang Y. F. (2011). Why eukaryotic cells use introns to enhance gene expression: splicing reduces transcription-associated mutagenesis by inhibiting topoisomerase I cutting activity. Biol. Direct 6:24. 10.1186/1745-6150-6-24 21592350 PMC3118952

[B72] Osman K. Sanchez-Moran E. Mann S. C. Jones G. H. Franklin F. C. (2009). Replication protein A (AtRPA1a) is required for class I crossover formation but is dispensable for meiotic DNA break repair. EMBO J. 28, 394–404. 10.1038/emboj.2008.295 19153602 PMC2646153

[B73] Pál C. Papp B. Hurst L. D. (2001). Highly expressed genes in yeast evolve slowly. Genetics 158, 927–931. Available online at: http://genetics.org/content/158/2/927.long 11430355 10.1093/genetics/158.2.927 PMC1461684

[B74] Rajavashisth T. B. Taylor A. K. Andalibi A. Svenson K. L. Lusis A. J. (1989). Identification of a zinc finger protein that binds to the sterol regulatory element. Science 245, 640–643. 10.1126/science.2562787 2562787

[B75] Ren X. Y. Vorst O. Fiers M. W. Stiekema W. J. Nap J. P. (2006). In plants, highly expressed genes are the least compact. Trends Genet. 22, 528–532. 10.1016/j.tig.2006.08.008 16934358

[B76] Rogelj B. Godin K. S. Shaw C. E. Ule J. (2011). The functions of glycine-rich regions in TDP-43, FUS and related RNA-binding proteins, in RNA Binding Proteins, ed Lorkovic Z. J. (Austin, TX: 'Landes Bioscience and Springer Science+Business Media), 1–17.

[B77] Roy S. (2014). Maintenance of genome stability in plants: repairing DNA double strand breaks and chromatin structure stability. Front. Plant Sci. 5:487. 10.3389/fpls.2014.00487 25295048 PMC4172009

[B78] Sablok G. Wu X. Kuo J. Nayak K. C. Baev V. Varotto C.. (2013). Combinational effect of mutational bias and translational selection for translation efficiency in tomato (Solanum lycopersicum) cv. Micro-Tom. Genomics 101, 290–295. 10.1016/j.ygeno.2013.02.008 23474140

[B79] Shabalina S. A. Ogurtsov A. Y. Spiridonov A. N. Novichkov P. S. Spiridonov N. A. Koonin E. V. (2010). Distinct patterns of expression and evolution of intronless and intron-containing mammalian genes. Mol. Biol. Evol. 27, 1745–1749. 10.1093/molbev/msq086 20360214 PMC2908711

[B80] Shultz R. W. Tatineni V. M. Hanley-Bowdoin L. Thompson W. F. (2007). Genome-wide analysis of the core DNA replication machinery in the higher plants Arabidopsis and rice. Plant Physiol. 144, 1697–1714. 10.1104/pp.107.101105 17556508 PMC1949880

[B81] Simillion C. Vandepoele K. Van Montagu M. C. Zabeau M. Van de Peer Y. (2002). The hidden duplication past of *Arabidopsis thaliana*. Proc. Natl. Acad. Sci. U.S.A. 99, 13627–13632. 10.1073/pnas.212522399 12374856 PMC129725

[B82] Singh S. K. Roy S. Choudhury S. R. Sengupta D. N. (2010). DNA repair and recombination in higher plants: insights from comparative genomics of Arabidopsis and rice. BMC Genomics 11:443. 10.1186/1471-2164-11-443 20646326 PMC3091640

[B83] Takashi Y. Kobayashi Y. Tanaka K. Tamura K. (2009). Arabidopsis replication protein A 70a is required for DNA damage response and telomere length homeostasis. Plant Cell Physiol. 50, 1965–1976. 10.1093/pcp/pcp140 19812063

[B84] Tamura K. Peterson D. Peterson N. Stecher G. Nei M. Kumar S. (2011). MEGA5: molecular evolutionary genetics analysis using maximum likelihood, evolutionary distance, and maximum parsimony methods. Mol. Biol. Evol. 28, 2731–2739. 10.1093/molbev/msr121 21546353 PMC3203626

[B85] Theobald D. L. Mitton-Fry R. M. Wuttke D. S. (2003). Nucleic acid recognition by OB-fold proteins. Annu. Rev. Biophys. Biomol. Struct. 32, 115–133. 10.1146/annurev.biophys.32.110601.142506 12598368 PMC1564333

[B86] Tzfati Y. Abeliovich H. Avrahami D. Shlomai J. (1995). Universal minicircle sequence binding protein, a CCHC-type zinc finger protein that binds the universal minicircle sequence of trypanosomatids. Purification and characterization. J. Biol. Chem. 270, 21339–21345. 10.1074/jbc.270.36.21339 7545668

[B87] Umezu K. Sugawara N. Chen C. Haber J. E. Kolodner R. D. (1998). Genetic analysis of yeast RPA1 reveals its multiple functions in DNA metabolism. Genetics 148, 989–1005. 9539419 10.1093/genetics/148.3.989 PMC1460019

[B88] Vassin V. M. Wold M. S. Borowiec J. A. (2004). Replication protein A (RPA) phosphorylation prevents RPA association with replication centers. Mol. Cell. Biol. 24, 1930–1943. 10.1128/MCB.24.5.1930-1943.2004 14966274 PMC350552

[B89] Vision T. J. Brown D. G. Tanksley S. D. (2000). The origins of genomic duplications in Arabidopsis. Science 290, 2114–2117. 10.1126/science.290.5499.2114 11118139

[B90] Walther A. P. Gomes X. V. Lao Y. Lee C. G. Wold M. S. (1999). Replication protein A interactions with DNA. 1. Functions of the DNA-binding and zinc-finger domains of the 70-kDa subunit. Biochemistry 38, 3963–3973. 10.1021/bi982370u 10194308

[B91] Waterworth W. M. Bray C. M. West C. E. (2015). The importance of safeguarding genome integrity in germination and seed longevity. J. Exp. Bot. 66, 3549–3558. 10.1093/jxb/erv080 25750428

[B92] Waterworth W. M. Drury G. E. Bray C. M. West C. E. (2011). Repairing breaks in the plant genome: the importance of keeping it together. New Phytol. 192, 805–822. 10.1111/j.1469-8137.2011.03926.x 21988671

[B93] Waterworth W. M. Masnavi G. Bhardwaj R. M. Jiang Q. Bray C. M. West C. E. (2010). A plant DNA ligase is an important determinant of seed longevity. Plant J. 63, 848–860. 10.1111/j.1365-313X.2010.04285.x 20584150

[B94] Winter D. Vinegar B. Nahal H. Ammar R. Wilson G. V. Provart N. J. (2007). An “Electronic Fluorescent Pictograph” browser for exploring and analyzing large-scale biological data sets. PLoS ONE 2:e718. 10.1371/journal.pone.0000718 17684564 PMC1934936

[B95] Wobbe C. R. Weissbach L. Borowiec J. A. Dean F. B. Murakami Y. Bullock P.. (1987). Replication of simian virus 40 origin-containing DNA *in vitro* with purified proteins. Proc. Natl. Acad. Sci. U.S.A. 84, 1834–1838. 10.1073/pnas.84.7.1834 3031654 PMC304535

[B96] Wold M. S. (1997). Replication protein A: a heterotrimeric, single-stranded DNA-binding protein required for eukaryotic DNA metabolism. Annu. Rev. Biochem. 66, 61–92. 10.1146/annurev.biochem.66.1.61 9242902

[B97] Wright S. I. Yau C. B. Looseley M. Meyers B. C. (2004). Effects of gene expression on molecular evolution in *Arabidopsis thaliana* and *Arabidopsis lyrata*. Mol. Biol. Evol. 21, 1719–1726. 10.1093/molbev/msh191 15201397

[B98] Xu X. Vaithiyalingam S. Glick G. G. Mordes D. A. Chazin W. J. Cortez D. (2008). The basic cleft of RPA70N binds multiple checkpoint proteins, including RAD9, to regulate ATR signaling. Mol. Cell. Biol. 28, 7345–7353. 10.1128/MCB.01079-08 18936170 PMC2593429

[B99] Zhang J. (2003). Evolution by gene duplication: an update. Trends Ecol. Evol. 18, 292–298. 10.1016/S0169-5347(03)00033-8

[B100] Zou Y. Liu Y. Wu X. Shell S. M. (2006). Functions of human replication protein A (RPA): from DNA replication to DNA damage and stress responses. J. Cell. Physiol. 208, 267–273. 10.1002/jcp.20622 16523492 PMC3107514

