# Supplementary material for: Molecular Evolution and Functional Diversification of Replication Protein A1 in Plants
Source: Front Plant Sci. 2016 Jan 29;7:33. doi: 10.3389/fpls.2016.00033 (PMC4731521; doi:10.3389/fpls.2016.00033)
Supplement: Supplementary file 1 [file Table1.PDF]

Plant species	RPA1 NCBI protein ID	Similarity to the Arabidopsis RPA1	Percent similarity	E-value	Intron number	Designation
***Arabidopsis thaliana* (Arabidopsis)**	NP_178690.1	RPA1A	100%	0.0	2	*At* RPA1A*
	NP_196419.1	RPA1B	100%	0.0	9	*At* RPA1B*
	NP_199353.1	RPA1C	100%	0.0	1	*At* RPA1C*
	NP_200908.1	RPA1D	100%	0.0	10	*At* RPA1D*
	NP_567576.2	RPA1E	100%	0.0	1	*At* RPA1E*
Arabidopsis lyrata	XP_002883738.1	RPA1A	97%	0.0	2	*Al* RPA1A
	XP_002871302.1	RPA1B	96%	0.0	9	*Al* RPA1B
	XP_002890942.1	RPA1C	84%	0.0	2	*Al* RPA1C
	XP_002866409.1	RPA1D	95%	0.0	10	*Al* RPA1D
	XP_002867952.1	RPA1E	87%	0.0	1	*Al* RPA1E
***Caspella* rubella**	EOA30084.1	RPA1A	94%	0.0	2	*Cr* RPA1A
	EOA20180.1	RPA1B	94%	0.0	9	*Cr* RPA1B
	EOA14703.1	RPA1C	81%	0.0	2	*Cr* RPA1C
	EOA13017.1	RPA1D	94%	0.0	10	*Cr* RPA1D
	EOA15986.1	RPA1E	83%	0.0	1	*Cr* RPA1E
***Solanum* lycopursicum (Tomato)**	XP_004239312.1	RPA1A	69%	0.0	1	*Sl* RPA1A
	XP_004236053.1	RPA1B	73%	0.0	11	*Sl* RPA1B
	XP_004245920.1	RPA1C	50%	0.0	1	*Sl* RPA1C
***Medicago truncatula* (Barel clover)**	XP_003599848.1	RPA1A	63%	0.0	6	*Mt* RPA1A
	XP_003612608.1	RPA1C	50%	0.0	6	*Mt* RPA1C
***Vitis* vinifera (Grape)**	XP_002278273.1	RPA1A	73%	0.0	1	*Vv* RPA1A
	XP_002283959.1	RPA1B	71%	0.0	11	*Vv* RPA1B
	XP_002264009.1	RPA1C	55%	0.0	1	*Vv* RPA1C
**Theobroma cacao (Cacao)**	XP_007012690.1	RPA1A	70%	0.0	1	*Ts* RPA1A
	XP_007037579.1	RPA1B	67%	0.0	11	*Ts* RPA1B
	XP_007040824.1	RPA1C	55%	0.0	1	*Tc* RPA1C
***Fragaria* Vesca (Strawberry)**	XP_004287611.1	RPA1A	66%	0.0	1	*Fv* RPA1A
	XP_004301112.1	RPA1B	67%	0.0	11	*Fv* RPA1B
	XP_004301636.1	RPA1C	48%	0.0	1	*Fv* RPA1C
***Cucumis sativus* (Cucumber)**	XP_004141065.1	RPA1A	68%	0.0	2	*Cs* RPA1A
	XP_004138198.1	RPA1B	70%	0.0	11	*Cs* RPA1B
	XP_004146122.1	RPA1C	55%	0.0	1	*Cs* RPA1C
***Glycin max* (Soybean)**	XP_003546476.1	RPA1A	68%	0.0	1	*Gm* RPA1A
	XP_003524615.1	RPA1B	68%	0.0	11	*Gm* RPA1B-1
	XP_006600581.1	RPA1B	68%	0.0	12	*Gm* RPA1B-2
	XP_003533460.1	RPA1C	50%	0.0	1	*Gm* RPA1C

Supplementary Table S1. List of plant and algal species and their RPA1 proteins. The proteins were identified using BLAST hosted by NCBI. Orthologous Arabidopsis RPA1 proteins were employed for the protein search. Reverse Blast (RB) was used to confirm orthology. Asterics indicate previously characterized and designated RPA1proteins

Supplementary Table S1. Continued

Plant species	RPA1 NCBI protein ID	Similarity to the Arabidopsis RPA1	Percent similarity	E-value	Intron number	Designation
Ricinus communis (Castor oil plant)	XP_002514062.1	RPA1A	69%	0.0	1	*Rc* RPA1A
	XP_002514651.1	RPA1B	70%	0.0	11	*Rc* RPA1B
	XP_002519884.1	RPA1C	52%	0.0	3	*Rc* RPA1C
Populus trichocarpa (California poplar)	XP_006372003.1	RPA1A	67%	0.0	1	*Pt* RPA1A
	XP_006374397.1	RPA1B	72%	0.0	12	*Pt* RPA1B
	XP_002304377.1	RPA1C	61	0.0	1	*Pt* RPA1C
Prunus persica (Peach)	XP_007201934.1	RPA1A	67%	0.0	1	*Pp* RPA1A
	XP_007210879.1	RPA1B	70%	0.0	11	*Pp RPA1B*
	XP_007211093.1	RPA1C	50%	0.0	1	*Pp* RPA1C
Zea mays (corn)	NP_001169500.1	RPA1A	58%	0.0	1	*Zm* RPA1A
	NP_001141474.1	RPA1B	62%	0.0	11	*Zm* RPA1B-1
	NP_001147118.1	RPA1B	62%	0.0	11	*Zm* RPA1B-2
	NP_001151792.1	RPA1C	41%	0.0	1	*Zm* RPA1C
Sorghum bicolar (Sorghum)	XP_002454649.1	RPA1A	58%	0.0	1	*Sb* RPA1A
	XP_002468278.1	RPA1B	63%	0.0	11	*Sb* RPA1B
	XP_002439140.1	RPA1C	44%	0.0	1	*Sb* RPA1C-1
	XP_002452128.1	RPA1C	39%	0.0	1	*Sb* RPA1C-2
	XP_002439139.1	RPA1C	43%	7e-174	0	*Sb* RPA1C-3
	XP_002439138.1	RPA1C	48%	7e-144	5	*Sb* RPA1C-4
Setaria italica (Millet)	XP_004954095.1	RPA1A	59%	0.0	1	*Si* RPA1A
	XP_004985197.1	RPA1B	63%	0.0	11	*Si* RPA1B
	XP_004960524.1	RPA1C	45%	0.0	1	*Si* RPA1C-1
	XP_004963448.1	RPA1C	40%	0.0	7	*Si* RPA1C-2
Oryza sativa (Rice)	NP_001048287.1	RPA1A	59%	0.0	1	*Os* RPA1A*
	NP_001049369.1	RPA1B	65%	0.0	11	*Os* RPA1B*
	NP_001054445.1	RPA1C	42%	0.0	1	*Os* RPA1C*
Brachypodium *distachyon*	XP_003570523.1	RPA1A	57%	0.0	1	*Bd* RPA1A-1
	XP_003558501.1	RPA1A	55%	0.0	1	*Bd* RPA1A-2
	XP_003561889.1	RPA1B	64%	0.0	11	*Bd* dRPA1B
	XP_003569087.1	RPA1C	43%	0.0	1	*Bd* RPA1C
Selaginella moellendorffii	XP_002968459.1	RPA1A	52%	0.0	2	*Sm* RPA1A-1
	XP_002970633.1	RPA1A	47%	0.0	2	*Sm* RPA1A-2
	XP_002981625.1	RPA1B	52%	0.0	11	*Sm* RPA1B-1
	XP_002963163.1	RPA1B	52%	0.0	11	*Sm* RPA1B-2
Physcomitrella patens	XP_001777498.1	RPA1A	56%	0.0	4	*Pp* RPA1A-1
	XP_001775481.1	RPA1A	54%	0.0	4	*Pp* RPA1A-2
	XP_001779367.1	RPA1B	52%	1e-176	8	*Pp* RPA1B

Supplementary Table S1. Continued

Unicellular green algae species	RPA1 NCBI protein ID	Similarity to the Arabidopsis RPA1	Percent similarity	E-value	Intron number	Designation
Ostreococcus lucimarinus	XP_001422250.1	RPA1B	36%	2e-210	2	*Ol* RPA1B
	XP_001421229.1	RPA1E	32%	1e-110	1	*Ol* RPA1E
Micromonas pusilla	XP_003064598.1	RPA1B	35%	3e-105	3	*Mp* RPA1B
	XP_003057576.1	RPA1C	31%	2e-128	2	*Mp* RPA1C
Volvox cateri	XP_002952179.1	RPA1B	31%	1e-68	10	*Vc* RPA1B
	XP_002949682.1	RPA1C	42%	6e-122	13	*Vc* RPA1C
Chlamydomonas reinhardtii	XP_001698157.1	RPA1B	26%	2e-65	14	*Cr* RPA1B
	XP_001703773.1	RPA1A	42%	2e-124	12	*Cr* RPA1A
Coccomyxa subellipsoida	XP_005644552.1	RPA1B	34%	4e-97	8	*Cs* RPA1B
	XP_005644980.1	RPA1A	35%	1e-132	11	*Cs* RPA1A

Species	Type	NCBI RPA1 protein ID	Similarity to the Arabidopsis RPA1	Percent identity	E-value
Cyanidioschyzon merolae	Unicellular red algae	XP_005535335.1	RPA1A	30%	3e-88
Galdieria sulphuraria	Unicellular red algae	XP_005702788.1	RPA1A	33%	3e-99
Chondrus crispus	Multicellular red algae	XP_005712204.1	RPA1A	30%	2e-87
Ectocarpus siliculosus	Multicellular brown algae	CBJ31747.1	RPA1C	37%	7e-86

Supplementary Table S2. RPA1-like proteins of red and brown algae species . The proteins were identified using BLAST hosted in NCBI. Orthologous Arabidopsis and algae RPA1 proteins were used for the protein search. Reverse BLAST(RB) method was used to confirm orthology.

Species	NCBI RPA1 Protein ID	Similarity to the Arabidopsis RPA1	Percent identity	E-value	Intron number
Homo sapiens	NP_002936.1	RPA1E	35%	3e-119	16
Xenopus tropicalis	NP_001015732.1	RPA1A	35%	2e-177	16
Danio rerio	NP_956105.2	RPA1A	34%	7e-113	16
Drosophila melanogaster	NP_524274.1	RPA1A	33%	8e-103	3
Saccharomyces cerevisiae	NP_009404.1	RPA1C	31%	1e-93	0
Schizosaccharomyces pombe	NP_595092.1	RPA1E	31%	8e-107	1
Neurospora crassa	XP_961333.1	RPA1E	31%	8e-101	3
Ustilago maydis	XP_011388213.1	RPA1E	32%	8e-106	0

Supplementary Table S3. RPA1 proteins of animals and fungal species . The proteins were identified using BLAST hosted in NCBI. Orthologous Arabidopsis RPA1 proteins were used for the protein search. Reverse BLAST(RB) method was used to confirm orthology.

A

	Percentage of amino acids (aa)																				Total # of aa
Species	A	C	D	E	F	G	H	I	K	L	M	N	P	Q	R	S	T	V	W	Y	
Arabidopsis	8.8	2.6	4.4	2.6	2.6	14.0	1.8	1.8	1.8	4.4	2.6	7.9	4.4	5.3	6.1	14.0	5.3	5.3	0.0	4.4	114.0
A. lyrata	8.5	2.5	3.4	2.5	0.8	15.3	0.8	3.4	0.8	4.2	2.5	10.2	5.1	5.1	5.9	14.4	5.1	4.2	0.0	5.1	118.0
C. rubella	7.9	2.6	2.6	5.3	1.8	15.8	1.8	2.6	0.9	3.5	2.6	7.0	5.3	5.3	6.1	13.2	5.3	5.3	0.0	5.3	114.0
Tomato	6.3	2.3	3.3	4.7	5.0	14.3	2.0	3.3	4.0	4.7	3.7	7.3	4.0	8.0	4.0	10.6	5.3	3.0	0.7	3.7	301.0
Strawberry	8.8	4.4	1.3	1.3	0.6	13.1	3.8	3.8	2.5	3.1	1.3	8.8	7.5	4.4	2.5	16.9	3.1	7.5	0.6	5.0	160.0
Cucumber	5.8	3.9	1.3	3.2	3.9	14.3	2.6	2.6	1.9	2.6	1.3	9.1	8.4	3.9	4.5	16.2	7.1	2.6	0.6	3.9	154.0
Soybean	8.6	3.0	3.0	5.6	4.3	12.5	3.0	4.3	5.3	4.0	2.0	5.6	4.3	5.3	3.6	11.2	4.6	4.3	0.7	5.0	303.0
Castor oil plant	8.7	4.4	1.6	4.9	3.3	13.1	3.3	4.9	2.2	4.4	0.5	8.2	5.5	4.4	3.3	15.3	3.8	2.7	1.1	4.4	183.0
Grape	5.3	3.5	1.8	1.8	3.5	18.7	2.9	1.2	2.3	2.9	1.8	7.6	5.8	4.7	3.5	17.0	5.8	5.3	0.6	4.1	171.0
Cacao	6.7	4.9	5.5	3.1	3.1	14.1	3.1	3.7	3.1	2.5	2.5	4.3	4.3	4.3	6.1	13.5	6.7	3.1	0.6	4.9	163.0
California poplar	7.1	4.5	1.3	1.3	3.2	16.8	3.2	1.3	3.2	1.9	3.2	9.0	6.5	6.5	2.6	15.5	3.9	4.5	0.6	3.9	155.0
Peach	5.6	2.2	3.7	6.3	3.7	13.0	1.9	2.6	5.2	4.8	2.6	7.4	4.5	4.5	4.5	10.0	5.9	7.1	0.7	3.7	269.0
Maize	12.5	3.6	1.2	1.2	1.2	13.1	1.2	0.6	0.6	4.2	0.6	7.7	7.1	11.3	4.2	11.9	7.7	4.2	0.0	6.0	168.0
RPA1C-1 Sorghum	11.3	3.2	1.6	1.1	1.6	14.0	1.1	1.1	1.1	3.2	2.2	7.0	7.0	12.4	3.8	14.0	6.5	1.6	0.0	6.5	186.0
RPA1C.2 Sorghum	9.1	3.6	3.6	1.8	3.6	20.0	1.8	2.7	2.7	1.8	1.8	7.3	6.4	8.2	5.5	10.9	3.6	1.8	0.9	2.7	110.0
Sorghum RPA1C-3	10.8	5.4	5.4	1.4	2.7	10.8	2.7	4.1	5.4	6.8	0.0	4.1	4.1	5.4	2.7	13.5	4.1	5.4	2.7	2.7	74.0
Millet RPA1C-1	10.5	4.3	3.1	6.2	2.7	11.7	1.2	2.3	2.7	5.8	3.1	5.1	3.9	7.4	4.7	8.9	5.8	4.3	0.4	5.8	257.0
Millet RPA1C-2	8.5	4.9	8.5	1.2	3.7	11.0	1.2	3.7	3.7	9.8	3.7	2.4	7.3	2.4	6.1	11.0	3.7	2.4	2.4	2.4	82.0
Rice	9.5	6.0	4.0	2.0	3.5	15.4	3.5	2.0	3.5	3.5	2.5	6.0	5.5	8.0	3.0	10.4	5.5	3.0	0.0	3.5	201.0
B. distachyon	13.6	5.0	3.7	0.8	3.7	12.0	2.9	0.8	1.2	3.7	1.7	9.1	6.6	5.8	3.7	12.8	5.0	3.7	0.0	4.1	242.0
Average	8.7	3.8	3.2	2.9	2.9	14.1	2.3	2.6	2.7	4.1	2.1	7.1	5.7	6.1	4.3	13.1	5.2	4.1	0.6	4.3	176.3

B

	Percentage of amino acids (aa)																				Total # of aa
Species	A	C	D	E	F	G	H	I	K	L	M	N	P	Q	R	S	T	V	W	Y	
Arabidopsis	5.2	2.6	4.3	1.7	0.9	19.0	4.3	1.7	2.6	3.4	0.9	6.0	5.2	6.0	6.0	12.9	6.9	5.2	0.0	5.2	116.0
A. lyrata	6.5	2.4	4.1	1.6	0.8	17.9	4.9	2.4	0.0	1.6	1.6	6.5	4.1	5.7	4.9	19.5	6.5	4.1	0.0	4.9	123.0
C. rubella	6.7	2.5	3.4	2.5	0.8	17.6	4.2	2.5	0.8	2.5	0.8	7.6	5.9	5.0	5.9	16.8	4.2	4.2	0.0	5.9	119.0
Average	6.1	2.5	3.9	2.0	0.8	18.2	4.5	2.2	1.1	2.5	1.1	6.7	5.0	5.6	5.6	16.4	5.9	4.5	0.0	5.3	119.3

Supplementary Table S4. Amino acid composition of the C-terminal extension region of RPA1C (A) and RPA1E (B). Highlighted columns show the most abundant amino acids (Glycine and Serine). Analysis was conducted using MEGA5.

Category	*Cis*-elements	Genes					Description
		RPA1A	*RPA1C*	*RPA1E*	*RPA1B*	*RPA1D*	
**Reproductive phase transition and flower related**	ROOTMOTIFTAPOX1	14	1	1	0	4	Motif found both in promoters of rolD.The plant oncogene rolD stimulates the reproductive phase transition in plants
		14	8	2	3	4	
	CARGATCONSENSUS	0	0	0	0	0	"CArG consensus" sequence found in the promoter of Arabidopsis. SOC1 which is the MADS-box flowering time gene.
		0	2	0	0	0	
	WUSATAg	2	0	0	0	0	Target sequence of WUS in the intron of AGAMOUS gene in A. thaliana. AGAMOUS gene is related to floral organ identity.
		2	2	1	0	0	
**Total number of *cis*-elements**		16	1	1	0	4	
		16	12	3	3	4	
**Pollen related**	GTGANTG10	10	2	6	3	4	"GTGA motif" found in the promoter of the tobacco late pollen gene g10.
		10	9	16	12	11	
	POLLEN1LELAT52	11	6	1	4	8	One of two co-dependent regulatory elements responsible for pollen specific activation of tomato lat52 gene.
		11	12	12	13	15	
	POLLEN2LELAT52	0	0	0	0	1	One of two co-dependent regulatory elements responsible for pollen specific activation of tomato lat52 gene.
		0	0	0	0	1	
**Total number of *cis*-elements**		21	8	7	7	13	
		21	21	28	25	27	

Supplementary Table S5. List of *cis*-regulatory elements found within Arabidopsis *RPA1* genes. Numbers indicate frequency of the respective element occurrence in the promoter of each gene. Each cis-element has two rows filled with numbers. The numbers in the upper row are based on promoter sequences of varying length (RPA1A= 1794 bp, RPA1C= 873 bp, RPA1E= 571 bp, RPA1B= 372 bp, RPA1D= 1174 bp) as obtained from TAIR data base. The numbers in the lower row are based on optimized promoter sequence length (equal length, 1794 bp, for each RPA1).

Supplementary Table S5. Continued

Category	*Cis*-elements	Genes					Description
		RPA1A	*RPA1C*	*RPA1E*	*RPA1B*	*RPA1D*	
**Cell cycle and DNA synthesis related**	E2FCONSENSUS	0<br>0	0<br>0	0<br>0	2<br>2	1<br>1	"E2F consensus sequence" of all different E2F-DP-binding motifs found in plants. E2F is a group of genes that codifies a family of transcription factors (TF) in higher eukaryotes. They are involved in the cell cycle regulation and synthesis of DNA.
	MYBCOREATCYCB1	0<br>0	0<br>0	0<br>0	0<br>2	0<br>2	"Myb core" found in the promoter of Arabidopsis Arabidopsis cyclin B1:1 gene.
	TE2F2NTPCNA	0<br>0	0<br>0	0<br>0	1<br>1	0<br>0	"te2f-2" found in the promoter of tobacco PCNA gene. Binding site of Os E2F1 and Os E2F2. Involved in transcriptional activation in actively dividing cells and tissue.
**Total number of cis-elements**		0<br>0	0<br>0	0<br>0	3<br>5	1<br>3	
**Senescence related**	ERELEE4	3<br>3	0<br>0	3<br>3	0<br>0	0<br>0	"ERE (ethylene responsive element)" of tomato E4 and carnation GST1 genes; GST1 is related to senescence.
	LECPLEACS2	2<br>2	0<br>0	0<br>0	0<br>0	0<br>0	Core element in LeCp (tomato Cys protease) binding cis-element) in LeAcs2 gene. LeAcs2 gene codes for an enzyme that catalyzes the synthesis of 1-Aminocyclopropane-1-carboxylic acid (ACC), a precursor for ethylene.
**Total number of cis-elements**		5<br>5	0<br>0	3<br>3	0<br>0	0<br>0	
**Seed development and germination related**	-300CORE	0<br>0	1<br>1	0<br>0	0<br>0	1<br>1	"prolamin box" or P-box; Binds with P-box binding factor (PBF). The prolamin box is found in promoters of many cereal seed storage protein genes.
	-300ELEMENT	3<br>3	5<br>7	0<br>3	1<br>1	2<br>2	Present upstream of the promoter from the B-hordein gene of barley and the alpha-gliadin, gamma-gliadin, and low molecular weight glutenin genes of wheat. These proteins are groups of polymeric storage proteins.

Supplementary Table S5. Continued

Category	*Cis*-elements	Genes					Description
		RPA1A	*RPA1C*	*RPA1E*	*RPA1B*	*RPA1D*	
Seed development and germination related	2SSEEDPROTBANAPA	0	0	0	0	1	Conserved in many storage-protein gene promoters.
		0	0	1	0	1	
	AACACOREOSGLUB1	0	0	0	0	0	Core of AACA motifs found in rice glutelin genes. Involved in controlling the endosperm-specific expression.
		0	0	1	0	0	
	ACGTABOX	0	2	0	0	0	"A-box" according to the nomenclature of ACGT elements by Foster et al. Found in ocs gene. Binding site for the rice bZIP transcriptional activator RITA-1 that is highly expressed during seed development. Also called "G motif" by Toyofuku et al. (1998). G motif is responsible for sugar repression.
		0	2	0	0	0	
	ACGTCBOX	0	2	0	0	0	"C-box" according to the nomenclature of One of ACGT elements. RITA-1 binding site.
		0	0	2	0	0	
	AMYBOX2	0	0	0	0	0	"amylase box"; "amylase element"; Conserved sequence found in 5'upstream region of alpha-amylase gene of rice, wheat, barley.
		0	0	0	1	0	
	CANBNNAPA	0	1	0	0	1	Core of "(CA)n element" in storage protein genes in Brasica napus(B.n.). Embryo- and endosperm-specific transcription of napin (storage protein) gene, napA.
		0	2	1	1	1	
	CEREGLUBOX3PSLEGA	0	0	0	0	0	"Cereal glutenin box" in pea legumin gene (legA). Sequence homologous to the cereal glutenin gene control elements.
		0	1	0	0	0	
	CGACGOSAMY3	1	3	0	0	0	"CGACG element" found in the GC-rich regions of the rice Amy3D and Amy3E amylase genes.
		1	3	5	1	0	
	EBOXBNNAPA	12	2	4	0	4	E-box of napA storage-protein gene of Brassica napus (B.n.). This sequence is also known as RRE (R response element).
		12	14	20	20	10	
	GCN4OSGLUB1	0	0	1	0	0	"GCN4 motif" found in GluB-1 gene in rice. Required for endosperm-specific expression.
		0	0	1	0	0	

Supplementary Table S5. Continued

Category	*Cis*-elements	Genes					Description
Seed development and germination related Seed related	NAPINMOTIFBN	0<br>0	1<br>1	0<br>1	0<br>0	0<br>0	Sequence found in 5' upstream region of napin (2S albumin) gene in *Brassica napus* . Interact with a protein present in crude nuclear extracts from developing *Brassica napus* seeds.
	PROLAMINBOXOSGLUB1	0<br>0	1<br>0	0<br>0	0<br>2	0<br>0	"Prolamine box" found in the rice GluB-1 gene promoter.
	PROXBBNNAPA	0<br>0	0<br>0	0<br>0	0<br>0	1<br>1	"prox B (proximal portion of B-box) found in napA gene of *Brassica napus*. Required for seed specific expression and ABA responsiveness.
	RYREPEATBNNAPA	1<br>1	0<br>0	0<br>0	0<br>0	0<br>0	"RY repeat" found in RY/G box (the complex containing the two RY repeats and the G-box) of napA gene in Brassica napus . Required for seed specific expression
	SEF1MOTIF	1<br>1	0<br>0	0<br>0	0<br>0	0<br>0	"SEF1 (soybean embryo factor 1)" binding motif. Sequence found in 5'-upstream region of soybean beta-conglicinin (7Sglobulin) gene.
	SEF3MOTIFGM	2<br>2	0<br>1	1<br>2	0<br>1	0<br>0	"SEF3 binding site". Soybean consensus sequence found in the 5' upstream region of beta-conglycinin (7S globulin) gene.
	SEF4MOTIFGM7S	10<br>10	3<br>4	0<br>0	0<br>0	8<br>10	"SEF4 binding site"; Soybean consensus sequence found in 5'upstream region of beta-conglycinin (7S globulin) gene (Gmg17.1).
	SP8BFIBSP8BIB	1<br>1	0<br>1	0<br>0	0<br>0	1<br>0	One of SPBF binding site (SP8b). Found in gSPO-B1 (sporamin) and gB-Amy (beta-amylase) gene.
	TATCCAOSAMY	0<br>0	0<br>0	1<br>1	0<br>1	1<br>1	"TATCCA" element found in alpha-amylase promoters of rice binding sites of OsMYBS1, OsMYBS2 and OsMYBS3 which mediate sugar and hormone regulation of alpha-amylase gene expression.
	TATCCAYMOTIFOSRAMY3D	0<br>0	0<br>0	0<br>0	0<br>1	0<br>0	"TATCCAY motif" found in rice RAmy3D alpha-amylase gene promoter.

Supplementary Table S5. Continued

Category	*Cis*-elements	Genes					Description
		RPA1A	*RPA1C*	*RPA1E*	*RPA1B*	*RPA1D*	
Seed development and germination related Seed related	TGACGTVMAMY	1<br>1	2<br>2	0<br>1	0<br>0	0<br>0	"TGACGT motif" found in the Vigna mungo alpha-Amylase (Amy) gene promoter. Required for high level expression of alpha-Amylase in the cotyledons of the germinated seeds.
	WBBOXPCWRKY1	0<br>0	1<br>2	1<br>3	0<br>1	3<br>3	"WB box". WRKY proteins bind specifically to the DNA sequence motif (T)(T)TGAC(C/T), which is known as the W box. Found in amylase gene in sweet potato, alpha-Amy2 genes in wheat, barley, and wild oat, PR1 gene in parsley, and a transcription factor gene in Arabidopsis .
	WBOXHVISO1	1<br>1	1<br>6	2<br>4	0<br>4	5<br>8	SUSIBA2 bind to W-box element in barley iso1 (encoding isoamylase1) promoter.
Total number of *cis*-elements		33<br>33	25<br>47	10<br>46	1<br>34	28<br>38	
Abiotic stress related	ABRELATERD1	0<br>0	1<br>1	2<br>2	0<br>0	2<br>2	ABRE-like sequence required for etiolation-induced expression of erd1 (early responsive to dehydration) in Arabidopsis .
	ABRERATCAL	0<br>0	0<br>0	0<br>1	0<br>0	1<br>1	"ABRE-related sequence" or "Repeated sequence motifs" identified in the upstream regions of 162 Ca(2+)-responsive upregulated genes.
	ACGTABREMOTIFA2OSEM	0<br>0	0<br>0	1<br>1	0<br>0	0<br>0	Experimentally determined sequence requirement of ACGT-core of motif A in ABRE of the rice gene, OSEM. DRE and ABRE are interdependent in the ABA-responsive expression of the rd29A in Arabidopsis .
	ACGTATERD1	6<br>6	12<br>14	4<br>8	0<br>0	6<br>8	ACGT sequence required for etiolation-induced expression of erd1 (early responsive to dehydration) in A. thaliana.
	CBFHV	3<br>3	1<br>2	0<br>1	0<br>1	0<br>0	Binding site of barley CBF1, and also of barley CBF2. CBF= C-repeat (CRT) binding factors. CBFs are also known as dehydration-responsive element (DRE) binding proteins (DREBs).

Supplementary Table S5. Continued

Category	*Cis*-elements	Genes					Description
		RPA1A	*RPA1C*	*RPA1E*	*RPA1B*	*RPA1D*	
Abiotic stress related	CCAATBOX1	6	2	0	0	2	"CCAAT box" found in the promoter of heat shock protein genes. "CCAAT box" act cooperatively with HSEs to increase the hs promoter activity.
		6	4	1	2	3	
	CRTDREHVCBF2	2	0	0	0	0	Preferred sequence for AP2 transcriptional activator HvCBF2 of barley. DNA binding is regulated by temperature.
		2	0	0	0	0	
	CURECORECR	4	2	2	0	6	GTAC is the core of a CuRE (copper-response element) found in Cyc6 and Cpx1 genes in Chlamydomonas. Also involved in oxygen-response (Oxygene deficiency) of these genes.
		4	8	6	0	10	
	DPBFCOREDCDC3	1	0	2	1	1	A novel class of bZIP transcription factors, DPBF-1 and 2 (Dc3 promoter-binding factor-1 and 2) binding core sequence. Related ABA response.
		1	2	4	3	2	
	DRE1COREZMRAB17	0	0	0	0	0	"DRE1" core found in maize rab17 gene promoter. Related to ABA and drought response.
		0	1	0	0	0	
	DRE2COREZMRAB17	1	1	0	0	0	"DRE2" core found in maize (rab17 gene promoter. Related to ABA and drought response.
		1	1	1	0	0	
	DRECRTCOREAT	1	1	0	0	0	Core motif of DRE/CRT (dehydration-responsive element/C-repeat) *cis*-acting element found in many genes in A. thaliana and in rice.
		1	1	1	0	0	
	LTRE1HVBLT49	0	0	1	0	0	"LTRE-1" (low-temperature-responsive element) in barley blt4.9 gene promoter
		0	0	2	0	0	
	LTRECOREATCOR15	1	2	0	0	0	Core of low temperature responsive element (LTRE) of cor15a gene in Arabidopsis .
		1	2	1	0	0	
	MYB2AT	1	0	0	0	2	Binding site for ATMYB2. ATMYB2 is involved in regulation of genes that are responsive to water stress in Arabidopsis .
		1	0	0	0	1	
	MYBATRD22	1	0	0	0	0	Binding site for MYB (*At* MYB2) in dehydration-responsive gene, rd22.
		1	0	0	1	0	

Supplementary Table S5. Continued

Category	*Cis*-elements	Genes					Description
		RPA1A	*RPA1C*	*RPA1E*	*RPA1B*	*RPA1D*	
Abiotic stress related	MYBCORE	1<br>1	0<br>4	0<br>3	0<br>0	0<br>3	Binding site for all animal MYB and at least two plant MYB proteins *At* MYB1 and *At* MYB2, both isolated from Arabidopsis . *At* MYB2 is involved in regulation of genes that are responsive to water stress in Arabidopsis .
	MYCATERD1	2<br>2	0<br>0	0<br>22	0<br>0	0<br>1	MYC recognition sequence necessary for expression of erd1 (early responsive to dehydration) in dehydrated Arabidopsis.
	MYCATRD22	1<br>1	0<br>0	0<br>1	0<br>0	0<br>1	Binding site for MYC (rd22BP1) in Arabidopsis dehydration-resposive gene, rd22.
	MYCCONSENSUSAT	12<br>12	0<br>14	4<br>20	0<br>20	0<br>10	MYC recognition site found in the promoters of the dehydration-responsive gene rd22 and many other genes in Arabidopsis .
	PREATPRODH	0<br>0	1<br>1	0<br>0	0<br>0	0<br>0	"PRE (Pro- or hypoosmolarity-responsive element) found in the promoter region of proline dehydrogenase (ProDH) gene in Arabidopsis .
	SBOXATRBCS	0<br>0	0<br>1	0<br>0	0<br>0	0<br>0	"S-box" conserved in several rbcS promoters in *A. thaliana*. ABI4 binding site. "Important for the sugar and ABA responsiveness of CMA5.
	UPRMOTIFIAT	0<br>0	1<br>1	0<br>0	0<br>0	0<br>0	"Motif I" in the conserved UPR (unfolded protein response)cis-acting element in A. thaliana genes coding for SAR1B, HSP-90, SBR-like, Ca-ATPase 4, CNX1, PDI, etc.
Total number of cis-elements		43<br>43	24<br>57	16<br>75	1<br>27	20<br>42	
Biotic stress related	AGMOTIFNTMYB2	1<br>1	0<br>0	0<br>0	0<br>1	0<br>0	AG-motif found in the promoter of *Nt* Myb2 gene. *Nt* Myb2 is a regulator of the tobacco retrotransposon Tto1 and the defense-related gene phenylalanine ammonia lyase (PAL), which are induced by various stress such as wounding or elicitor treatment.
	BIHD1OS	2<br>2	0<br>4	2<br>3	2<br>6	2<br>3	Binding site of OsBIHD1, a rice BELL homeodomain transcription factor in disease resistance responses.

Supplementary Table S5. Continued

Category	*Cis*-elements	Genes					Description
		RPA1A	*RPA1C*	*RPA1E*	*RPA1B*	*RPA1D*	
**Biotic stress related**	BOXLCOREDCPAL	3	0	0	0	1	Consensus of the putative "core" sequences of box-L-like sequences in carrot By binding to this motif DcMYB1 acts as a transcriptional activator of DcPAL1 in response to Elicitor, UV-B and dilution treatment.
		3	0	0	0	1	
	ELRECOREPCRP1	0	0	0	0	1	ElRE (Elicitor Responsive Element) core of parsley (P.c.) PR1 genes. Consensus sequence of elements W1 and W2 of parsley PR1-1 and PR1-2 promoters.
		0	1	5	0	1	
	GCCCORE	0	0	0	0	1	Core of GCC-box found in many pathogen-responsive genes such as PDF1.2, Thi2.1, and PR4.
		0	0	0	0	1	
	GT1GMSCAM4	6	2	2	2	5	"GT-1 motif" found in the promoter of soybean (Glycine max) CaM isoform, SCaM-4. Plays a role in pathogen- and salt-induced SCaM-4 gene expression.
		6	2	7	5	9	
	HEXAT	0	1	0	0	0	"Hex motif". Binding site of Arabidopsis bZIP protein TGA1 and G box binding factor GBF1.
		0	1	0	0	0	
	MYB1LEPR	0	1	0	0	0	Tomato Pti4(ERF) regulates defence-related gene expression via GCC box and non-GCC box cis elements (Myb1(GTTAGTT)and G box (CACGTG).
		0	1	0	0	0	
	SEBFCONSSTPR10A	1	0	0	0	0	Binding site of the potato silencing element binding factor (SEBF) gene found in promoter of pathogenesis-related gene (PR-10a).
		1	1	0	2	0	
	T/GBOXATPIN2	0	0	0	0	0	"T/G-box" found in tomato proteinase inhibitor II (pin2) and leucine aminopeptidase (LAP) genes. Involved in jasmonate (JA) induction of these genes.
		0	0	0	0	1	
	TCA1MOTIF	0	0	1	0	0	TCA-1 (tobacco nuclear protein 1) binding site. Related to salicylic acid-inducible expression of many genes.
		0	1	1	1	0	
	WBOXATNPR1	2	2	3	2	8	"W-box" found in promoter of Arabidopsis NPR1 gene. They were recognized specifically by salicylic acid (SA)-induced WRKY DNA binding proteins.
		2	6	7	6	11	

Supplementary Table S5. Continued

Category	*Cis*-elements	Genes					Description
		RPA1A	*RPA1C*	*RPA1E*	*RPA1B*	*RPA1D*	
Biotic stress related	WBOXNTCHN48	0<br>0	0<br>1	1<br>1	1<br>3	0<br>0	"W box" identified in the tobacco class I basic chitinase gene CHN48. *Nt* WRKY1, *Nt* WRKY2 and *Nt* WRKY4 bound to W box. NtWRKYs possibly involved in elicitor-respsponsive transcription of defense genes in tobacco.
	WBOXNTERF3	1<br>1	1<br>7	2<br>5	1<br>6	6<br>9	"W box" found in the promoter region of a transcriptional repressor ERF3 gene in tobacco. May be involved in activation of ERF3 gene by wounding.
Total number of *cis*-elements		16<br>16	7<br>25	11<br>29	8<br>30	24<br>36	
Light related	AT1BOX	1<br>1	0<br>0	0<br>0	0<br>0	0<br>0	"AT-1 box (AT-rich element)" found in the promoter region of the genes for tobacco chlorophyll a/b binding protein (cab) and small subunit of ribulose-1,5-bisphosphate carboxylase (bcS).
	BOXCPSAS1	0<br>0	1<br>1	0<br>0	0<br>0	0<br>3	Box C in pea asparagine synthetase (AS1) gene. AS1 is negatively regulated by light.
	BOXIINTPATPB	2<br>2	0<br>0	0<br>2	0<br>2	2<br>2	"Box II" found in the tobacco atpB gene promoter. Conserved in several NCII (nonconsensus type II) promoters of plastid genes.
	BOXIIPCCHS	0<br>0	0<br>0	1<br>1	0<br>0	0<br>0	Core of "Box II/G box" found in the parsley chs genes. Essential for light regulation.
	CCA1ATLHCB1	3<br>3	1<br>1	0<br>0	1<br>2	1<br>3	CCA1 binding site. CCA1 protein (myb-related transcription factor) interact with two imperfect repeats of AAMAATCT in Lhcb1*3 gene of Arabidopsis Related to regulation by phytochrome.
	CDA1ATCAB2	0<br>0	0<br>0	0<br>0	0<br>1	0<br>0	CDA-1 (CAB2 DET1-associated factor 1) binding site in DtRE (dark response element) of chlorophyll a/b-binding protein2 (CAB2) gene in Arabidopsis .
	GATABOX	8<br>8	2<br>12	1<br>14	4<br>13	13<br>16	"GATA box"; Required for high level, light regulated, and tissue specific expression. Conserved in the promoter of all LHCII type I Cab genes.

Supplementary Table S5. Continued

Category	*Cis*-elements	Genes					Description
		RPA1A	*RPA1C*	*RPA1E*	*RPA1B*	*RPA1D*	
Light related	GT1CONSENSUS	13<br>13	9<br>18	6<br>20	4<br>16	16<br>22	Consensus GT-1 binding site in many light-regulated genes.
	GT1CORE	2<br>2	1<br>2	0<br>0	0<br>0	0<br>0	Critical for GT-1 binding to box II of rbcS.
	HDZIP2ATATHB2	1<br>1	0<br>0	0<br>0	0<br>0	0<br>0	Binding site of the A. thaliana homeobox gene (ATHB-2) found in its own promoter. ATHB-2 is regulated by light signals which function as a negative autoregulator of its own gene.
	IBOX	0<br>0	0<br>0	0<br>0	0<br>1	0<br>0	"I box"; Conserved sequence upstream of light-regulated genes.
	IBOXCORE	3<br>3	1<br>5	1<br>8	1<br>7	5<br>7	"I box"; Conserved sequence upstream of light-regulated genes.
	INRNTPSADB	6<br>6	3<br>7	2<br>6	0<br>5	4<br>4	"Inr (initiator)" elements found in the tobacco psaDb gene promoter without TATA boxes. Light-responsive transcription of psaDb depends on Inr, but not TATA box.
	LRENPCABE	0<br>0	0<br>0	1<br>1	0<br>0	0<br>0	"LRE"; A positive light regulatory element in tobacco CAB (cab-E) gene.
	PRECONSCRHSP70A	1<br>1	1<br>2	0<br>4	1<br>2	1<br>1	Consensus sequence of PRE (plastid response element) in the promoters of HSP70A in Chlamydomonas. Involved in induction of HSP70A gene by both MgProto and light.
	REALPHALGLHCB21	10<br>10	0<br>0	0<br>0	1<br>3	0<br>0	"REalpha" found in Lemna gibba Lhcb21 gene promoter. The DNA binding activity is high in etiolated plants but much lower in green plants; Required for phytochrome regulation.
	REBETALGLHCB21	0<br>0	0<br>0	0<br>0	0<br>1	0<br>0	"REbeta" found in Lemna gibba Lhcb21 gene promoter; Required for phytochrome regulation.
	RBCSCONSENSUS	1<br>1	1<br>1	0<br>0	0<br>0	0<br>0	rbcS (RuBisCO) general consensus sequence. Influences the level of gene expression and involved in light regulated gene expression.
	SORLREP3AT	0<br>0	0<br>0	0<br>0	1<br>1	0<br>0	One of sequences Over-Represented in Light-repressed Promoters (SORLREPs) in Arabidopsis Computationally identified phyA-repressed motifs.

Supplementary Table S5. Continued

Category	*Cis*-elements	Genes					Description
		RPA1A	*RPA1C*	*RPA1E*	*RPA1B*	*RPA1D*	
	SORLIP1AT	1	2	2	0	0	One of "Sequences Over-Represented in Light-Induced Promoters (SORLIPs) in Arabidopsis. Computationally identified phyA-induced motifs.
		1	1	3	0	0	
	SORLIP2AT	4	1	2	0	2	One of "Sequences Over-Represented in Light-Induced Promoters (SORLIPs) in Arabidopsis. Computationally identified phyA-induced motifs.
		4	1	2	0	2	
	SV40COREENHAN	1	0	0	0	0	"SV40 core enhancer"; similar sequences found in rbcS genes.
		1	0	0	0	0	
	TBOXATGAPB	1	3	0	0	2	"Tbox" found in the A. thaliana. GAPB gene promoter. Mutations in the "Tbox" resulted in reductions of light-activated gene transcription.
		1	3	1	1	4	
**Total number of *cis*-elements**		58	26	16	13	46	
		58	54	62	55	64	
**Hormone related Gibberellin**	CAREOSREP1	2	0	0	0	0	"CAREs (CAACTC regulatory elements)" found in the promoter region of a cystein proteinase (REP-1) gene in rice. CAREs are gibberellin responsive elements.
		2	0	0	0	0	
	CARGCW8GAT	2	4	0	0	4	A variant of CArG motif, with a longer A/T-rich core. Binding site for AGL15 (AGAMOUS-like 15). AGL15 regulates gibberellin metabolism.
		2	6	0	0	4	
	CARGNCAT	0	0	0	0	2	Noncanonical CArG motif (CC-Wx8-GG) found in the promoter region of DTA1. A relevant cis element for the response to AGL15. AGL15 regulates gibberellin metabolism.
		0	0	0	0	2	
	GARE2OSREP1	1	0	0	0	0	"Gibberellin-responsive element (GARE)" found in the promoter region of a cystein proteinase gene in rice.
		1	0	0	0	0	
	GAREAT	1	0	0	0	0	GARE (Gibberellin-responsive element).
		1	1	0	0	0	
	PYRIMIDINEBOXHVEPB1	0	0	0	0	1	"Pyrimidine box" found in the barley EPB-1 (cysteine proteinase) gene promoter. Required for gibberellic acid induction.
		0	1	1	0	1	

Supplementary Table S5. Continued

Category	*Cis*-elements	Genes *RPA1A*	*RPA1C*	*RPA1E*	*RPA1B*	*RPA1D*	Description
	PYRIMIDINEBOXOSRAMY1A	4<br>4	0<br>0	1<br>3	0<br>0	0<br>1	Pyrimidine box found in rice alpha-amylase (RAmy1A) gene. Gibberellin-respons *cis*-element of GARE and pyrimidine box are partially involved in sugar repression.
	WRKY71OS	6<br>6	4<br>14	4<br>9	3<br>14	9<br>13	"A core of TGAC-containing W-box" of, e.g., Amy32b promoter. Binding site of rice WRKY71, a transcriptional repressor of the gibberellin signaling pathway.
**Total number of cis-elements**		16<br>16	8<br>22	5<br>13	3<br>14	16<br>21	
**Hormone related (auxin)**	ARFAT	1<br>1	0<br>2	0<br>0	0<br>2	0<br>0	ARF (auxin response factor) binding site found in the promoters of primary/early auxin response genes of Arabidopsis.
	AUXRETGA1GMGH3	1<br>1	0<br>0	0<br>0	0<br>0	0<br>0	"TGA-box #1" in putative auxin-resonsive element (AUXRE) of soybean GH3 promoter.
	CACGCAATGMGH3	0<br>2	0<br>0	0<br>0	0<br>2	1<br>2	Sequence found in D4 element in Soybean GH3 gene promoter. Confers auxin inducibility.
	CATATGGMSAUR	2<br>2	0<br>0	0<br>0	0<br>2	2<br>2	Sequence found in NDE element in soybean SAUR (Small Auxin-Up RNA) 15A gene promoter; Involved in auxin responsiveness.
	NTBBF1ARROLB	2<br>2	0<br>0	1<br>3	0<br>1	0<br>1	NtBBF1binding site in Agrobacterium rhizogenes rolB gene. Required for tissue-specific expression and auxin induction.
**Total number of *cis*-elements**		6<br>6	0<br>2	1<br>3	0<br>5	3<br>4	
**Hormone related (cytokinine)**	ARR1AT	29<br>29	14<br>26	11<br>27	4<br>22	18<br>29	"ARR1-binding element" found in Arabidopsis. ARR1 is a response Regulator and involved in cytokinin response.
	CPBCSPOR	2<br>2	2<br>2	0<br>1	1<br>1	0<br>1	Critical for Cytokinin-enhanced Protein Binding in vitro; found in the promoter of the cucumber POR (NADPH-protochlorophyllide reductase) gene.
**Total number of *cis*-elements**		31<br>31	16<br>28	11<br>28	5<br>23	18<br>30	

Supplementary Table S5. Continued

Category	*Cis*-elements	Genes					Description
		RPA1A	*RPA1C*	*RPA1E*	*RPA1B*	*RPA1D*	
Growth and development related	ACGTTBOX	0	0	0	0	2	"T-Box" Motif, related to development
		0	2	0	0	2	
	ASF1MOTIFCAMV	3	3	0	0	1	Found in many promoters and are involved in transcriptional activation of several genes by auxin and/or salicylic acid; May be relevant to light regulation, growth and development.
		3	3	1	2	1	
	ATHB2ATCONSENSUS	0	0	0	0	0	Recognition sequence of Arabiddopsis Athb-2 protein. Growth and development
		0	0	4	0	0	
	DOFCOREZM	27	13	9	9	18	Core site required for binding of Dof proteins in maize.
		27	26	31	33	30	
	MARTBOX	4	0	2	0	2	"T-Box"; Motif found in SAR (scaffold attachment region; or matrix attachment region, MAR). related to development.
		4	0	4	0	6	
	MYB1AT	6	0	0	2	2	MYB recognition site found in the promoters of the dehydration-responsive gene rd22 and many other genes in Arabidopsis.
		6	3	0	5	4	
	MYB2CONSENSUSAT	1	2	0	0	4	MYB recognition site found in the promoters of the dehydration-responsive gene rd22 and many other genes in Arabidopsis.
		1	2	0	0	3	
	MYBPLANT	3	0	0	0	0	Plant MYB binding site; Consensus sequence related to box P in promoters of phenylpropanoid biosynthetic genes such as PAL, CHS,CHI, DFR, CL, Bz1.The AmMYB308 and AmMYB330 transcription factors from Antirrhinum majus regulate phenylpropanoid and lignin biosynthesis in transgenic tobacco.
		3	0	0	1	0	
	MYBST1	0	0	1	0	0	Core motif of MybSt1 (a potato MYB homolog) binding site
		0	1	2	4	2	
	PALBOXAPC	0	0	0	0	1	Box A; Consensus; One of three putative cis-acting elements (boxes P, A, and L) of phenylalanine ammonia-lyase. These elements appear to be necessary but not sufficient for elicitor-or light-mediated PAL gene activation.
		0	0	0	0	1	

Supplementary Table S5. Continued

Category	*Cis*-elements	Genes					Description
		RPA1A	*RPA1C*	*RPA1E*	*RPA1B*	*RPA1D*	
	QARBNEXTA	0<br>0	0<br>0	0<br>0	0<br>0	1<br>1	"QAR (quantitative activator region)" in promoter region of Brassica napus extA extensin gene. Extensins are a family of flexuous, rodlike, hydroxyproline-rich glycoproteins (HRGPs) of the plant cell wall.
	RAV1AAT	11<br>11	0<br>4	1<br>4	1<br>4	4<br>6	Binding consensus sequence of Arabidopsis transcription factor, RAV1.
	RAV1BAT	0<br>0	1<br>1	0<br>0	1<br>1	0<br>0	Binding consensus sequence of an A. thaliana transcription factor, RAV1.
	XYLAT	0<br>0	0<br>0	1<br>1	0<br>0	0<br>0	Cis-element identified among the promoters of the "core xylem gene set".
**Total number of cis-elements**		55<br>55	19<br>42	14<br>47	13<br>50	35<br>56	
**Nodule related**	NODCON1GM	1<br>1	3<br>5	1<br>5	2<br>4	3<br>3	One of two putative nodulin consensus sequences; See also NODCON2GM
	NODCON2GM	12<br>12	1<br>3	0<br>4	3<br>7	3<br>7	One of two putative nodulin consensus sequences; See also NODCON1GM.
	OSE1ROOTNODULE	1<br>1	3<br>5	1<br>5	2<br>4	3<br>3	One of the consensus sequence motifs of organ-specific elements (OSE) characteristic of the promoters activated in infected cells of root nodules. See also OSE2ROOTNODULE
	OSE2ROOTNODULE	12<br>12	1<br>3	0<br>4	3<br>8	3<br>7	One of the consensus sequence motifs of organ-specific elements (OSE) characteristic of the promoters activated in infected cells of root nodules. See also OSE1ROOTNODULE.
**Total number of cis-elements**		26<br>26	8<br>16	2<br>18	10<br>23	12<br>20	
**Fermentative pathway related**	ANAERO1CONSENSUS	4<br>4	1<br>2	1<br>3	1<br>3	6<br>6	One of 16 motifs found in silico in promoters of 13 anaerobic genes involved in the fermentative pathway
	ANAERO2CONSENSUS	0<br>0	0<br>0	0<br>0	1<br>1	0<br>0	One of 16 motifs found in silico in promoters of 13 anaerobic genes involved in the fermentative pathway.
	ANAERO3CONSENSUS	0<br>0	0<br>0	0<br>0	1<br>1	0<br>0	One of 16 motifs found in silico in promoters of 13 anaerobic genes involved in the fermentative pathway.

Supplementary Table S5. Continued

Category	*Cis*-elements	Genes					Description
		RPA1A	*RPA1C*	*RPA1E*	*RPA1B*	*RPA1D*	
	ANAERO4CO<br>NSENSUS	0<br>0	1<br>1	0<br>0	0<br>0	0<br>0	One of 16 motifs found in silico in promoters of 13 anaerobic genes involved in the fermentative pathway.
**Total number of *cis*-elements**		4<br>4	2<br>3	1<br>3	3<br>5	6<br>6	
**Axillary bud outgrowth related**	SREATMSD	0<br>0	0<br>1	1<br>1	0<br>3	1<br>1	"sugar-repressive element (SRE)" found in 272 of the 1592 down-regulated genes after main stem decapitation in Arabidopsis. It might regulate expression of some genes during initiation of axillary bud outgrowth in Arabidopsis.
	UP1ATMSD	1<br>1	0<br>0	2<br>2	0<br>0	1<br>1	"Up1" motif found in 162 of the 1184 up-regulated genes after main stem decapitation in A. thaliana. It might regulate expression of some genes during initiation of axillary bud outgrowth in Arabidopsis.
	UP2ATMSD	1<br>1	0<br>0	0<br>0	0<br>0	0<br>0	"Up2" motif found in 193 of the 1184 up-regulated genes after main stem decapitation in Arabidopsis. It might regulate expression of some genes during initiation of axillary bud outgrowth in Arabidopsis.
**Total number of *cis*-elements**		2<br>2	0<br>1	3<br>3	0<br>3	2<br>2	
**Promoter related**	-10PEHVPSBD	3<br>3	0<br>0	0<br>1	1<br>1	0<br>0	-10 promoter element
	CAATBOX1	25<br>25	8<br>21	8<br>32	1<br>19	19<br>20	"CAAT promoter consensus sequence" found in legA gene of pea. KW CAAT; legA; seed.
	TATABOX2	0<br>0	2<br>2	0<br>1	0<br>0	3<br>4	"TATA box"; TATA box found in the 5'upstream region of pea legA gene and sporamin A of sweet potato.
	TATABOX3	3<br>3	1<br>0	0<br>0	0<br>0	1<br>0	"TATA box"; TATA box found in the 5'upstream region of sweet potato sporamin A gene.
	TATABOX4	3<br>3	6<br>1	1<br>0	0<br>0	2<br>4	"TATA box"; TATA box found in the 5'upstream region of sweet potato sporamin A gene.
	TATABOX5	1	5	2	1	5	"TATA box"; TATA box found in the 5'upstream region of pea glutamine synthetase gene.

Supplementary Table S5. Continued

Category	*Cis*-elements	Genes					Description
		RPA1A	*RPA1C*	*RPA1E*	*RPA1B*	*RPA1D*	
	TATAPVTRNALEU	0	1	0	0	2	"TATA-like motif"; A TATA-like sequence found in Phaseolus vulgaris tRNALeu gene promoter.
		0	1	0	0	3	
	TATABOXOSPAL	1	0	0	0	1	Binding site for *Os* TBP2, found in the promoter of rice pal gene encoding phenylalanine ammonia-lyase.
		1	1	0	0	1	
**Total number of *cis*-elements**		36	18	11	3	32	
		36	31	36	21	37	
**Other *cis*-elements**	BS1EGCCR	0	0	0	0	1	"BS1 (binding site 1)" found in E. gunnii Cinnamoyl-CoA reductase(CCR) gene promoter. nuclear protein binding site; Required for vascular expression.
		0	0	0	0	1	
	CGCGBOXAT	0	0	0	2	0	"CGCG box" recognized by AtSR1-6 (A. thaliana signal-responsive genes). Multiple CGCG elements are found in promoters of many genes. Ca++/calmodulin binds to all AtSRs.
		0	0	2	2	0	
	CIACADIANLELHC	4	0	1	0	1	Region necessary for circadian expression of tomato (L.e.) Lhc gene.
		4	1	1	4	1	
	CACTFTPPCA1	13	8	7	1	11	Tetranucleotide (CACT) is a key component of Mem1 (mesophyll expression module 1) found in the cis-regulatory element in the distal region of the phosphoenolpyruvate carboxylase (ppcA1) of the C4 dicot F. trinervia.
		13	22	22	13	26	
	EECCRCAH1	2	1	2	1	3	"EEC"; Consensus motif of the two enhancer elements, EE-1 and EE-2, both found in the promoter region of the Chlamydomonas Cah1<br>Binding site of Myb transcription factor LCR1
		2	3	5	4	4	
	HEXAMERATH4	0	0	0	0	0	hexamer motif of Arabidopsis histone H4 promoter.
		0	0	1	0	0	
	HEXMOTIFTAH3H4	1	2	0	0	0	"hexamer motif" found in promoter of wheat histone H3 and H4."hexamer motif" in type 1 element may play important roles in regulation of replication- dependent but not of replication-independent expression of the wheat histone H3 gene.
		1	2	1	0	0	
		1	2	1	0	0	

Supplementary Table S5. Continued

Category	*Cis*-elements	Genes					Description
		RPA1A	*RPA1C*	*RPA1E*	*RPA1B*	*RPA1D*	
Other *cis*-elements	L1BOXATPDF1	0	0	1	0	0	"L1 box" found in promoter of A. thaliana ROTODERMAL FACTOR1 (PDF1) gene. Involved in L1 layer-specific expression.
		0	0	1	0	0	
	MYBPZM	3	0	0	1	0	Core of consensus maize P (myb homolog) binding site. Maize P gene specifies red pigmentation of kernel pericarp, cob, and other floral organs.
		3	0	0	3	0	
	RHERPATEXPA 7	0	0	0	0	1	"Right part of RHEs (Root Hair-specific *cis*-elements)" conserved among the A. thaliana A7 (*At* EXPA7) homologous genes from diverse angiosperm species with different hair distribution patterns
		0	0	0	1	1	
	S1FBOXSORPS1 L21	1	1	0	0	1	"S1F box" conserved both in spinach RPS1 and RPL21 genes encoding the plastid ribosomal protein S1 and L21, respectively; Negative element; Might play a role in downregulating RPS1 and RPL21 promoter activity
		1	1	0	0	4	
	S1FSORPL21	0	1	0	0	0	"S1F binding site" ("S1 site") in spinach RPL21 gene encoding the plastid ribosomal protein L21; Negative element; Might play a role in down regulating RPL21 promoter activity.
		0	1	0	0	0	
	SITEIIATCYTC	2	1	2	0	2	"Site II element" found in the promoter regions of cytochrome genes (Cytc-1, Cytc-2) in Arabidopsis. Overrepresented in the promoters of nuclear genes encoding components of the oxidative phosphorylation (OxPhos) machinery from both Arabidopsis and rice
		2	1	3	0	2	
	SURE2STPAT21	0	0	0	0	1	Sucrose Responsive Element 2 (SURE2). A motif conserved among genes regulated by sucrose. Found in the patatin (a major tuber protein)gene promoter of potato.
		0	0	0	0	1	
	SURECOREATS ULTR11	2	0	2	0	5	Core of sulfur-responsive element (SURE) found in the promoter of SULTR1.1 high-affinity sulfate transporter gene in Arabidopsis.
		2	2	3	3	1	

Supplementary Table S5. Continued

Category	*Cis*-elements	Genes					Description
		RPA1A	*RPA1C*	*RPA1E*	*RPA1B*	*RPA1D*	
Other *cis*-elements	TAAAGSTKST1	5	2	3	2	0	TAAAG motif found in promoter of *Solanum tuberosum* (KST1 gene; Target site for trans-acting StDof1 protein controlling guard cell-specific gene expression; KST1 gene encodes a K+ influx channel of guard cells
		0	0	0	0	1	
	POLASIG1	4	3	1	2	4	"PolyA signal"; poly A signal found in legA gene of pea, rice alpha-amylase.
		4	5	2	2	5	
	POLASIG2	5	2	3	0	2	"PolyA signal"; poly A signal found in rice alpha-amylase.
		5	3	0	0	2	
	POLASIG3	4	3	0	1	4	"Plant polyA signal"; Consensus sequence for plant polyadenylation signal.
		4	6	4	1	5	
	TRANSINITDICOTS	0	0	1	0	0	Context sequence of translational initiation codon in dicots.
		0	0	1	1	0	
	TRANSINITMONOCOTS	0	0	1	0	0	Context sequence of translational initiation codon in monocots
		0	0	1	1	0	
	INTRONLOWER	1	0	0	0	0	"3' intron-exon splice junctions"; Plant intron lower sequence. Consensus sequence for plant introns.
		1	0	0	2	0	
	INTRONUPPER	0	0	0	0	0	"5' exon-intron splice junctions" of plant introns.
		0	0	1	0	0	
