# Supplementary material for: Molecular Evolution and Functional Diversification of Replication Protein A1 in Plants
Source: Front Plant Sci. 2016 Jan 29;7:33. doi: 10.3389/fpls.2016.00033 (PMC4731521; doi:10.3389/fpls.2016.00033)
Supplement: Supplementary file 2 [file DataSheet1.PDF]

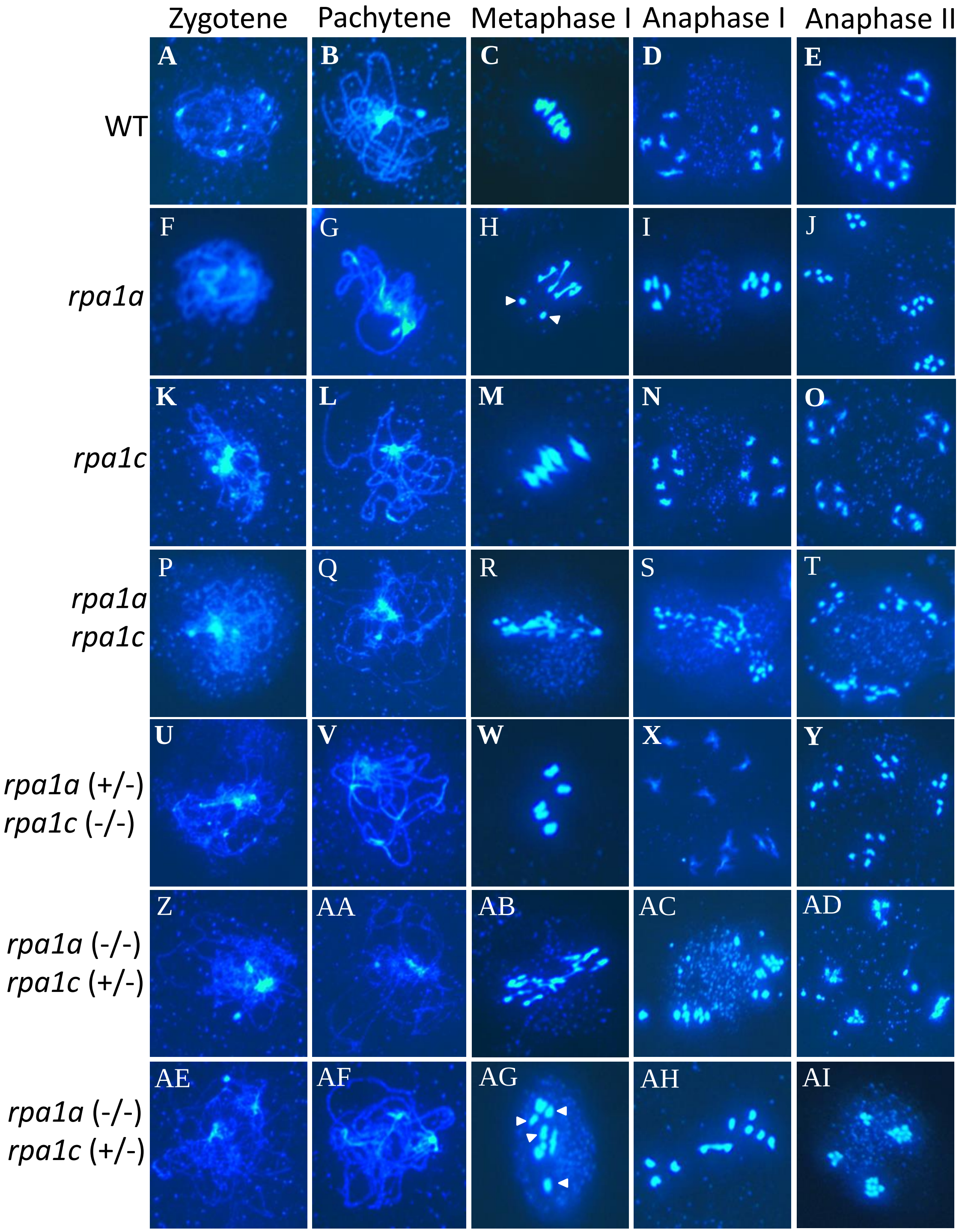

Supplementary Figure S1. Meiotic stages of pollen-mother cells from Arabidopsis WT and *rpa1* mutants. In WT, *rpa1c,* and *rpa1a* (+/-) *rpa1c* (-/-) homologs are segregated in equal number at anaphase I (D, N, X), followed by the separation and segregation of sister chromatids at anaphase II (E, O, Y). In *rpa1a*, zygotene (F) and pachytene (G) stages are similar to WT, *rpa1c* and *rpa1a* (+/-) *rpa1c* (-/-). However, univalents (arrows) are present at the metaphase plate (H) leading to unequal segregation of homologous chromosomes at anaphase I (I), and anaphase II (J). In *rpa1a rpa1c* , multiple fragmented chromosomes were present at metaphase plate (R) and at anaphase I (S) leading to abnormal anaphase II (T). In *rpa1a* (-/-) *rpa1c* (+/-), a combination of fragmented (AB) and univalent (AG) chromosomes were present at metaphase plate leading abnormal anaphase I and II with broken chromosomes (AC, AD) and unequal segregation (AH, AI).

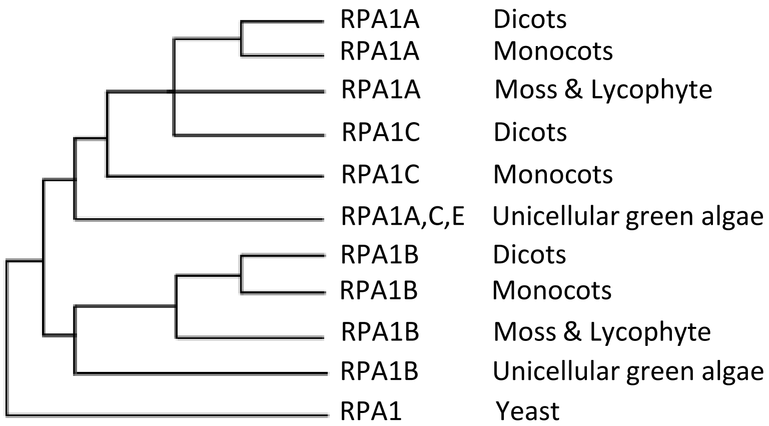

Supplementary Figure S2. Summary of evolutionary relationships of RPA1 proteins. The tree is constructed based on original tree that is constructed using the Maximum-Likelihood method performed with MEGA5.2 software package (Figure 2). The Baker's yeast (*S. cerevisiae*) RPA1 is used as an out-group to root the tree.

A

```
                                                        #  #          #  #                  #
RPA1  H.sapiens               8 GAIAAI.[10].ILQVINIRPI.[ 7].RYRLLMSDG.[32].FIVNTLK.[2].RRVVILMELEVLK  103
RPA1  X.tropicalis            9 GAISAI.[ 9].TLQVINIRSI.[ 7].RYRLLMSDG.[32].FIVNNLK.[2].RRVIIVMEMDVLK  103
RPA1  D.rerio                 8 GAIESL.[ 9].ILQCVNIRKI.[ 7].RFRVMMSDG.[32].SVTNVLK.[2].RRVVVILDIEVLK  102
RPA1  D.melanogaster          9 GVIARI.[ 9].VLQILAIKKI.[ 7].RYRILISDG.[32].YVTSLVG.[5].RRVLIISELTVVN  106
RPA1  S.cerevisiae            9 GDFHSI.[13].VYQVYNTRKS.[ 7].KNLIMISDG.[31].AEPAIVR.[3].KYVLLVDDFELVQ  107
RPA1  S.pombe                 8 GALR-I.[13].ILQVLTVKEL.[10].RYRVVLSDS.[31].FTVNVMK.[1].RKILIVLGLNVLT  107
RPA1  N.crassa                8 GALDAM.[13].ILQCLQIKTL.[11].RFRIVLSDL.[31].YQAQCLK.[1].KNILIVLDLEVIQ  108
RPA1  U.maydis                9 GAIAQM.[14].VCQILSIKKI.[12].RYRIILSDG.[31].FASNSVQ.[1].RRILILLDLDVVH  111
```

B

```
                                                         #  #          #  #                 #
RPA1     H.sapiens            9 AIAAIM.[7].KPILQVINIRPI.[ 7].RYRLLMSDG.[28].QIHRFI.[7].RRVVILMELEVLK  103
RPA1A    A.thaliana           9 AITAIH.[6].KPLLQVLEIKMI.[ 9].RYRFLISDG.[27].QLIDYI.[6].RKLIVVLNMETIV  102
RPA1A    A.lyrata             9 AITAIN.[6].KPLLQVLEIKMI.[ 9].RYRFLISDG.[27].QLIDYI.[6].RKLIVVLNMETIV  102
RPA1A    C.rubella            9 AITAIN.[6].KPLLQVLDIKMI.[ 9].RYRFLISDG.[27].QLIDYI.[6].RKVIVVLNMETLV  102
RPA1A    Tomato               9 AISAIN.[6].KPVVQVLDIKLI.[ 5].RYRLTLSDS.[27].QLIEYI.[6].RKIIVVLNMETII   98
RPA1A    Strawberry           9 AVPIIN.[6].KPLMQVTDVKLI.[ 4].RYRLLLSDG.[27].QLTEYI.[6].RKIIVALNMETIV   97
RPA1A    Cucumber             9 AISAIV.[6].KPLVQVLDIKLI.[ 5].RYSLLISDA.[27].QLIDYV.[6].RKIIVVLCLETII   98
RPA1A    Soybean              9 AIPAII.[6].KPLVQVLDVALV.[ 9].RYRLLLSDA.[27].QLLDYI.[6].RKIIVILNMETIM  102
RPA1A    Barel clover         9 AIPAIT.[6].KPLVQVISITLL.[11].KYYLKLSDG.[27].KLLDYV.[6].RKIIIVHKMETIV  104
RPA1A    Castor oil plant     9 AISMIN.[6].KPLVQVVDIKQI.[ 5].RYRFLISDS.[27].QLIDYI.[6].RKIIVVLNLETII   98
RPA1A    Grape                9 AIAAIN.[6].KPLVQVLDIKLI.[ 5].RYRFLLSDA.[27].QLIDYI.[6].RKIIVVLNMETII   98
RPA1A    Cacao                9 AIASIN.[6].KPLVQVVDIKLI.[ 5].RYRFLLSDS.[27].QLIDYV.[6].RRIIVVLNMETII   98
RPA1A    California popular   9 AIAMIN.[6].KPLVQVLDIKQM.[ 4].RFRLLVSDS.[27].QLIDYI.[6].RKIIVVLNLETII   97
RPA1A    Peach               10 AIAAIS.[6].KPLVQVIDIKLL.[ 5].RYRFIVSDA.[27].QLTDYI.[6].HKIIVVLNMETII   98
RPA1A    Maize               10 AVAAAM.[6].KPVVQVVDLRSI.[10].RFRAIISDG.[27].QLLDYI.[6].RKAIVILNMEVLV  104
RPA1A    Sorghum             10 AVAAAM.[6].KPVVQVVDLRSI.[ 8].RFRAIISDG.[27].QLLDYI.[6].RKAIVILNLEVLA  105
RPA1A    Millet              10 AVTATL.[6].KPVVQVVDLRSI.[10].RFRAIISDG.[27].QLLDYI.[6].RRAMVILNMEVLV  107
RPA1A    Rice                10 GVAAAL.[6].KPVLQIVELRGV.[11].RFRAVVSDG.[27].QLSEYV.[6].RRIIVILNLEVLV  105
RPA1A-1  B.distachyon        10 AVEAVL.[6].RPIVQVLDVRCV.[ 5].RWRGNVSDG.[27].QLDEYV.[6].RRIIVVLNMTVLR   99
RPA1A-2  B.distachyon        10 AVAAVL.[6].RPVVQVVDLRRA.[ 3].RWRACVSDG.[27].QLDDYV.[6].RRIVVVLNMTVLR   99
RPA1A-1  S.moellendorffii    10 AIVALL.[6].RPIVQVLDVKQI.[ 8].RYRLVLSDG.[27].KLQEYI.[6].RKIVIVLNLDVLA  102
RPA1A-2  S.moellendorffii    10 AIVALL.[6].RPIVQVLDVKQI.[ 8].RYRLVLSDG.[27].KLQEYI.[6].RKIVIVLNLDVLV  102
RPA1A-1  P.patens             9 AIVALN.[6].RPVLQIVDVRQI.[ 8].RFRLVLSDG.[27].QLLEYI.[6].RKIIIVLNMEIVE  101
RPA1A-2  P.patens             9 AIVALN.[6].RPVLQIVDIRQI.[ 8].RFRLVLSDG.[27].QLLEYI.[6].RKIIIVLNMEIVE  101
```

C

```
                                                         #  #          #  #                 #
RPA1     H.sapiens            5 LSEGAI.[12].PILQVINIRPI.[ 7].RYRLLMSDG.[27].VCQIHR.[9].RRVVILMELEVLK  104
RPA1C    A.thaliana           5 LTEGVV.[15].PVLQVTELKLI.[12].RYKFLLSDG.[26].VIRLTH.[8].RRIVVIMQLEVIV  110
RPA1C    A.lyrata             5 LTAGAI.[15].PVLQVTELKMI.[ 3].RYKFLLSDG.[26].IVRLTH.[8].RRIVIIMQLEVIV  101
RPA1C    C.rubella            5 LTTGAI.[15].PVLQVTELKLI.[11].RYKVSLSDG.[26].ILRVTQ.[8].RRIVIISNLEIIV  109
RPA1C    Tomato               6 LTEGAV.[14].PVLQITDVRLV.[10].RYRLLLSDG.[26].IIQMNE.[8].RVIIIIIELDILV  108
RPA1C    Strawberry           5 LTEGAV.[13].PVLQVTDLRQI.[10].RYRLNLSDG.[26].IVCLTE.[8].RRIIIIVSLDVLV  106
RPA1C    Cucumber            10 LTEGAV.[14].PILQVIDLKLV.[ 9].RFRLLVSDG.[26].IVRLRQ.[8].RLIIIVIELDVIE  111
RPA1C    Soybean              5 LTQSAI.[12].PVLQVIELKLV.[ 9].RYRLVLSDG.[26].VVRLTQ.[8].RKIIIIVDLDVVL  105
RPA1C    Barel clover         5 LTQGAI.[10].PVLQVIDLKLV.[ 9].RYRVVLSDG.[26].IVKLSQ.[8].RKIIIIVDLDVIM  103
RPA1C    Castor oil plant     6 LSEGAI.[14].PTLQVTELKQV.[ 9].RFRLILSDG.[26].VVQLIQ.[8].RMLLGSRSCIGCE  107
RPA1C    Grape                5 LTEGAI.[14].PVLQVADIRLV.[10].RFRILLSDG.[26].VVQLTQ.[8].RMIIIIIDLDVIL  107
RPA1C    Cacao                5 LTGGAV.[14].PVLQVIELKEV.[18].RFRLLLSDG.[26].IVQLTQ.[8].RMIVIIIELEVII  116
RPA1C    California popular   3 LTEGAI.[14].PTLQVTDLKQV.[ 9].RFRLVLSDG.[26].IVQLNQ.[8].RMIIIILELALVA  104
RPA1C    Peach                5 LTEGAI.[14].PILQVLDVRMV.[14].RYRVLISDG.[26].IVCLKQ.[8].----ALPGNAQSI  107
RPA1C    Maize                5 LTPGAV.[14].PVLQVVDVRMV.[ 9].RFRMVLSDG.[26].IVHLLE.[8].RRIIIVIKLDVLQ  106
RPA1C-1  Sorghum              5 LTTGAV.[14].PVLQVVDVRIV.[10].RFRMLLSDG.[26].IVHLHE.[8].RRIIIIIKLDVLQ  107
RPA1C-2  Sorghum              8 LTPGAV.[14].PVLQVLEVRRV.[20].RYRLILSDG.[26].IVHLNE.[8].KRIIIIVKIEILQ  120
RPA1C-4  Sorghum             47 LSTGAL.[10].PVLQLADAPQM.[ 9].RYRVALSDG.[27].VIRVLE.[8].QRIIIVIQLEILQ  145
RPA1C-1  Millet               7 LTPGGV.[13].PVLQVVDVRRV.[11].RYRMVLSDG.[26].IIHLQE.[8].RRIIIIIKLEVLQ  110
RPA1C-2  Millet               9 LSHGAV.[12].PVLQVADARHV.[12].RYRIDLSDG.[25].VVRVLD.[8].RRTITVIQLQILQ  110
RPA1C    Rice                 5 LTPGAV.[14].PVLQVVDVRPV.[13].RFRMMLSDG.[26].VVQLTD.[8].KRIIIVVKLDVLQ  110
RPA1C    B.distachyon         6 LTEGAV.[14].PVVQVGDVKIV.[11].RYRMLISDG.[26].IVHLVE.[8].RRIIIVTKLDVLQ  109
```

Supplementary Figure S3. Amino acid alignment of the basic cleft (binding surface I) domain found in DBD-F of RPA1 from various species. Hash marks show the position of the conserved residues. Numbers in parenthesis indicate the number of omitted amino acids. (A) Sequences from RPA1 of animals and fungi (Supplementary Table S3), (B) sequences from RPA1A of plants, (C) sequences from RPA1C and RPA1E of plants, (D, next page) sequences from RPA1B and RPA1D of plants. Sequences were aligned by ClustalW hosted in MEGA5.

Supplementary Figure S3. Continued

D

```
                                             #  #         #  #            #
RPA1     H.sapiens          5 LSEGAIAA.[13].QVINIRPI.[7].RYRLLMSDGL.[33].VNTLKD-GR      RVVILMELEVL  106
RPA1B    A.thaliana         5 VTQDGIAT.[18].QVVDLKPA.[2].RYTFSANDGK.[32].VNDIPGKSE.[1].KYMLITKCEAV  107
RPA1B    A.lyrata           5 VTQDGIAT.[18].QVVDLKPV.[2].RYTFNANDGN.[32].VNDIPGKSE.[1].KYMLITKCEAV  107
RPA1B    C.rubella          8 VTQDGIAT.[18].QVVDLKAV.[2].RYTFSANDGK.[32].VNDIPGKSE.[1].KYMLITKCEAV  110
RPA1B    Tomato             7 VTPDAIST.[18].QVVDLKPT.[2].RYMFSANDGK.[32].LNDIPTKNE      KYLIVTKCEAV  105
RPA1B    Strawberry         6 PTPDAIST.[18].QILDLRRN.[2].RYMFTASDGK.[32].VNDIPGKPE      KCMIVIKCEMV  106
RPA1B    Cucumber           5 PTAGGISK.[18].QVIDLKAT.[2].RYMFTASDGV.[32].LNDIPNKSE      KYLIVTKCEVV  105
RPA1B-1 Soybean             6 VTPDAVST.[18].QVLDLKAT.[2].KYMFTANDGK.[32].VNDIPNKSD      KYLLAIKCEAV  106
RPA1B-2 Soybean             6 VTPDAVSM.[18].QVLDLKAT.[2].KYMFTANDGK.[32].VNVIPNKSD      KYLIVTKCEPV  106
RPA1B    Castor oil plant   5 VSPDAIAA.[18].QITNLEPK.[2].SYGFDANDGK.[32].VNEIPSKSE      NYLIITKCEVV  105
RPA1B    Grape              5 ITPNGIST.[18].QVIDLTPI.[2].RYKFTANDGK.[32].LNDIPSKQE      KYLIVTKCEAV  105
RPA1B    Cacao             61 VTRDAIST.[18].QVLDLKLT.[2].RYTFNASDGN.[32].LNDIPNKSE      KYLIVKKCETV  161
RPA1B    California popular 9 VSPDGISK.[18].QVTNLEPK.[2].SYGFDASDGK.[32].VNEIPSKSE      KFLIITKCEVV  109
RPA1B    Peach              6 PTPDAIST.[18].QVLDLGPR.[2].TYKFTASDGK.[32].VNEIPGMSE      NYVLVKQCEVV  106
RPA1B-1 Maize               7 VTPGAVSY.[18].QVLDLKSI.[4].RFSFTATDGN.[32].CNSVKGNAD      KVLIVVKCETV  109
RPA1B-2 Maize               7 VTPVAVSH.[18].QVLDLKSV.[4].RFSFTATDGK.[32].CNVVKGKDD      KVLVVIKCELV  109
RPA1B    Sorghum            7 VTPGAVSH.[18].QVLDLKSI.[4].RFSFMATDGK.[32].CNAVNGNNG      KVLIVIKCELV  109
RPA1B    Millet             7 VTPGAVSH.[18].QVVDLKSI.[2].RFGFMASDGK.[31].CNAVGGNND.[1].KALIITKCEVV  108
RPA1B    Rice               9 VTPGAVAF.[20].QVVDLKPI.[2].RFTFLASDGK.[32].CNTIGEKQE      KVLIITKLEVV  111
RPA1B    B.distachyon       6 VTPDALAI.[18].QVVDLKPL.[4].RFTFMASDGK.[32].CNDIPKTLS      KCLIITKCEVV  108

RPA1D    A.thaliana         5 VTPDAIST.[18].QVVDLKPI.[2].RYTFSANDGK.[32].VNDISSKST      KYFLVTKCEAV  106
RPA1D    A.lyrata           5 VTPDAIST.[18].QVVDLKPI.[2].RYTFSANDGK.[32].VNDISSKST      KYFLVNKCEAV  106
RPA1D    C.rubella         54 VTAGAIST.[18].QVVDLKPI.[2].RYTFSANDGK.[32].VNDISSKAT      KYFLVTKCEAV  155
```

A

```
                                         ###          #### #     ### #               #       ###          ###
RPA1B    A. thaliana            26 PEIVVQVVDLKP.[3].RYTFSANDGKMKIKAMLPATLTSDIISGKIQNLGLIRLLEYTVNDIP.[4].EKYMLITKCEAV  103
RPA1B    A. lyrata              26 PEIVVQVVDLKP.[3].RYTFNANDGNMKMKAMLPATLTCEIISGKIQNLGLVRLLDYTVNDIP.[4].EKYMLITKCEAV  103
RPA1B    C. rubella             29 PQIVVQVVDLKA.[3].RYTFSANDGKTKIKAMLPASLTSDIISGKIQSLGLIRLLDYTVNDIP.[4].EKYMLITKCEAV  106
RPA1B    Tomato                 26 PEIIVQVVDLKP.[3].RYMFSANDGKMKIKGILQSSLSSEVISGSIQNLGLIRVIDYTLNDIP.[3].EKYLIVTKCEAV  102
RPA1B    Cucumber               26 PDVVVQVIDLKA.[3].RYMFTASDGVMKLKAILPSNLTSDVISGNIQNLGLIRILDYSLNDIP.[3].EKYLIVTKCEVV  102
RPA1B    Strawberry             27 PEIVVQILDLRR.[3].RYMFTASDGKMKLKGILNSDMSSMVDSGDFQNLSLVRILEYTVNDIP.[3].EKCMIVIKCEMV  103
RPA1B-1  Soybean                27 PDIVVQVLDLKA.[3].KYMFTANDGKTKLKAMIPSDMRSQVLSGAIQNLGLIRVLDYTVNDIP.[3].DKYLLAIKCEAV  103
RPA1B-2  Soybean                27 PEIVVQVLDLKA.[3].KYMFTANDGKTKLKAMISSDMCSQVLSGAIQNLGLIRVLDYTVNVIP.[3].DKYLIVTKCEPV  103
RPA1B    Castor oil plant       26 PEIIVQITNLEP.[3].SYGFDANDGKRKIKAIFNSRLSTEIISGNIQNLGLIRILDYTVNEIP.[3].ENYLIITKCEVV  102
RPA1B    Grape                  26 PEIIVQVIDLTP.[3].RYKFTANDGKMKLKAMFPSSFSSEINSGNIQNLGLIQVIDYTLNDIP.[3].EKYLIVTKCEAV  102
RPA1B    Cacao                  82 LEIVVQVLDLKL.[3].RYTFNASDGNMKLRAIFPSNVSSEITSGSIQNKGLVKILDYTLNDIP.[3].EKYLIVKKCETV  158
RPA1B    California popular     30 PEIILQVTNLEP.[3].SYGFDASDGKMKIKAIFSSRLSSEILSGNIQNLCLIRVLDYTVNEIP.[3].EKFLIITKCEVV  106
RPA1B    Peach                  27 PDIVIQVLDLGP.[3].TYKFTASDGKMKLKGMFSSQLASQITSGNIQNLGLVRILDYAVNEIP.[3].ENYVLVKQCEVV  103
RPA1B-1  Maize                  28 SDLVVQVLDLKS.[5].RFSFTATDGNDKIKAMLPTYFASEVHSGNLKNFGLIRILDYTCNSVK.[3].DKVLIVVKCETV  106
RPA1B-2  Maize                  28 TDLVVQVLDLKS.[5].RFSFTATDGKDKIKAMLPTNFGSEVRSGNLKNLGLIRIIDYTCNVVK.[3].DKVLVVIKCELV  106
RPA1B    Sorghum                28 PDLVVQVLDLKS.[5].RFSFMATDGKDKIKAMFPTSFASEVRSGNLKNLGLIRILDYTCNAVN.[3].GKVLIVIKCELV  106
RPA1B    Millet                 28 PELVVQVVDLKS.[3].RFGFMASDGKDKIKAMLPTQFAAEVRSGNLQNLGLIRILDYTCNAVG.[4].NKALIITKCEVV  105
RPA1B    Rice                   32 PEIVLQVVDLKP.[3].RFTFLASDGKDKIKTMLLTQLAPEVRSGNIQNLGVIRVLDYTCNTIG.[3].EKVLIITKLEVV  108
RPA1B    B. distachyon          27 PEIVVQVVDLKP.[6].RFTFMASDGKAKMKAMLPTNFASEVNSGNLQNLGLVRILHYTCNDIP.[3].SKCLIITKCEVV  106
RPA1D    A. thaliana            26 SEIVVQVVDLKP.[3].RYTFSANDGKTKVKAMFTASLTPEIISGKIQNLGLIRLIDFTVNDIS.[3].TKYFLVTKCEAV  102
RPA1D    A. lyrata              26 PEIVVQVVDLKP.[3].RYTFSANDGKTRVKAMFTPTLTPEIISGKIQNLGLIRLIDFTVNDIS.[3].TKYFLVNKCEAV  102
RPA1D    C. rubella             75 PEIVVQVVDLKP.[3].RYTFSANDGKTKLKAMFTASLTPEIISGKIQNLGLIRLIDFTVNDIS.[3].TKYFLVTKCEAV  151
```

B (GBS-I in DBD-C of *Hs* RPA1)

```
                                        ###          #### #     ### #               #      ###          ###
RPA1     H. sapiens            463 SSVATVVYLRK.[42].LSVNIADFQENQWVTCFQESA.[26].QNANFRSFIFRVRVKVETYNDESRIKATVMDVKPVD  598
```

C

```
                                         ###           #### #          ### #                #       ###        ###
RPA1A    M. truncatula          20 MKPLVQVISITL.[10].KYYLKLSDG.[0].VYSHSATIAAQLND.[4].GRVKEGSIVKLLDYVCPT.[3].RKIIIVH   96
RPA1A-1  B. distachyon          21 ARPIVQVLDVRC.[ 6].RWRGNVSDG.[0].VNTVPALFAGQLSA.[4].GAVRGGTILQLDEYVINN.[5].RRIIVVL   95
RPA1A-2  B. distachyon          21 ARPVVQVVDLRR.[ 4].RWRACVSDG.[0].LTSCSAMLAERLGD.[4].GVVRCGSIVQLDDYVLSD.[2].RRIVVVL   90
RPA1C-2  S. italica             25 LRPVLQVADARH.[13].RYRIDLSDG.[0].VHSQPGTLAASLNR.[4].GTLRRGSVVRVLDFVCDY.[1].RRTITVI  102
RPA1C-4  S. bicolor             61 TRPVLQLADAPQ.[10].RYRVALSDG.[1].ARLQPGMLAASLNH.[4].GALRRGTVIRVLEYFAGF.[3].QRIIIVI  138
```

Supplementary Figure S4. Amino acid Alignment of CDD predicted Generic Binding Surface I (GBS-I). Hash marks indicate the position of the conserved residues. Yellow highlighted columns show hydrophobic residues. Blue columns highlighted show polar and charged residues. Numbers in parenthesis indicate the number of omitted amino acids. (A) GBS-I found in DBD-F of RPA1B and RPA1D; (B) GBS-I found in DBD-C of human RPA1, shown for comparison; (C) GBS-I found in DBD-F of exceptional RPA1A and RPA1C. Sequences were aligned by ClustalW hosted in MEGA5.

A

```
                                                                      #     #              #   #
Homo sapiens                      463 SSVATVVYLRKENCMYQACPTQDCNKKVIDQQNGLYRCEKCDTEF.[17].ENQWVTCF 532
Xenopus laevis                    454 TSVATIVYLRKENCLYQACPSQDCNKKVIDQQNGLFRCEKCNKEF.[17].ENQWITCF 523
Danio rerio                       448 SCIATIVYIRKENCLYQACPSKDCNKKVVDQQNGMFRCEKCDKEF.[17].DNQWVTCF 517
Drosophila melanogaster           446 QCKAVVHIVKQENAFYRACPQSDCNKKVVDEGNDQFRCEKCNALF.[17].SNRWVSSF 515
Saccharomyces cerevisiae          468 SVKAAISFLKVDNFAYPACSNENCNKKVLEQPDGTWRCEKCDTNN.[17].NQLWLTLF 537
Schizosaccharomyces pombe         459 SLKGTIVYIRKKNVSYPACPAADCNKKVFDQG-GSWRCEKCNKEY.[17].GQLWLNVF 527
Neurospora crassa                 455 ALKATVVFIKQDNFAYPGCRSEGCNRKVTDMGDGTWRCEKCQINH.[17].GQLWLSCF 524
Ustilago maydis                   472 NVRATVVYIKQENLYYTACASEGCNKKVNLDHENNWRCEKCDRSY.[17].GQMWLSGF 541
```

B

```
                                                 #    #           #                 #  #        #
RPA1A   A.thaliana                489 TISFIKTDSFCYTACPLMIGDKQCNKKV.[ 4].TNRWLCDRCNQESDECDYRYLLQVQ 545
RPA1A   A.lyrata                  489 TISFIKTDSFCYTACPLMIGDKQCNKKV.[ 4].TNRWLCDRCNQESDECDYRYLLQVQ 545
RPA1A   C.rubella                 489 TVSFIKTDSFCYTACPLMIGDKQCNKKV.[ 4].TNRWLCDRCNQESDECEYRYLLQVQ 545
RPA1A   Tomato                    475 TITFIKTDTFCYTACPLMIGDRQCNKKV.[ 4].NSKWQCDRCNQEFEECDYRYLLQAQ 531
RPA1A   Strawberry                517 TISFIKTDSFCYTACPLMIGDRQCSKKV.[ 4].DR-WQCDRCNQEFEECDYRYLLQAQ 572
RPA1A   Cucumber                  469 TISFIKTDSFCYTACPLMIGDRQCNKKV.[ 4].NSKWVCDRCNQEFEDCDYRYLLQAQ 525
RPA1A   Soybean                   482 AILFIKTDTFCYTACPLMIGDRQCNKKV.[ 4].NTRWQCDRCNQEFEECDYRYLLQVQ 538
RPA1A   Barel clover              419 TISFMKTDVFCYTACPVMIGDRRCNKKV.[15].NTRWKCDTCNQEFDVCEYRYILQAQ 486
RPA1A   Castor oil plant          515 RITFVKTDTFCYTACPLMIGDRQCNKKV.[ 4].NSRWQCDRCNQEFDECDYRYLLQVQ 571
RPA1A   Grape                     469 TISFIKTDTFCYAACPLMIGDRQCNKKV.[ 4].NTRWQCDRCNQEFEDCDYRYLLQAQ 525
RPA1A   Cacao                     469 TVVFIKTDNFCYTACPLMIGDRQCNKKV.[ 4].NKRWLCDRCNQEFEECEYRYLLQVQ 525
RPA1A   California popular        515 SVSFIKTDTFCYTACPLMIGDRQCNKKV.[ 4].NSRWQCDRCNQEFDDCDYRYLLQVQ 571
RPA1A   Peach                     499 TISFIKTDSFCYTACPLMIGDRQCNKKV.[ 4].NRGWQCDRCDQEFEECDYRYLLQAQ 555
RPA1A   Maize                     504 AISFIKTDSFCYTACPNVIGDRQCGKKV.[ 4].SGNWLCDKCNQEFPECDYRYLLQLN 560
RPA1A   Sorghum                   500 TVTFIKTDPFCYTACPNVVGDRQCGKKV.[ 4].SGNWLCDKCNQEFPECDYRYLLQLN 556
RPA1A   Millet                    499 TVVFFKTDSFCYTACPNVIGERQCNKKV.[ 4].SGNWLCDKCNQEFPECDYRYLLQLQ 555
RPA1A   Rice                      502 TVIFFKNESFFYTACPNMIGDRQCNKKV.[ 4].NGNWTCDKCDREFEECDYRYLLQFQ 558
RPA1A-1 B.distachyon              475 SVIFFKNENFCYTSCPNKEGDRQCNKKV.[ 4].SGLWYCDKCNREFTECDYRYLLQLQ 531
RPA1A-2 B.distachyon              491 SIIFFKNENFCYTACPNKEGDRQCNKKV.[ 4].SGLWFCDKCNREFTECDYRYLLQLQ 547
RPA1A-1 S.moellendorffii          489 TIHFIKTDSFCYTACPLQIGDRQCSKKV.[ 4].DGTWRCERCDRTVPECDYRYMLSIQ 545
RPA1A-2 S.moellendorffii          565 TIHFIKTDSFCYTACPLQIGDRQCSKKV.[ 4].DGTWRCERCDRTVPECDYRYMLSIQ 621
RPA1A-1 P.patens                  488 TVFYIKPENFCYSACPLEVNGKQCMKKV.[ 4].DGTWRCDRCDRSVPECDYRYLLSIQ 544
RPA1A-2 P.patens                  482 TVFYIKPENFCYPACPLEVNGKQCMKKV.[ 4].DGTWRCDRCDRSVPECDYRYLLSIQ 538
```

C

```
                                                 #    #           #                        #  #        #
RPA1C   A.thaliana                588 TISFMKVENFCYTACPIMNGD-RPCS.[ 0].KKVTNNGDGTWRCEKCDKCVDECDYRY   639
RPA1C   A.lyrata                  553 TISFMKVENFCYTACPIMNGD-RPCS.[ 0].KKVTNNGDGTWRCEKCDKCVDECDYRY   604
RPA1C   C.rubella                 577 TISFMKVENFCYTACPNMNGD-RPCS.[ 0].KKVTNNGDGTWRCEKCDKCVDECDYRY   628
RPA1C   Tomato                    547 TVTFIKVDNFCYTACPLMIGD-RQCN.[ 0].KKVTNNGDGKWRCDRCDQTVDECEYRY   598
RPA1C   Strawberry                567 TVTFIKSDNCFYPACPLKTGD-RQCN.[ 0].KKVINDGDGTWRCERCDQSVQQCDYRY   618
RPA1C   Cucumber                  556 TVSFIKVDSFCYTACPIMIGD-RQCS.[ 0].KKVTNNGDGKWRCDRCDQSVDECDYRY   607
RPA1C   Soybean                   540 AVSHIKVDNFCYPGCPLKIGD-RQCN.[ 0].KKVTNNADGTWHCERCNQSIDTCDFRY   591
RPA1C   Barel clover              558 NVVFFKYDNFYYTACPNMIGD-RKCN.[ 0].KKVTDNGDKTWHCERCDTSLS-CDYRY   609
RPA1C   Castor oil plant          573 TVIYIKADNFCYTACPIMAGD-RPCS.[ 0].KKVTNNGDGKWRCEKCDQSMDECDYRY   624
RPA1C   Grape                     560 TVSFIKVDNFCYTACPIMIGD-RQCN.[ 0].KKVTNNGDGKWRCERCDQSVDDCDYRY   611
RPA1C   Cacao                     601 TIAYIKLDNFCYIACPIMNGD-RPCN.[ 0].KKVTNNGDGKWWCEKCDRSVDECDYRY   652
RPA1C   California popular        548 TVIYVKSDNFCYTACPIMSGE-RPCN.[ 0].KKVTNNGDGKWRCEKCDQSVDECDYRY   599
RPA1C   Peach                     517 TVSFIKVDSFCYSACPLMNGD-RQCS.[ 0].KKVTNNGDGKWRCDRCDQSMEECDYRY   568
RPA1C   Maize                     561 AISHLIADNFCYPACTIDVNG-RMCN.[ 0].KKVTDNGDGTWRCDKCDQSLPNCEYRY   612
RPA1C-1 Sorghum                   600 AISHLTTDNFCYPACTLEVNG-KVCN.[ 0].KKVINNGDGTWQCDKCDKSLPNCEYRY   651
RPA1C-2 Sorghum                   571 AISHVQTESFCYPACPLIFNE-KPCN.[ 0].KKVIDSGDGIWFCERCDKSSGSCEYRY   622
RPA1C-3 Sorghum                   442 AISHLKTDNFCYPACTMEVNG-RLCN.[ 0].KKVMNNGDGTWQCDKCNKSLPNFEYRY   493
RPA1C-4 Sorghum                   657 VLSHVGADNFCYQACTLELNG-KRCC.[14].RKPITVIDAIRALKTVIDAIRALKTVI   722
RPA1C-1 Millet                    699 TVSHLNTDNFCYPACTMEVNG-RQCN.[ 0].KKVINNGDGTWHCDRCDQSLPNCEYRY   750
RPA1C-2 Millet                    981 VISYVAVDKFCYPACTLELDG-KRCN.[ 0].RKVTSSGDGTWYCDRCNQCSEKCEYRY  1032
RPA1C   Rice                      598 AISHVTTESFCYPACPKLLPVGRQCN.[ 0].KKAINNGDGMWHCDRCDESFQNPEYRY   650
RPA1C   B. distachyon             566 SIFHIANDPFCYPACTMQVNG-RQCN.[ 0].KKVTNNGDGMWYCDKCEQSSPNCEYRY   617

RPA1E   A.thaliana                518 TILYLKFDNFCYTACPIMNGD-RPCS.[ 0].KKVTDNGDGTWRCEKCDKSVDECDYRY   569
RPA1E   A.lyrata                  525 TIIYMKVENFCYTACPIMNGD-RPCS.[ 0].KKVTDNGDGTWRCEKCDKSVDECDYRY   576
RPA1E   C.rubella                 609 TIIYTKFDNFCYTACPIMNGD-RPCS.[ 0].KKVTSNGDGTWTCEKCDKSVDECDYRY   660
```

Supplementary Figure S5. Amino acid alignment of the zinc-finger motif (ZFM) found with in DBD-C of RPA1. Hash marks and yellow highlighted columns indicate the position of the conserved Cysteine (C) residue. Blue highlighted residues are non-conserved amino acids. Numbers in parenthesis indicate the number of omitted amino acids. (A) ZFM from RPA1of animals and fungi, (B) ZFM from RPA1A of plants, (C) ZFM from RPA1C and RPA1E of plants, (D, see next page) ZFM from RPA1B and RPA1D of plants, (E, next page) ZFM from RPA1A/C-like sequences of unicellular green algae, (F, next page) sequences from RPA1B-like sequences of and RPA1D unicellular green algae. . Sequences were aligned by ClustalW hosted in MEGA5.

D

```
                                                       #   #             #  #       #
RPA1B    A.thaliana           449 STRAYISFIKPDQTMWYRACKTCNKKVTEAMDSGYWCESCQKKDQECSLRYIMAVKVSD 507
RPA1B    A.lyrata             449 SIRAYISFIKPDQTMWYRACKTCNKKVTEAMDSGYWCEGCQKKDQECSLRYIMAVKVSD 507
RPA1B    C.rubella            452 STRAYISFIKPDQTMWYRACKTCNKKVTEAMDSGYWCEGCQKKDQECSLRYIMAVKVSD 510
RPA1B    Tomato               460 SLKAYISFIKPDQTMWYRACKTCNKKVTEAFGSGYWCEGCQKNDAECSLRYIMALRVSD 518
RPA1B    Strawberry           455 SVKAYIGPIKSDQTLWYRACKVCNKKVTEGDG-GYWCEGCQKIAEECSLRYILLARAMD 512
RPA1B-1  Cucumber             449 SIRAYVSFIKPDQTMWYRACKTCNKKVTEAIGSGYWCDNCQKNDEECSLRYIMVVRVSD 507
RPA1B-2  Cucumber             406 SIRAYVSFIKPDQTMWYRACKTCNKKVTEAIGSGYWCDNCQKNDEECSLRYIMVVRVSD 464
RPA1B-1  Soybean              467 SLRGHITFIKPDQAMWYRACKTCNKKVTESFGSGYWCDGCQKSDEQCSLRYIMVAKVSD 525
RPA1B-2  Soybean              466 SLRGHISFIKPDQAMWYRACKTCNKKVTESVGSGYLCDGCQKSDEQCSLRYIMVAKVSD 524
RPA1B    Castor oil plant     468 SIRAYISFIKPDQSMWYRACKTCNKKVTEAIGGGYWCEGCQKNDAECSLRYIMVVKVSD 526
RPA1B    Grape                470 SIRAYISFIKPDQTMWYRACKTCNKKVTDAIGSGYWCEGCQKNDDECSLRYIMVVKVSD 528
RPA1B    Cacao                522 SIKAFISLIRPEQAMWYRACKSCNKKVTEAVGSGYWCEGCQKNDEECSLRYIMVSKISD 580
RPA1B    California popular   449 SIRAYISFIKPDQTMWYRACKTCNKKVTDALGGGYWCEGCQKNDAECSLRYIMVVKVSD 507
RPA1B    Peach                454 SVKAFISSIRPDQALWYRACKTCNKKVTEAIGSGYWCEACQKNDEECSLRYILVARVTD 512
RPA1B-1  Maize                470 SLYAIISHIKPDQNMWYRACTTCNKKVTEAFGSGYWCEGCQKNYSECSLRYIMVIKLSD 528
RPA1B-2  Maize                465 SLYATISHIKPDQNMWYRACKTCNKKVTETFGSGYWCEGCQKNDSECSLRYIMVIKVSD 523
RPA1B    Sorghum              470 SLNAIISHIKPDQNMWYRACKTCNKKVTEAFGSGYWCEGCQKNDSECSLRYIMVLKISD 528
RPA1B    Millet               470 SLNAIISHIKPDQNMWYRACKTCNKKVTEAVGGGYWCEGCNKNDAECSLRYIMVIKVSD 528
RPA1B    Rice                 477 SLNAYISLIKPDQTMWYRACKTCNKKVTEAIGSGYWCEGCQKNDAECSLRYIMVIKVSD 535
RPA1B    B.distachyon         482 SLNAYISHIKPDQTMWYRACKTCNKKVTEAVGSGYWCEGCQKNYEECMLRYIMAIKVSD 540
RPA1B-1  S.moellendorffii     414 TVRACISYIKPDQTMWYTACSTCNRKVSEDSSR-FWCEACQRHFDTASRRYIMLAKLTD 471
RPA1B-2  S.moellendorffii     414 TVRACISYIKPDQTMWYTACSTCNRKVSEDSSR-FWCEACQRHFDTASRRYIMLAKLTD 471
RPA1B    P.patens             300 NVRAYISFIKPDQAMWYLACQTCNRKVVEQSSSSYWCEGCQNHYDKCSRRYIMQAKLSD 358

RPA1D    A.thaliana           473 STRAYISFIKPDQTMWYQACKTCNKKVTEALDSGYWCEGCQRKYEECSLRYIMAVKVTD 531
RPA1D    A.lyrata             475 STRAYISFIKPDQTMWYQACKTCNKKVTEALDSGYWCEGCQKKYEECSLRYIMAVKVTD 533
RPA1D    C.rubella            524 STRAYVSFIKPDQTMWYQACKTCNKKVTEALDSGYWCEGCQKKYEECSLRYIMAVKVTD 582
```

E

```
                                                        #           #                  #  #
RPA1A C. reinhardtii       312 NVSAVLD.[ 8].VVYPSCPHDFNGRPCQKKMMDVGGGN----WNCDRCQ-FSTENPAWRYLVSLSACD 377
RPA1A C. subellipsoidea    493 QIVCTIT.[ 6].IAYPACTLQYNGKQCNKKVTDSGGGDGPNRWWCERCS-AACE-AEYRYMLNLNIED 559
RPA1C M. pusilla           516 QCRCHVT.[ 8].CFYPACPLRNGERMCQKKLRYDEAMG---TWNCERHAGEHVPNCEWRYIINMTVAD 583
RPA1C V. cateri           537 TVSAVVD.[13].VVYPSCPHDFNGRPCQKKMMDIGGGN----WNCERCN-YSTENPAWRYLVSMSACD 607
RPA1E O. lucimarinus      492 SCCGIIK.[ 8].NFYPACPLLNGERTCQKKLRKDDSTG---EWKCERHAGEKIEAADWRYMFSMVCMD 559
```

F

```
RPA1B C. reinhardtii       494 QNVTACVAMINNDDKNIFYLANPE--NGRKVVDQGGGRFWSEADSKVVEKPEHRYLLSVRLADHTGETN 560
RPA1B C. subellipsoidea    322 HTVIATVANID-SQQSLYYEACPD--NNRKVVKQGEGW-FCEYDQQTYMAMVRRYVMLANCVDASGDCL 386
RPA1B M. pusilla           388 GILGCATVVLVKPDQPMYYCACPEEGNNKKVVEESPGKWYCEATQKTYDSCRRRYILRLKVSDHAGGGW 456
RPA1B V. cateri           349 QAVTAYIAMVN-SELQMYYLANPE--NGRKVVDQGGGR-WAEADGRVVERPEHRYVLSVKLADHTGEAV 413
RPA1B O. lucimarinus      422 AWV-CAHTVMCKPDQTMYYTATPEEGNNKKVIE-SDGKWYCEANGQTYDTCERRYIMRFKAQDSSEGAW 488
```

Supplementary Figure S5, continued. Amino acid alignment of the zinc-finger motif (ZFM) found in RPA1 DBD-C. Hash marks and yellow highlighted columns indicate the position of the conserved Cysteine (C) residue. Blue highlighted residues are non-conserved amino acids. Green highlighted residues are non-conserved Cysteine (C) amino acids in the DBD-C region where ZFM is found. Numbers in parenthesis indicate the number of omitted amino acids. (D) ZFM from RPA1B and RPA1D of plants, (E) ZFM from RPA1A/C-like sequences of unicellular green algae, (F) sequences from RPA1B-like sequences of unicellular green algae. Sequences were aligned by ClustalW hosted in MEGA5.

A

```
RPA1C    A.thaliana         810 SCNVCRSNSHVSANCP---------------------------------------------------------------------------------------- 825
RPA1C    A.lyrata           775 SCNACGSNSHVSANCP---------------------------------------------------------------------------------------- 790
RPA1C    C.rubella          799 SCNACGSNGHVSANCP---------------------------------------------------------------------------------------- 814
RPA1C    Tomato             812 SCNSCGGTGHSASNCP.[29].----------------------ECYKCHQYGHWARDCP-------------------------------------------- 872
RPA1C    Strawberry         794 SCNICGGTSHNSLNCP.[22].----------------------ECYKCHQPGHWASDCP-------------------------------------------- 847
RPA1C    Cucumber           785 YCNSCGGSGHSSTNCP.[28].----------------------ECFKCHQTGHWARDCP-------------------------------------------- 844
RPA1C    Soybean            767 SCTNCGVSGHSSALCP.[30].ECYKCHQSGHYARDCP.[20].ECFKCHQTGHWARDCP-------------------------------------------- 864
RPA1C    Barel clover       784 SCSNCGGSDHSSAQCL.[26].KCYKCQQPGHWASNCP.[17].NCYKCNQPGHWANNCP-------------------------------------------- 874
RPA1C    Castor oil plant   800 SCISCGATSHSSANCP.[29].----------------------ECYKCHQVGHWARDCP- ------------------------------------------ 860
RPA1C    Grape              792 SCNSCGGTGHSSSNCP.[29].----------------------ECYKCHQFGHWARDCP-------------------------------------------- 852
RPA1C    Cacao              824 FCNSCGVTGHSSTNCP.[29].----------------------ECYKCHQSGHWAKDCP-------------------------------------------- 884
RPA1C    California popular 780 SCNSCGATSHSSANCP.[24].----------------------ECYKCHQVGHWARDCP-------------------------------------------- 835
RPA1C    Peach              749 SCNSCGDAGHSSMNCP.[29].----------------------DCYKCHQPGHWARDCP-------------------------------------------- 809
RPA1C    Maize              787 TCVSCGSSGHNVQNCP.[24].----------------------LCFKCNQPGHFANSCP-------------------------------------------- 842
RPA1C-1  Sorghum            825 TCMSCGSSGHNAQNCP.[27].----------------------PCFKCNQIGHFADSCP-------------------------------------------- 883
RPA1C-2  Sorghum            814 --------------------------------------------PCFKCHQPGHWSKECP-------------------------------------------- 829
RPA1C-3  Sorghum            647 --------------------------------------------ACFKCNQPGHWSKDCL-------------------------------------------- 662
RPA1C-1  Millet             924 TCMGCGSSGHNAQSCP.[30].----------------------RCFRCNQPGHFANACP-------------------------------------------- 982
RPA1C-2  Millet            1192 --------------------------------------------LCFRCNRPGHWAKDCF-------------------------------------------- 1207
RPA1C    Rice               815 TCSICGANGHSAQICH.[29].----------------------ECYKCKQPGHYARDCP.[ 7].ECFKCKQPGHFSRDCP.[ 7].ECFKCKQPGHFARDCP 921
RPA1C    B.distachyon       785 TCSVCGSNGHNAQNCP.[31].----------------------LCFKCNQPGHFSRDCP.[18].LCFKCNQPGHYSRDCP.[17].LCFKCNQPGHYARDCP 914

RPA1E A.thaliana            734 SCNVCGNSGHVSAKCP---------------------------------------------------------------------------------------- 749
RPA1E A.lyrata              752 SCNVCGNSGHVSANCP---------------------------------------------------------------------------------------- 767
RPA1E C.rubella             830 SCNVCGNSGHVSANCP---------------------------------------------------------------------------------------- 845
```

B

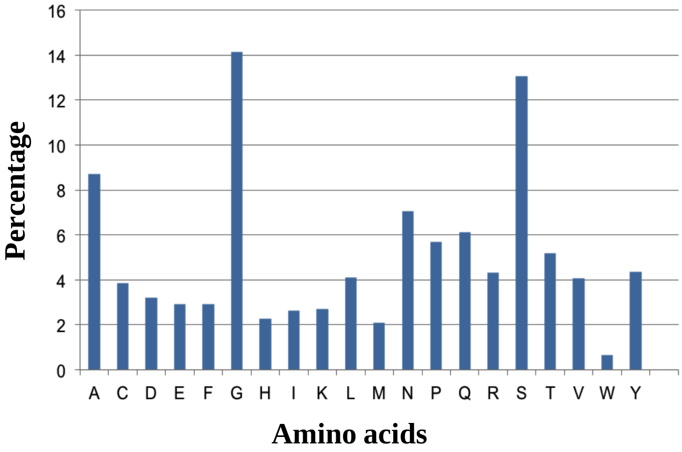

C

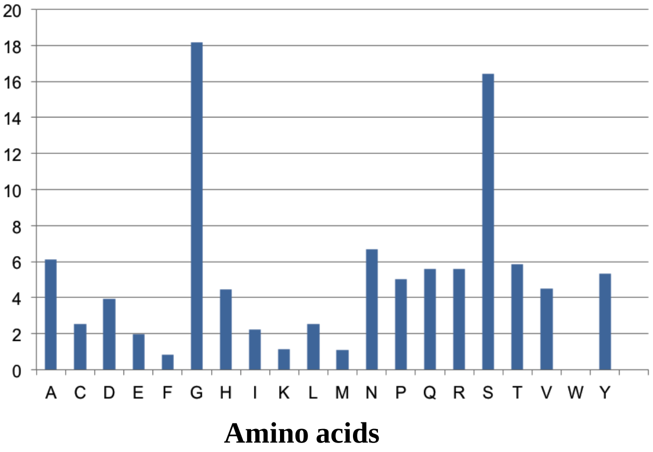

Supplementary Figure S6. (A) Amino acid alignment of the zinc-finger (zinc-knuckle) motif (ZFM) found within the C-terminal extension region of RPA1C and RPA1E. Yellow highlighted columns show the position of conserved Cysteine (C) and Histidine (H) residues. Numbers in parenthesis indicate the number of omitted amino acids. Sequences were aligned by ClustalW hosted in MEGA5. (B) & (C) Average amino acid composition of the C-terminal extension region of RPA1C and RPA1E, respectively. Twenty RPA1C and three RPA1E plant sequences were used for the analysis. For the list of these plants and their individual amino acid composition see Supplementary Table S3. Analysis was conducted using MEGA5.
